# Supplementary material for: Semantic priming supports infants’ ability to learn names of unseen objects
Source: PLoS One. 2025 Apr 23;20(4):e0321775. doi: 10.1371/journal.pone.0321775 (PMC12017536; doi:10.1371/journal.pone.0321775)
Supplement: S1 Appendix — (DOCX) [file pone.0321775.s001.docx]

Appendix 1. Augmented MacArthur Short Form Vocabulary Checklist: Level II

**Please provide your email address (to receive your payment)**:

- Email (4) __________________________________________________

**Child's First and Last Name**
(we need this to ensure that survey responses are matched correctly with the experimental data; after that, the data will be anonymized):

________________________________________________________________

**Child's Birthdate** **(example: December 15, 2012)**

________________________________________________________________

**Which language(s), other than English, is/are spoken at home? If English is the only language spoken, please write n/a.**

________________________________________________________________

**What percentage of the time does your child hear this language?**

________________________________________________________________

**Vocabulary checklist**
For words that your child understands but does not yet say, mark the first column (understands). For words that your child understands but also says, mark the second column (understands and says). If your child uses a different pronunciation of a word, mark it anyway.

|  | Understands (1) | Understands and says (2) |
| --- | --- | --- |
| hat (22) |  |  |
| sock (25) |  |  |
| jacket (90) |  |  |
| dog (6) |  |  |
| horse (91) |  |  |
| cat (92) |  |  |
| banana (93) |  |  |
| apple (95) |  |  |
| orange (94) |  |  |
| truck (97) |  |  |
| car (11) |  |  |
| bus (96) |  |  |
| choo choo (1) |  |  |
| meow (2) |  |  |
| ouch (3) |  |  |
| uh oh (4) |  |  |
| bird (5) |  |  |
| duck (7) |  |  |
| kitty (8) |  |  |
| lion (9) |  |  |
| mouse (10) |  |  |
| stroller (12) |  |  |
| ball (13) |  |  |
| book (14) |  |  |
| doll (15) |  |  |
| bread (16) |  |  |
| candy (17) |  |  |
| cereal (18) |  |  |
| cookie (19) |  |  |
| juice (20) |  |  |
| toast (21) |  |  |
| pants (23) |  |  |
| shoe (24) |  |  |
| eye (26) |  |  |
| head (27) |  |  |
| leg (28) |  |  |
| nose (29) |  |  |
| tooth (30) |  |  |
| chair (31) |  |  |
| couch (32) |  |  |
| kitchen (33) |  |  |
| table (34) |  |  |
| television (35) |  |  |
| blanket (36) |  |  |
| bottle (37) |  |  |
| cup (38) |  |  |
| dish (39) |  |  |
| lamp (40) |  |  |
| radio (41) |  |  |
| spoon (42) |  |  |
| flower (43) |  |  |
| home (44) |  |  |
| moon (45) |  |  |
| outside (46) |  |  |
| plant (47) |  |  |
| rain (48) |  |  |
| rock (49) |  |  |
| water (50) |  |  |
| babysitter (51) |  |  |
| girl (52) |  |  |
| grandma (53) |  |  |
| mommy (54) |  |  |
| bath (55) |  |  |
| don't (56) |  |  |
| hi (57) |  |  |
| night night (58) |  |  |
| patty cake (59) |  |  |
| please (60) |  |  |
| wait (61) |  |  |
| break (62) |  |  |
| feed (63) |  |  |
| finish (64) |  |  |
| help (65) |  |  |
| jump (66) |  |  |
| kick (67) |  |  |
| kiss (68) |  |  |
| push (69) |  |  |
| sing (70) |  |  |
| smile (71) |  |  |
| night (72) |  |  |
| today (73) |  |  |
| all gone (74) |  |  |
| big (75) |  |  |
| broken (76) |  |  |
| dark (77) |  |  |
| fast (78) |  |  |
| hurt (79) |  |  |
| pretty (80) |  |  |
| soft (81) |  |  |
| I (82) |  |  |
| me (83) |  |  |
| how (84) |  |  |
| who (85) |  |  |
| away (86) |  |  |
| out (87) |  |  |
| other (88) |  |  |
| some (89) |  |  |
