# Supplementary material for: Semantic priming supports infants’ ability to learn names of unseen objects
Source: PLoS One. 2025 Apr 23;20(4):e0321775. doi: 10.1371/journal.pone.0321775 (PMC12017536; doi:10.1371/journal.pone.0321775)
Supplement: S2 Appendix — (DOCX) [file pone.0321775.s002.docx]

[CHS](https://childrenhelpingscience.com/)

[Home](https://childrenhelpingscience.com/)

[Experimenter](https://childrenhelpingscience.com/exp/studies/)

[Studies](https://childrenhelpingscience.com/studies/)

[FAQ](https://childrenhelpingscience.com/faq/)

[The](https://childrenhelpingscience.com/scientists/)

[Scientists](https://childrenhelpingscience.com/scientists/)

[Resources](https://childrenhelpingscience.com/resources/)

[My](https://childrenhelpingscience.com/account/manage/)

[Account](https://childrenhelpingscience.com/account/manage/)

[My Past](https://childrenhelpingscience.com/studies/history/)

[Studies](https://childrenhelpingscience.com/studies/history/)

[Logou](https://childrenhelpingscience.com/logout/)t

One reason we are developing Internet-based experiments is to represent a more diverse group of families in our research. Your answers to these questions will help us understand what audience we reach, as well as how factors like speaking multiple languages or having older siblings affect children's learning.


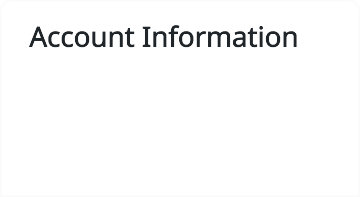


[Change your login credentials and/or nickname.](https://childrenhelpingscience.com/account/manage/)


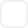

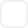

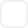

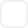

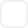

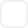

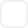

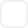


| [Tell us more about yourself.](https://childrenhelpingscience.com/account/demographics/) |
| --- |
| [Add or edit participant information.](https://childrenhelpingscience.com/account/children/) |
| [See all available studies.](https://childrenhelpingscience.com/studies/) |
| [Edit when you can be contacted.](https://childrenhelpingscience.com/account/email/) |

Even if you allow your study videos to be published for scientific or publicity purposes, your demographic information is never published in conjunction with your video.

What country do you live in? United States of America

What state do you live in?

---------

How would you describe the area where you live?

What racial group(s) does your family identify with/belong to? Please select any that apply to someone in your children's immediate family.

White

Hispanic, Latino, or Spanish origin Black or African American

Asian

American Indian or Alaska Native Middle Eastern or North African

Native Hawaiian or Other Pacific Islander Another race, ethnicity, or origin

Please select the appropriate responses for everyone in your children's immediate family.

How many children do you have?

Enter as a comma-separated list: YYYY-MM-DD, YYYY-MM-DD,

...

How many parents/guardians do your children live

with?

If the answer varies or needs more explanation, you can tell us more below.

What is your age?

What is your gender?

What is the highest level of education you've

completed?

What is your approximate family yearly income (in US

dollars)?

How did you hear about Lookit?

How did you hear about Lookit?

Anything else you'd like us to know?

Anything else you'd like us to know?

Save


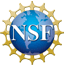
This material is based upon work supported by the National Science Foundation (NSF) under Grants 1429216, 1823919, and 2209756; the Center for Brains, Minds and Machines (CBMM), funded by NSF STC award CCF- 1231216, and by an NSF Graduate Research Fellowship under Grant No.

1122374. Any opinion, findings, and conclusions or recommendations expressed in this material are those of the authors(s) and do not necessarily reflect the views of the National Science Foundation.

[Privacy](https://childrenhelpingscience.com/privacy/) [Contact us](https://childrenhelpingscience.com/contact_us/) Connect: [
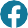
](https://www.facebook.com/lookit.mit.edu)
