# Supplementary material for: Semantic priming supports infants’ ability to learn names of unseen objects
Source: PLoS One. 2025 Apr 23;20(4):e0321775. doi: 10.1371/journal.pone.0321775 (PMC12017536; doi:10.1371/journal.pone.0321775)
Supplement: S3 Appendix — *Source of images in depicted here: https://unsplash.com. Actual stimuli may differ (all stimuli are available on OSF). (DOCX) [file pone.0321775.s003.docx]

**Appendix 3. Visual and auditory stimuli in Experiments 1 and 2**

**Semantic Priming condition**

| Semantic neighborhood | Priming Phase | | | | | Test Phase |
| --- | --- | --- | --- | --- | --- | --- |
|  | Familiar word-object 1 | Familiar word-object 2 | Familiar word-object 3 | Novel word | |  |
| Fruits: object images | 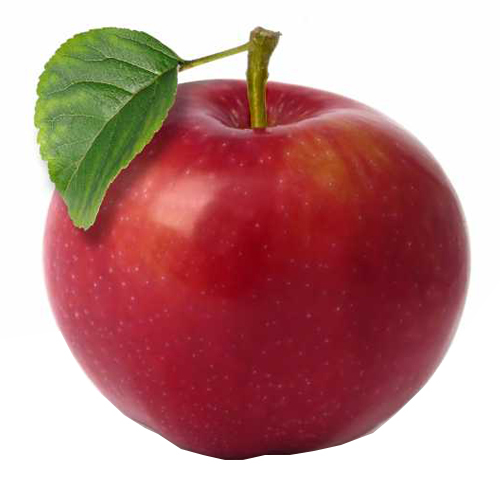 | 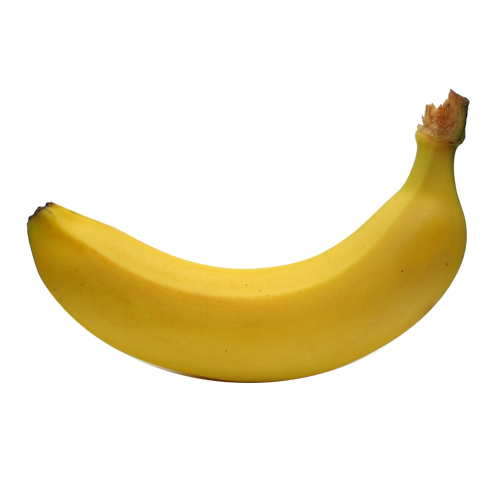 | 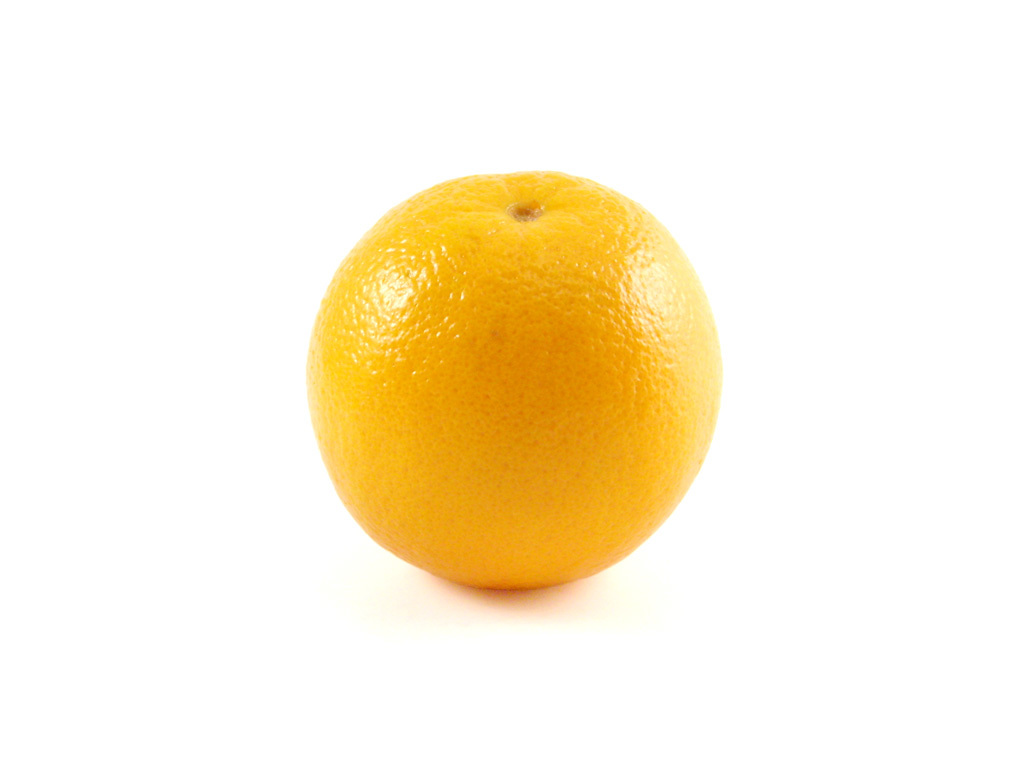 |  |  | 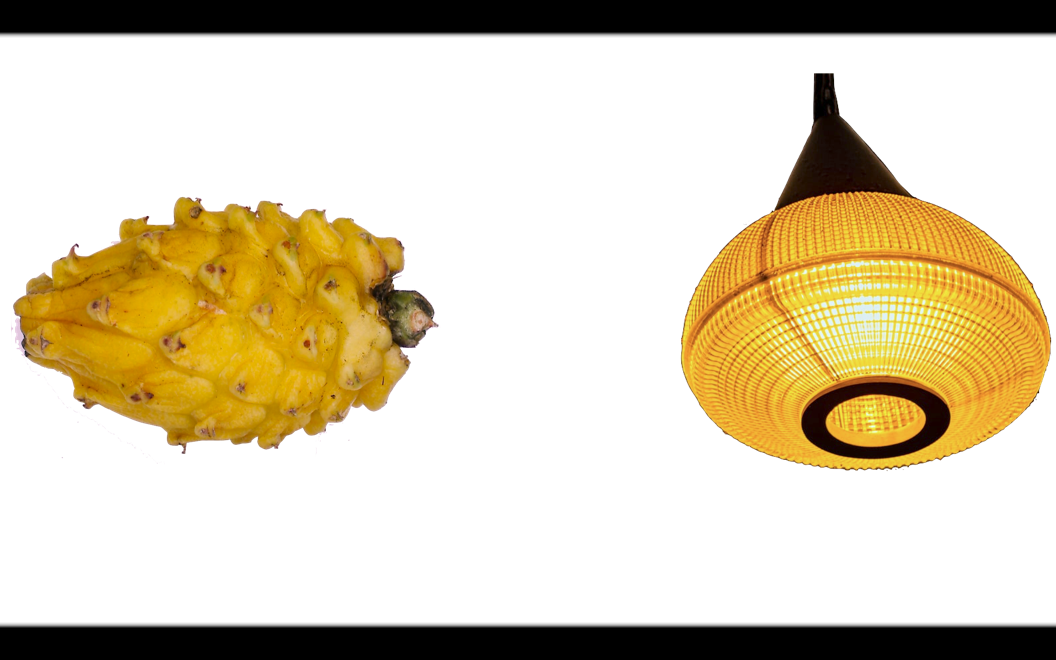 |
| Fruits: auditory stream | Ooh! Look! An apple! Do you see the apple? | Ooh! Look! A banana! Do you see the banana? | Ooh! Look! An orange! Do you see the orange? | Ooh! A modi! That’s a nice modi! I like modis! | Let’s play a game! Let’s find the modi! | Now look! Where is the modi? [2 s delay] Can you find the modi? |
| Vehicles: object images | 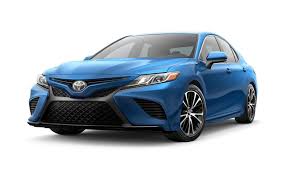 | 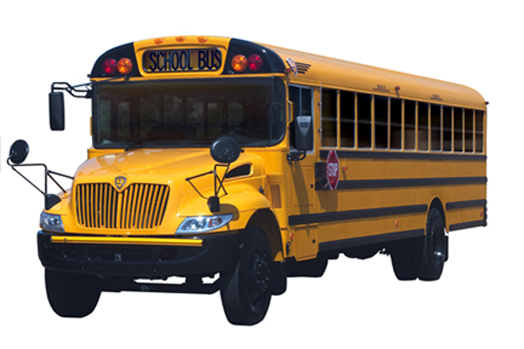 | 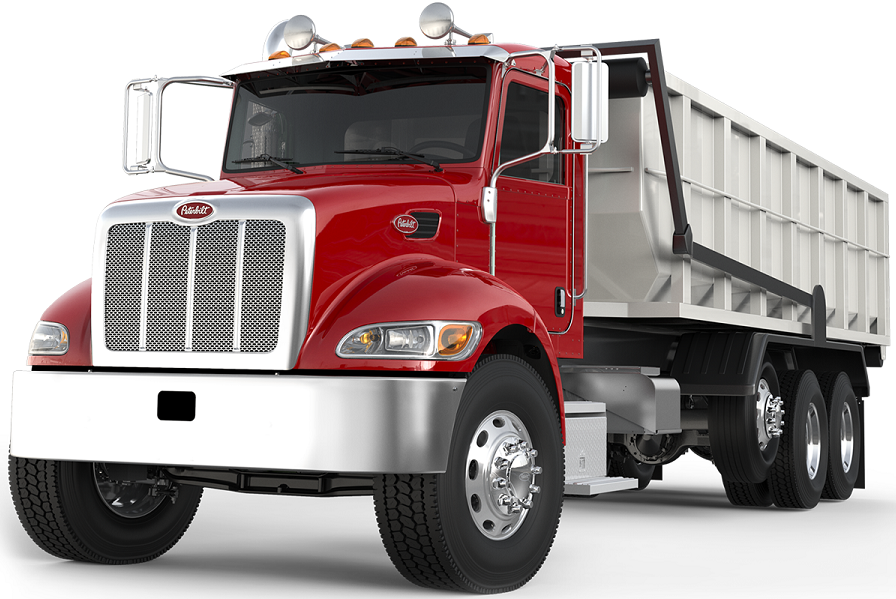 |  |  | 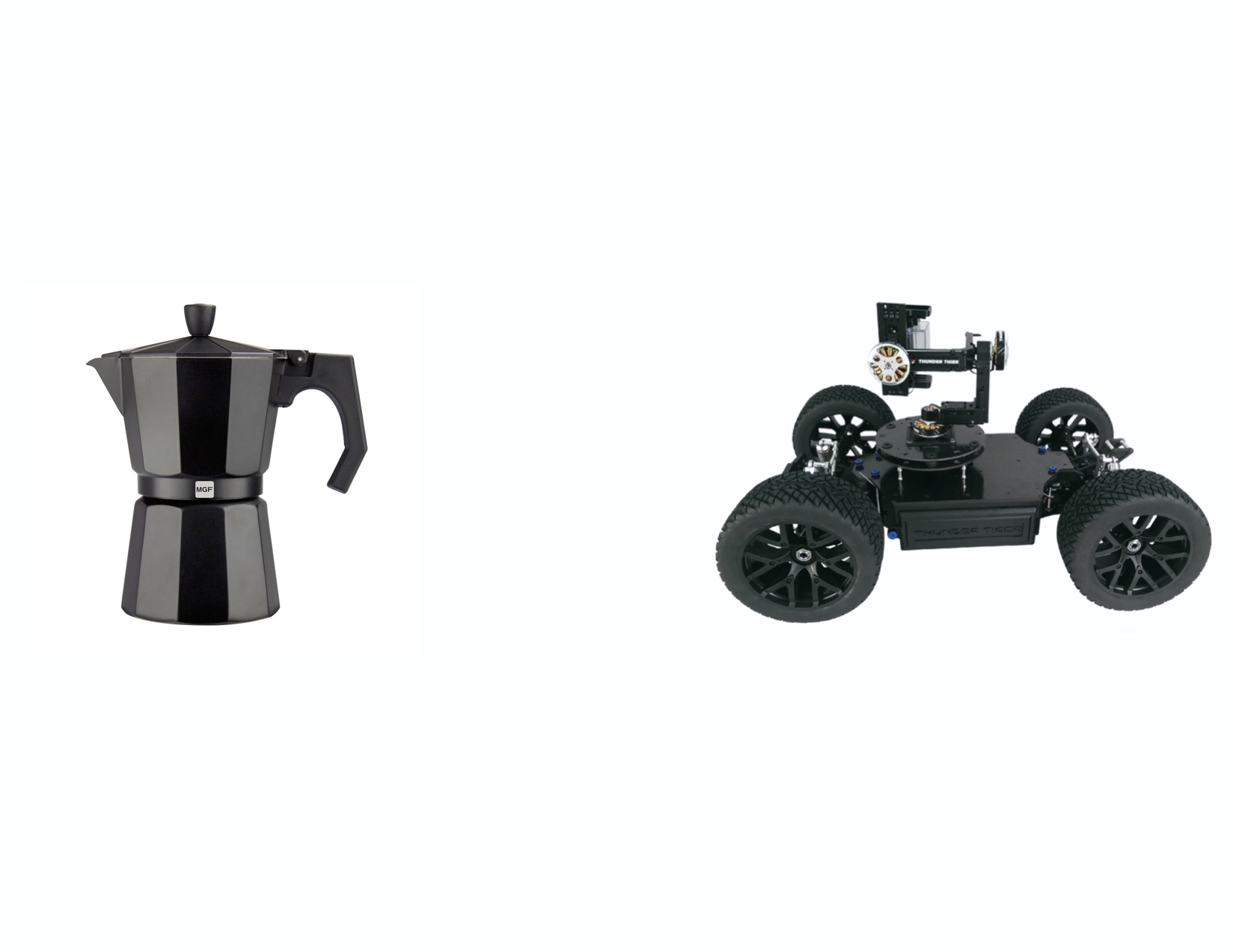 |
| Vehicles: auditory stream | Ooh! Look! A car! Do you see the car? | Ooh! Look! A bus! Do you see the bus? | Ooh! Look! A truck! Do you see the truck? | Ooh! A dax! That’s a nice dax! I like daxes! | Let’s play a game! Let’s find the dax! | Now look! Where is the dax? [2 s delay] Can you find the dax? |
| Animals: object images | 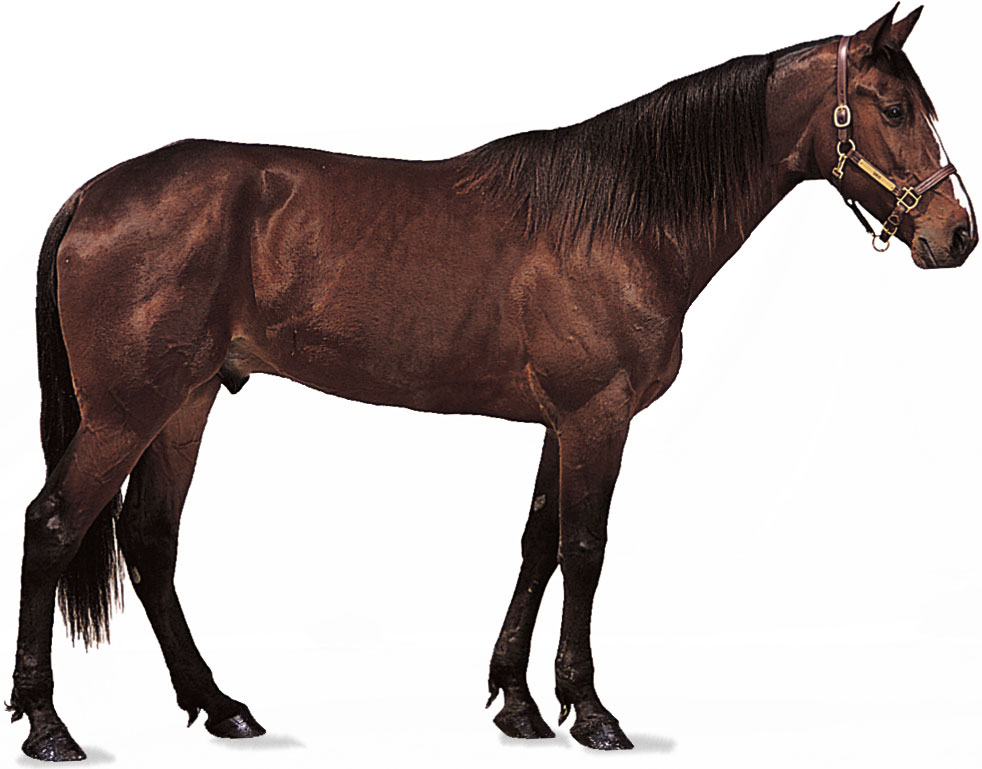 | 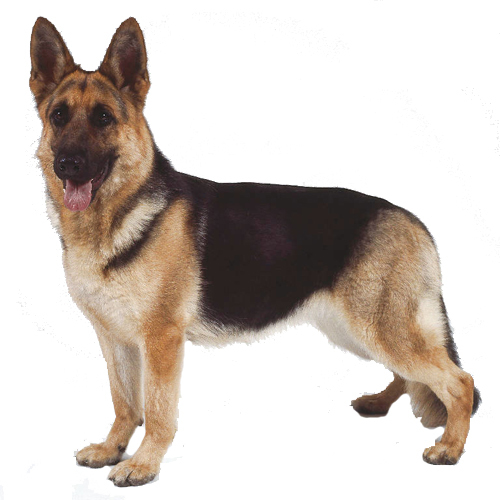 | 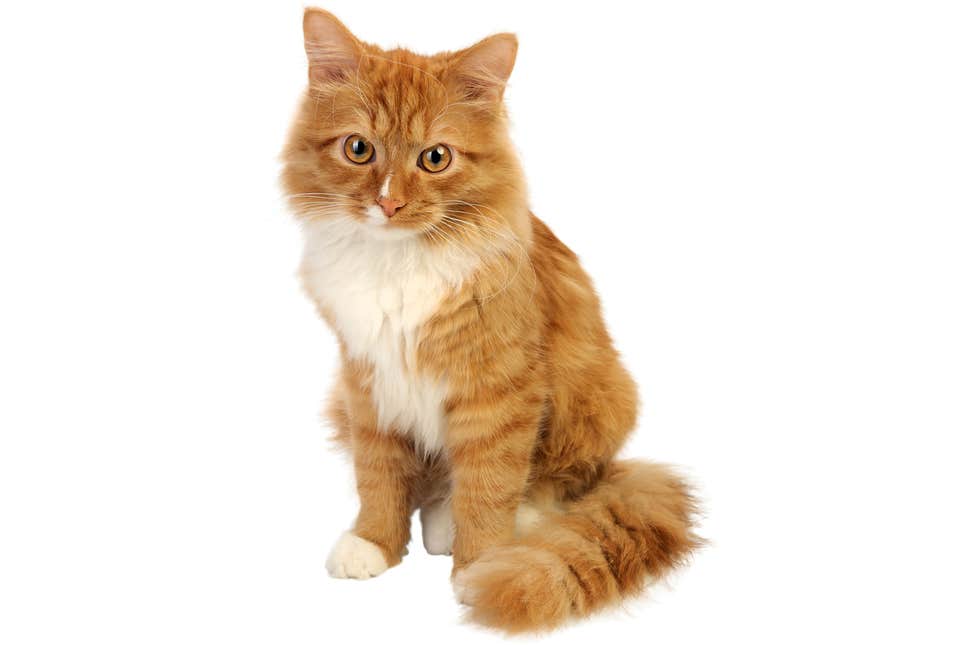 |  |  | 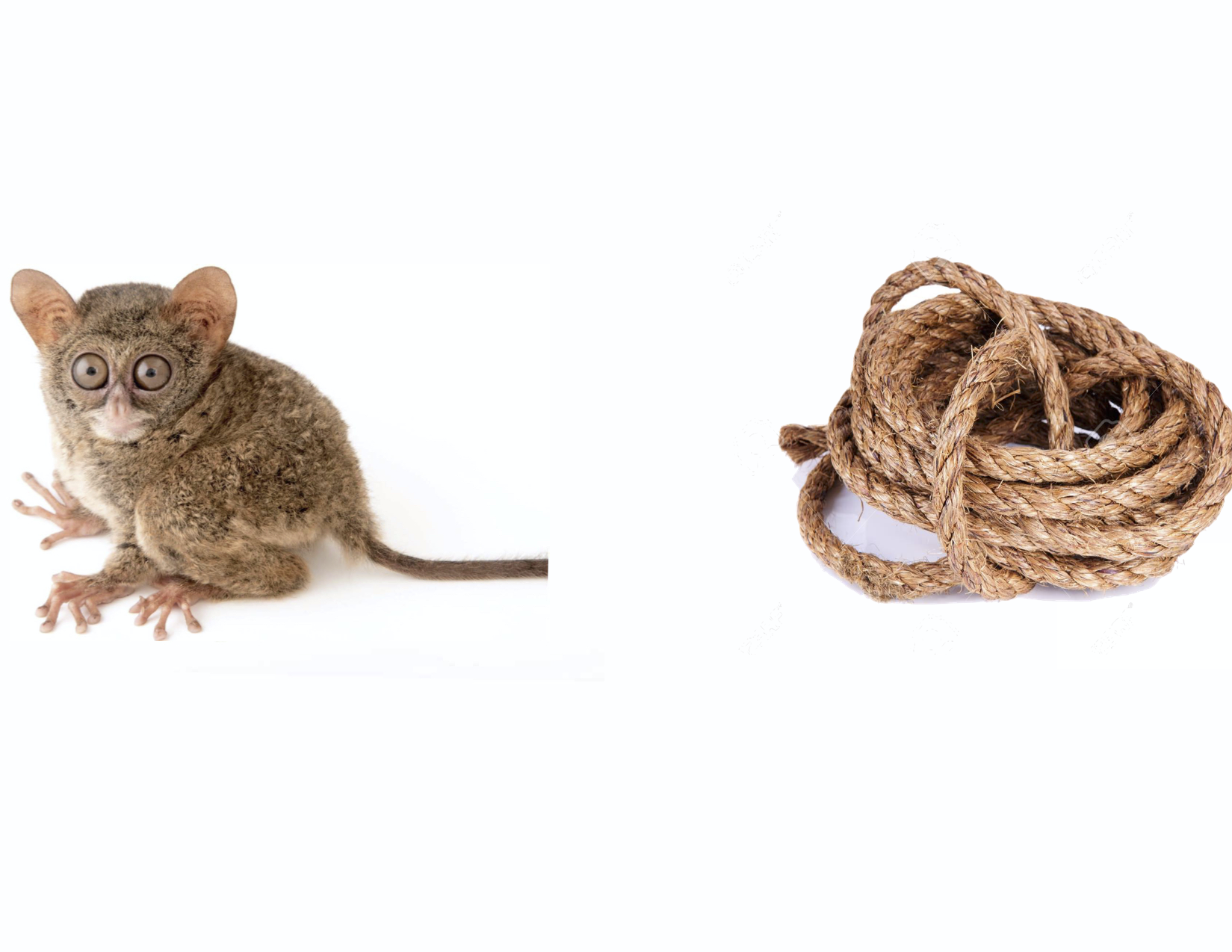 |
| Animals: auditory stream | Ooh! Look! A horse! Do you see the horse? | Ooh! Look! A dog! Do you see the dog? | Ooh! Look! A cat! Do you see the cat? | Ooh! A wug! That’s a nice wug! I like wugs! | Let’s play a game! Let’s find the wug! | Now look! Where is the wug? [2 s delay] Can you find the wug? |
| Clothing: object images | 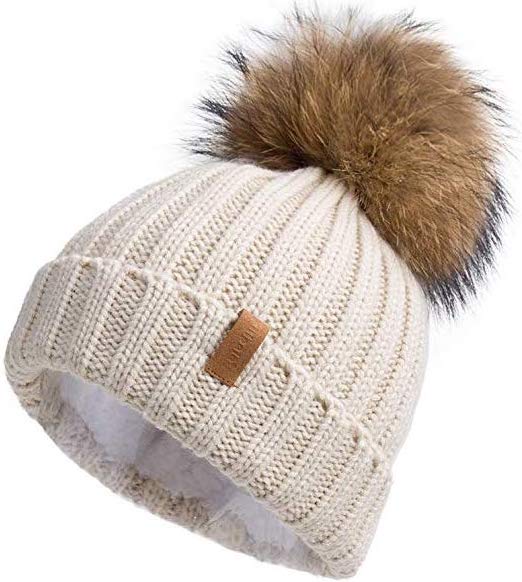 | 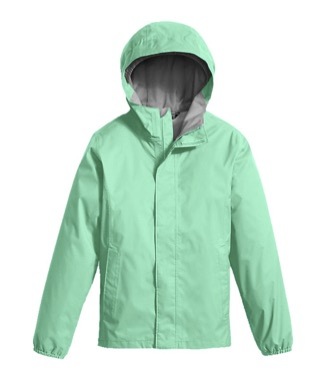 | 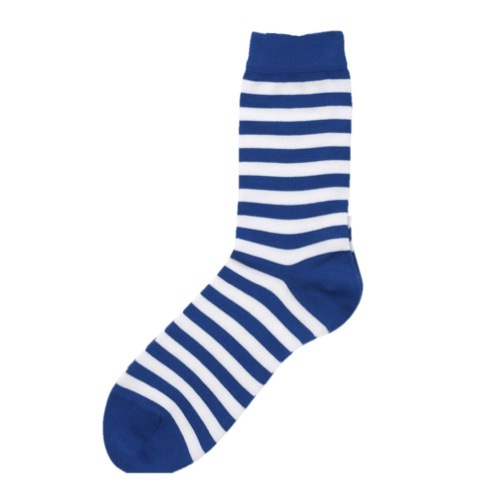 |  |  | 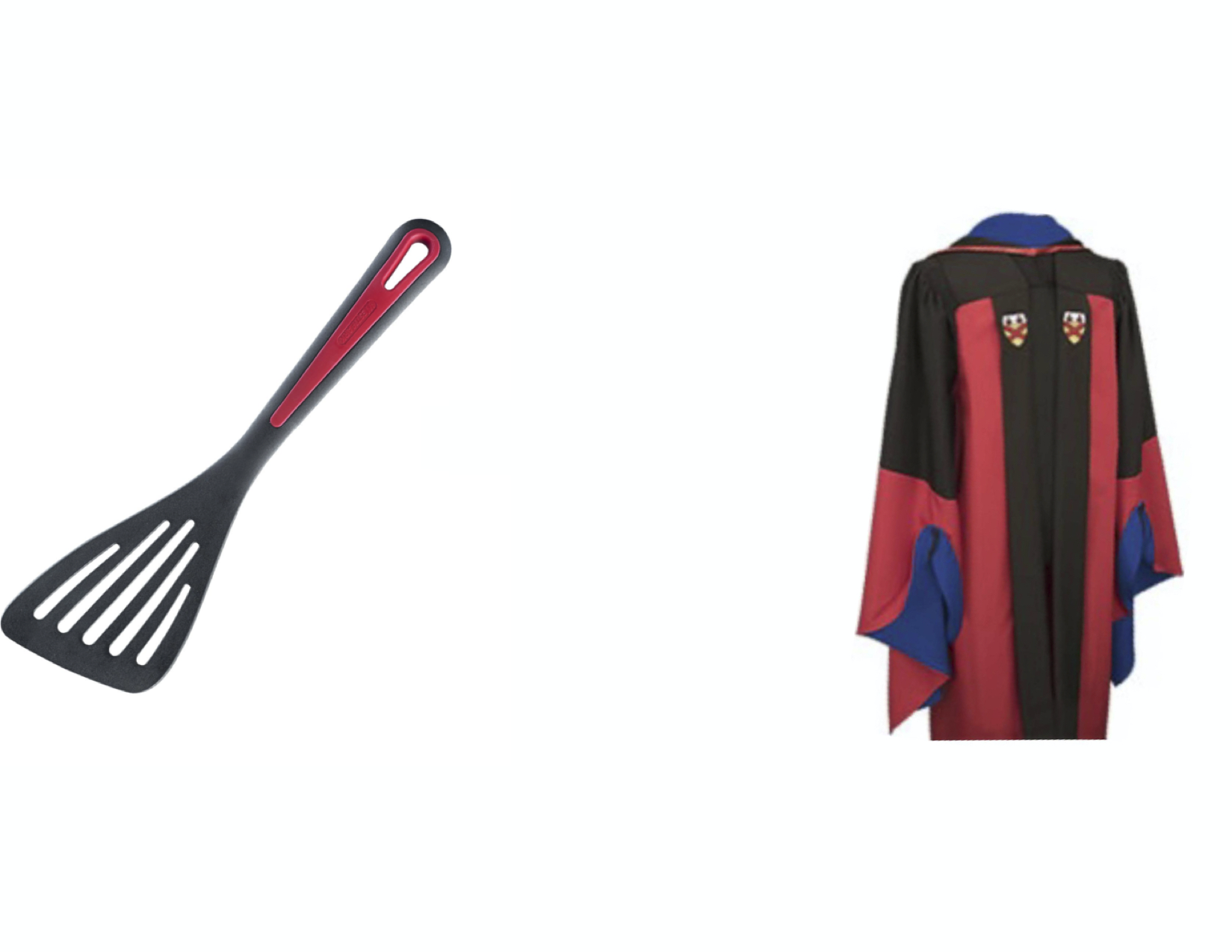 |
| Clothing: auditory stream | Ooh! Look! A hat! Do you see the hat? | Ooh! Look! A jacket! Do you see the jacket? | Ooh! Look! A sock! Do you see the sock? | Ooh! A blicket! That’s a nice blicket! I like blickets! | Let’s play a game! Let’s find the blicket! | Now look! Where is the blicket? [2 s delay] Can you find the blicket? |

**Switch Word condition**

| Semantic neighborhood | Priming Phase | | | | | Test Phase |
| --- | --- | --- | --- | --- | --- | --- |
|  | Familiar word-object 1 | Familiar word-object 2 | Familiar word-object 3 | Novel word | |  |
| Fruits: object images | 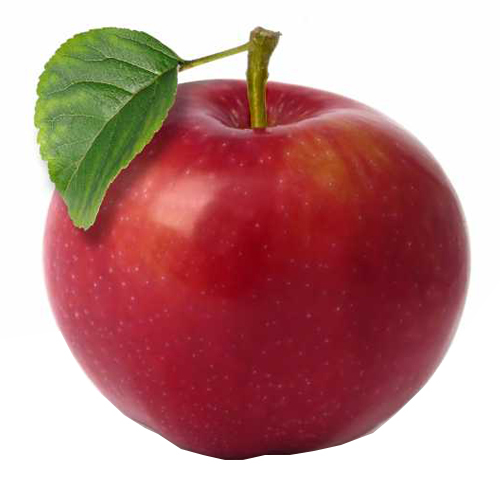 | 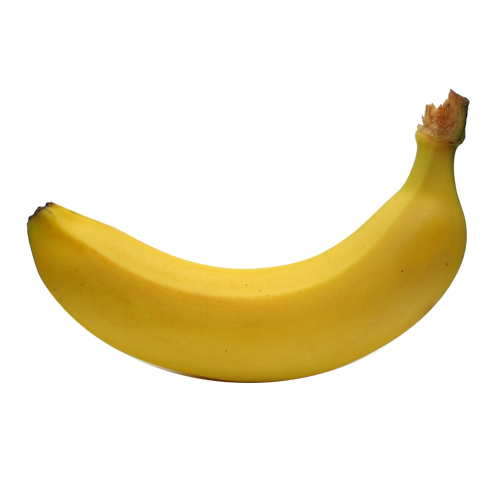 | 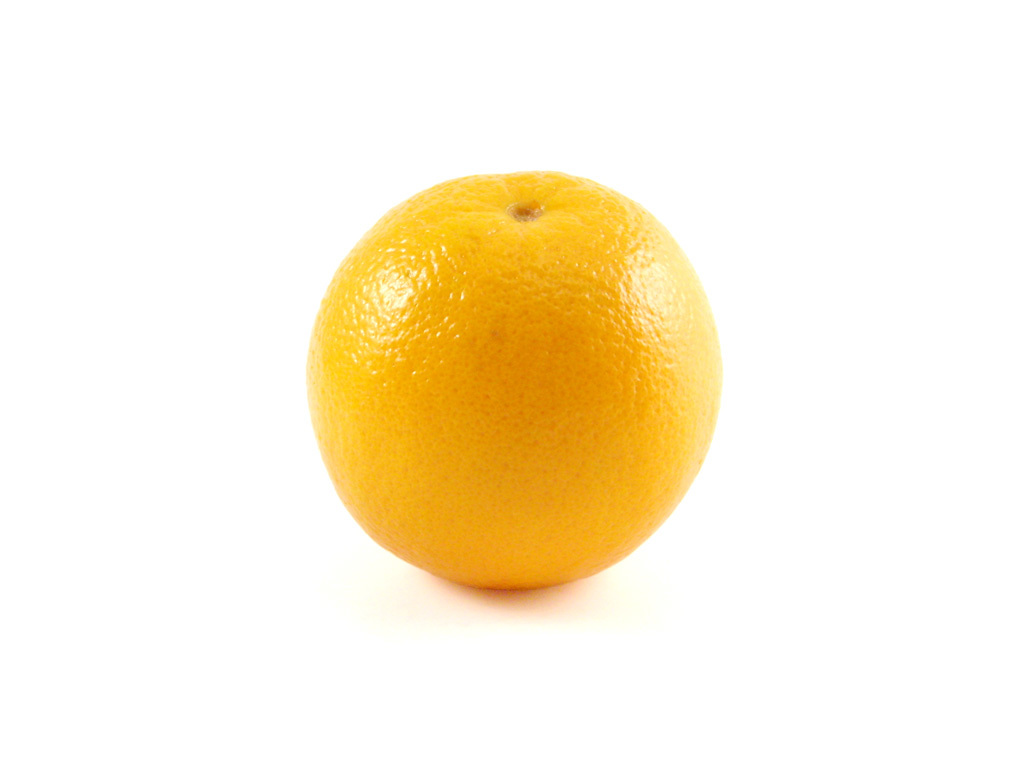 |  |  | 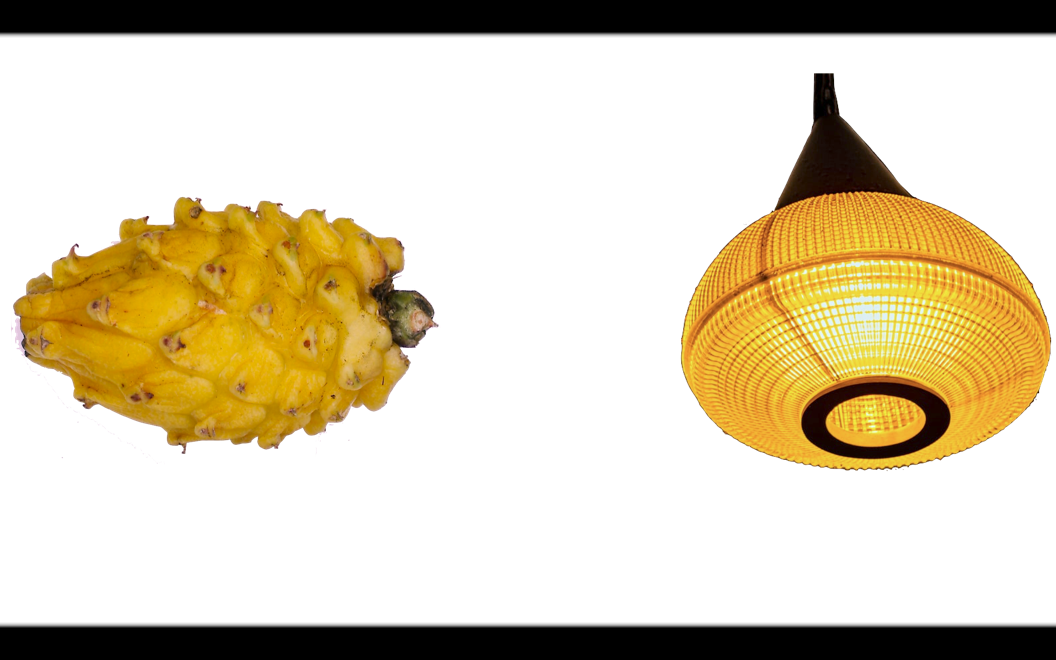 |
| Fruits: auditory stream | Ooh! Look! An apple! Do you see the apple? | Ooh! Look! A banana! Do you see the banana? | Ooh! Look! An orange! Do you see the orange? | Ooh! A modi! That’s a nice modi! I like modis! | Let’s play a game! Let’s find the modi! | Now look! Where is the d*anu*? [2 s delay] Can you find the *danu*? |
| Vehicles: object images | 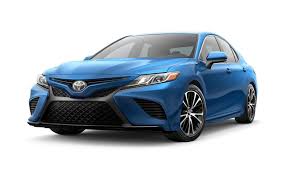 | 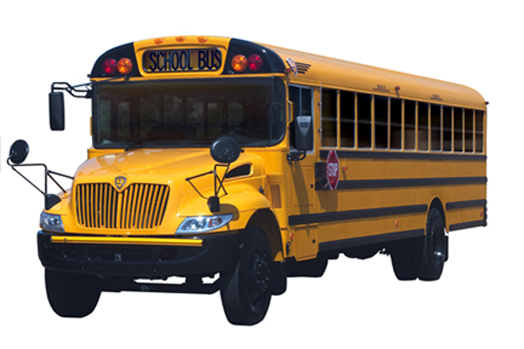 | 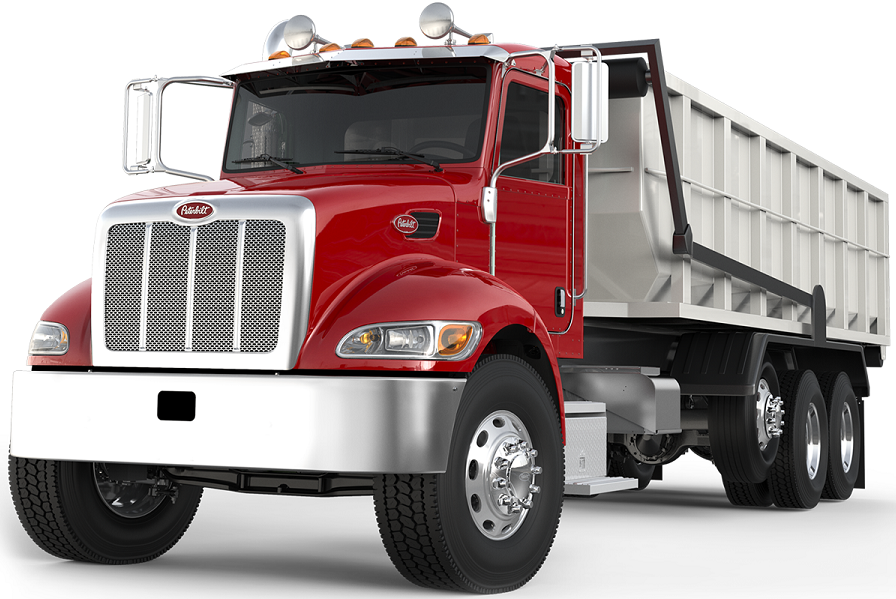 |  |  | 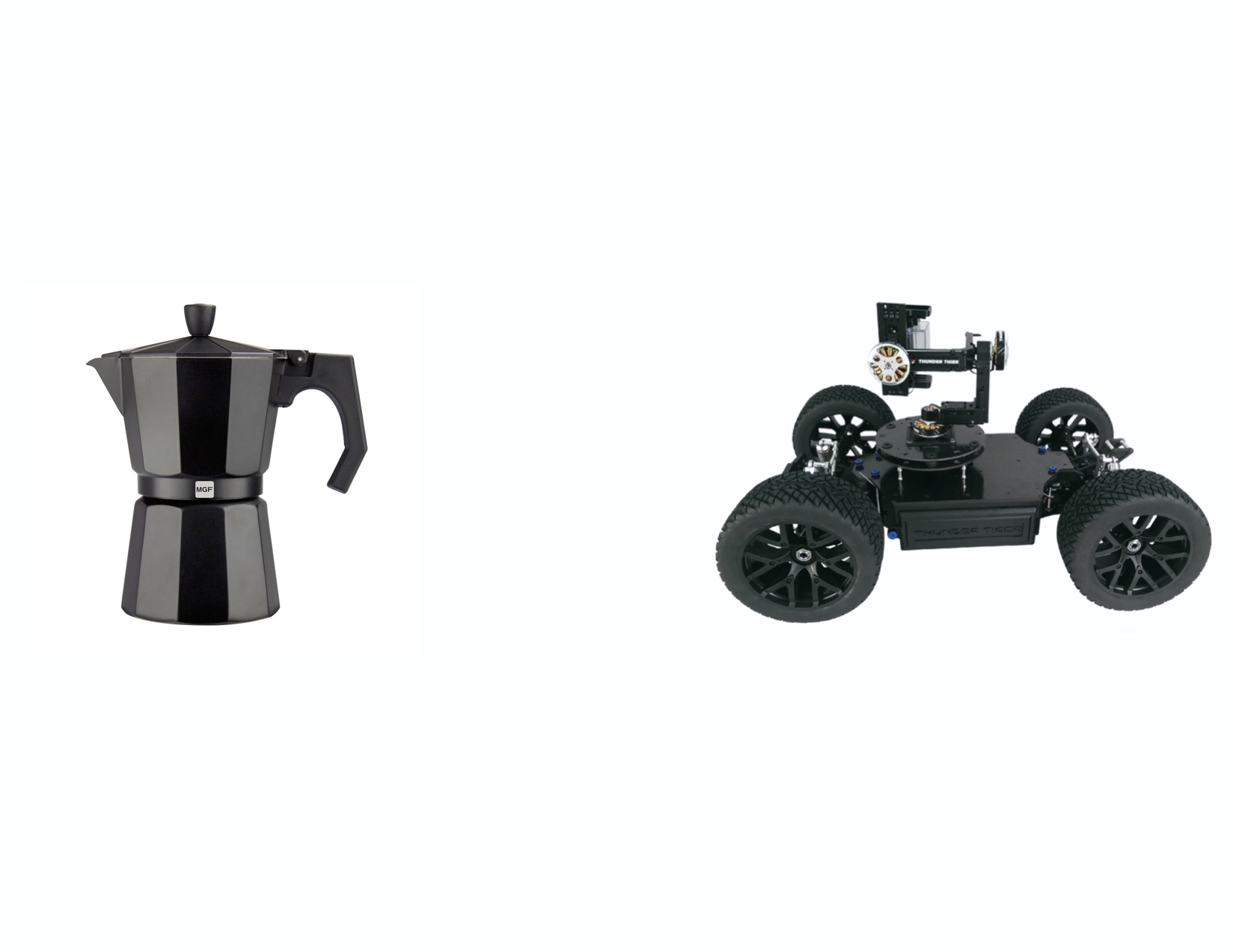 |
| Vehicles: auditory stream | Ooh! Look! A car! Do you see the car? | Ooh! Look! A bus! Do you see the bus? | Ooh! Look! A truck! Do you see the truck? | Ooh! A dax! That’s a nice dax! I like daxes! | Let’s play a game! Let’s find the dax! | Now look! Where is the *lif*? [2 s delay] Can you find the *lif*? |
| Animals: object images | 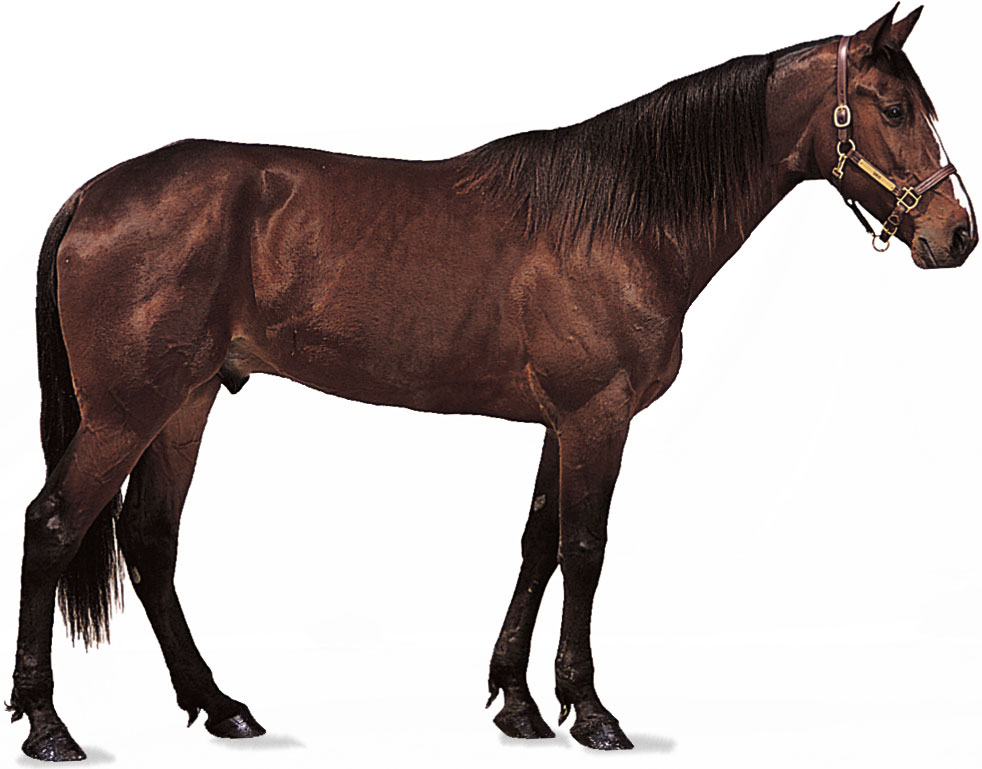 | 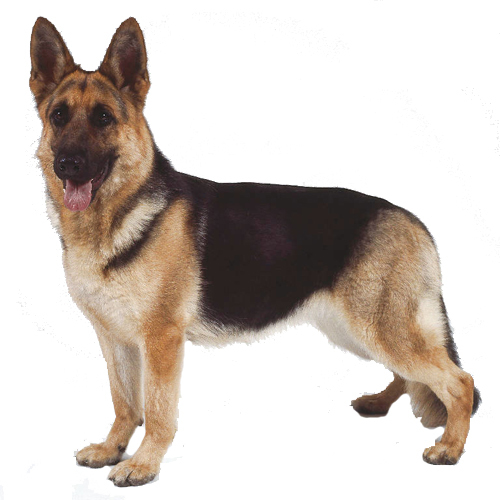 | 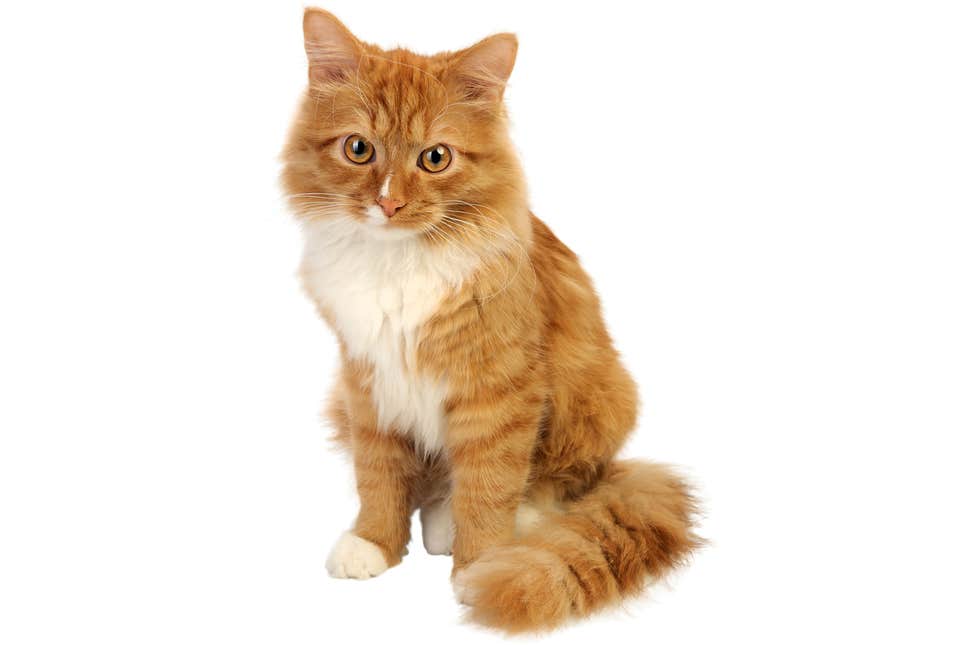 |  |  | 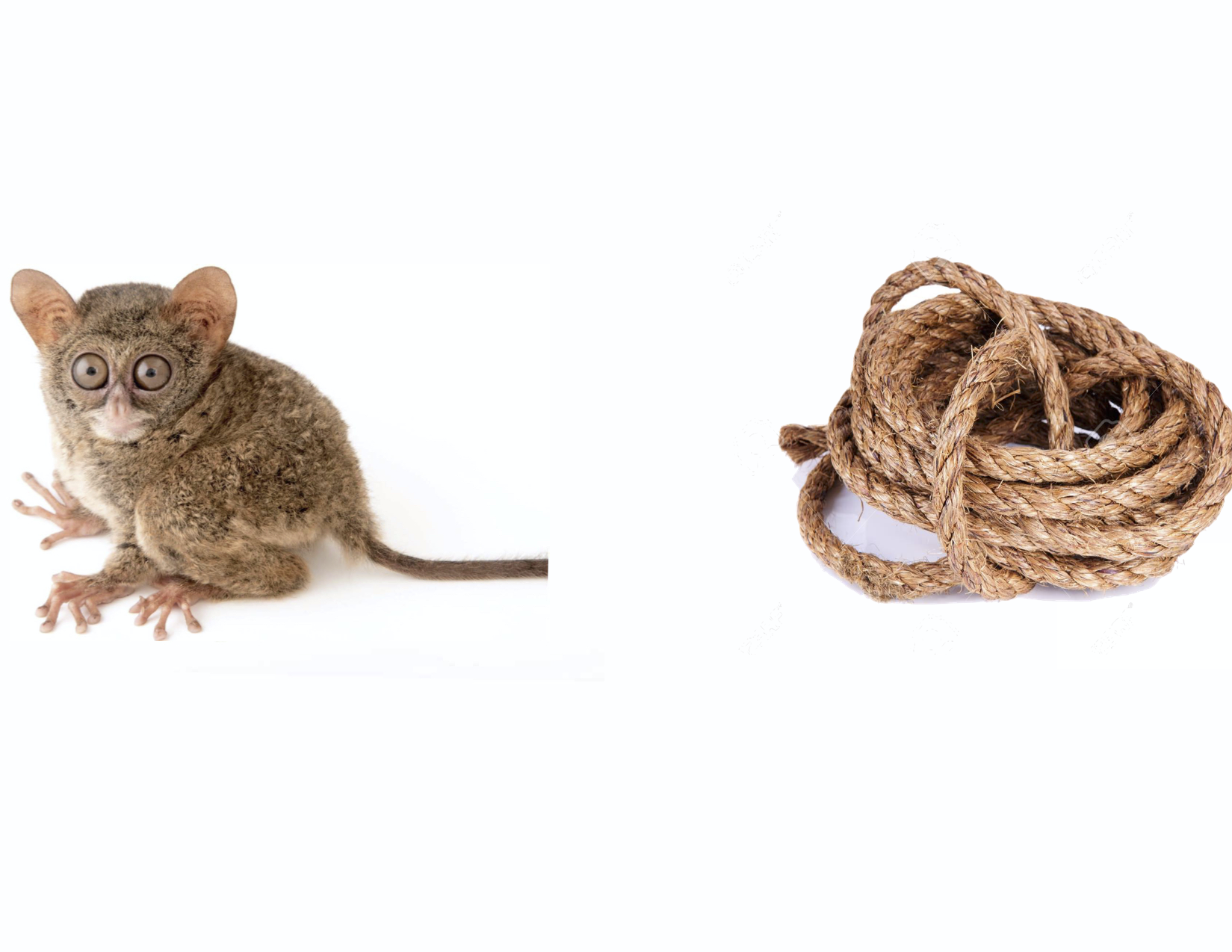 |
| Animals: auditory stream | Ooh! Look! A horse! Do you see the horse? | Ooh! Look! A dog! Do you see the dog? | Ooh! Look! A cat! Do you see the cat? | Ooh! A wug! That’s a nice wug! I like wugs! | Let’s play a game! Let’s find the wug! | Now look! Where is the *neem*? [2 s delay] Can you find the *neem*? |
| Clothing: object images | 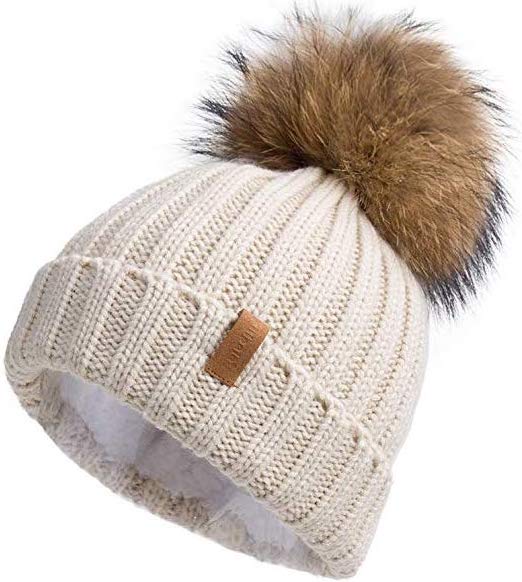 | 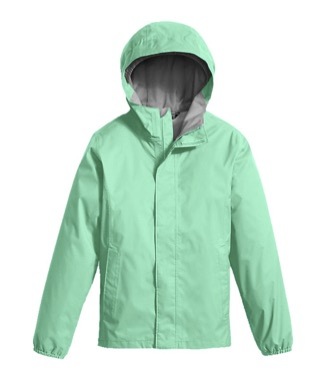 | 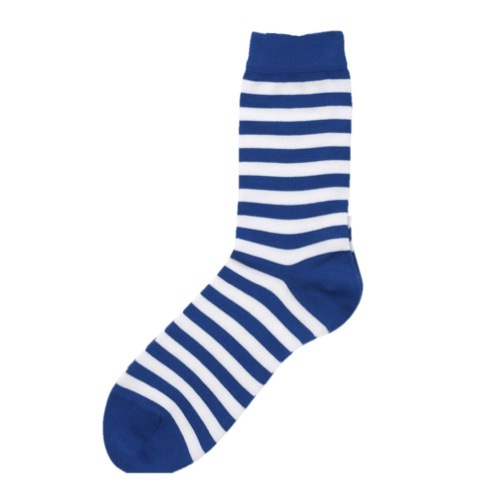 |  |  | 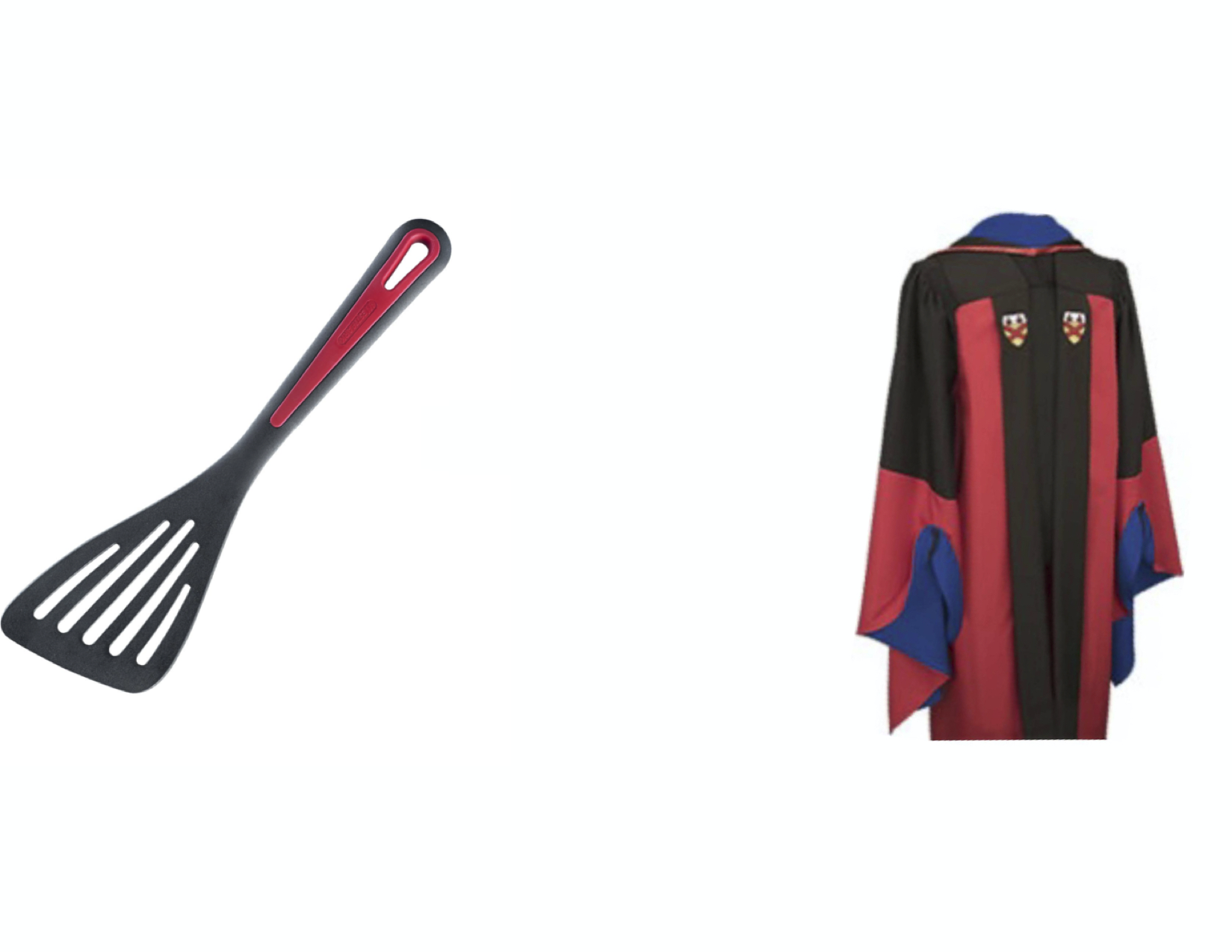 |
| Clothing: auditory stream | Ooh! Look! A hat! Do you see the hat? | Ooh! Look! A jacket! Do you see the jacket? | Ooh! Look! A sock! Do you see the sock? | Ooh! A blicket! That’s a nice blicket! I like blickets! | Let’s play a game! Let’s find the blicket! | Now look! Where is the *toma*? [2 s delay] Can you find the *toma*? |

**No Priming condition**

| Semantic neighborhood | Priming Phase | | | | | Test Phase |
| --- | --- | --- | --- | --- | --- | --- |
|  | Familiar word-object 1 | Familiar word-object 2 | Familiar word-object 3 | Novel word | |  |
| Fruits: object images | 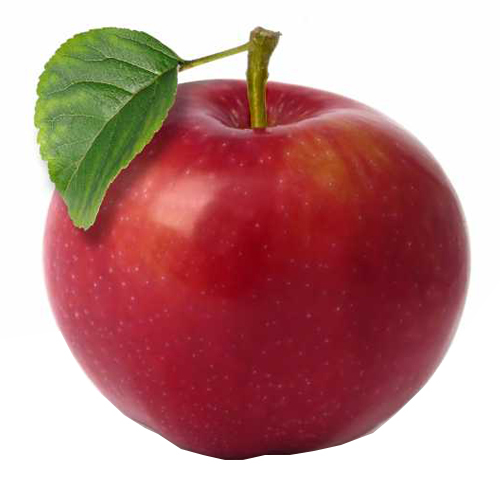 | 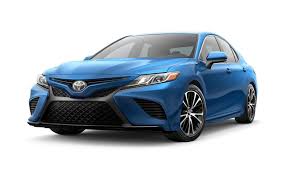 | 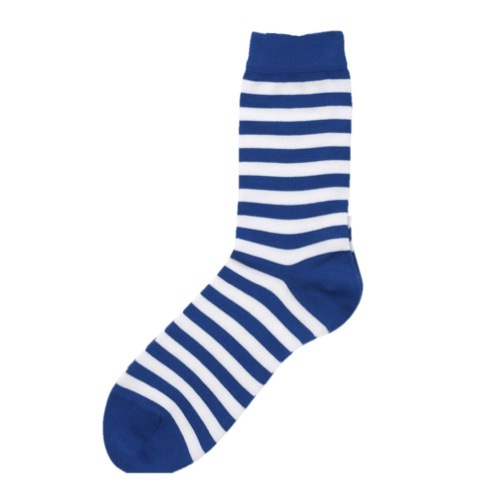 |  |  | 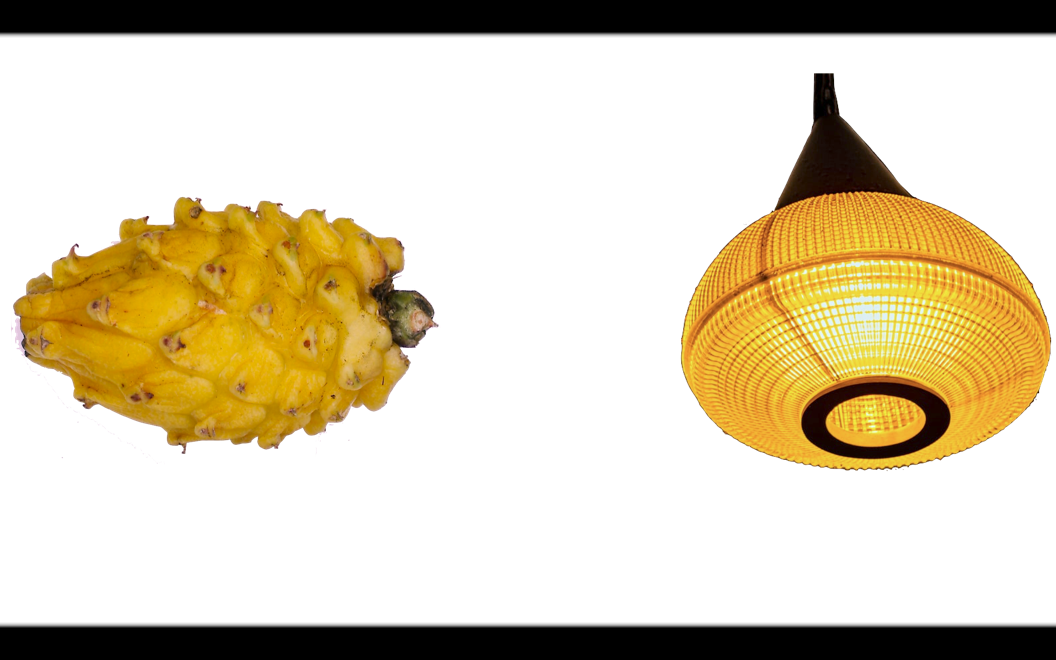 |
| Fruits: auditory stream | Ooh! Look! An apple! Do you see the apple? | Ooh! Look! A car! Do you see the car? | Ooh! Look! A sock! Do you see the sock? | Ooh! A modi! That’s a nice modi! I like modis! | Let’s play a game! Let’s find the modi! | Now look! Where is the modi? [2 s delay] Can you find the modi? |
| Vehicles: object images | 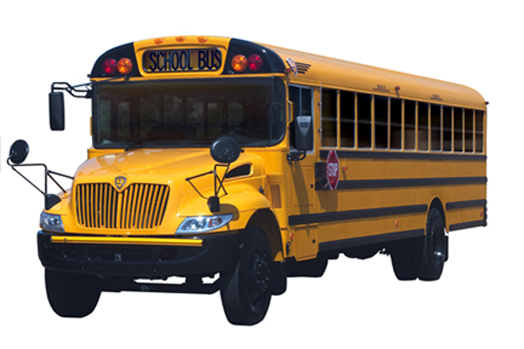 | 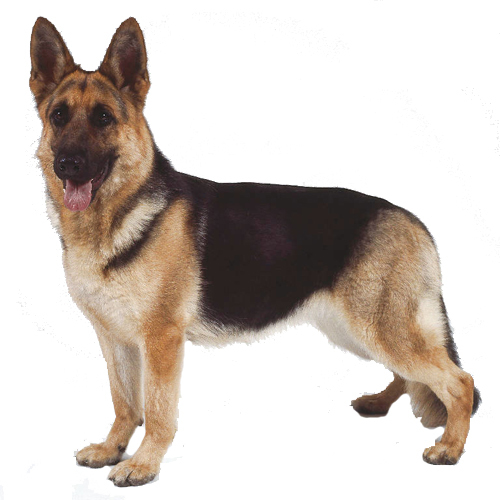 | 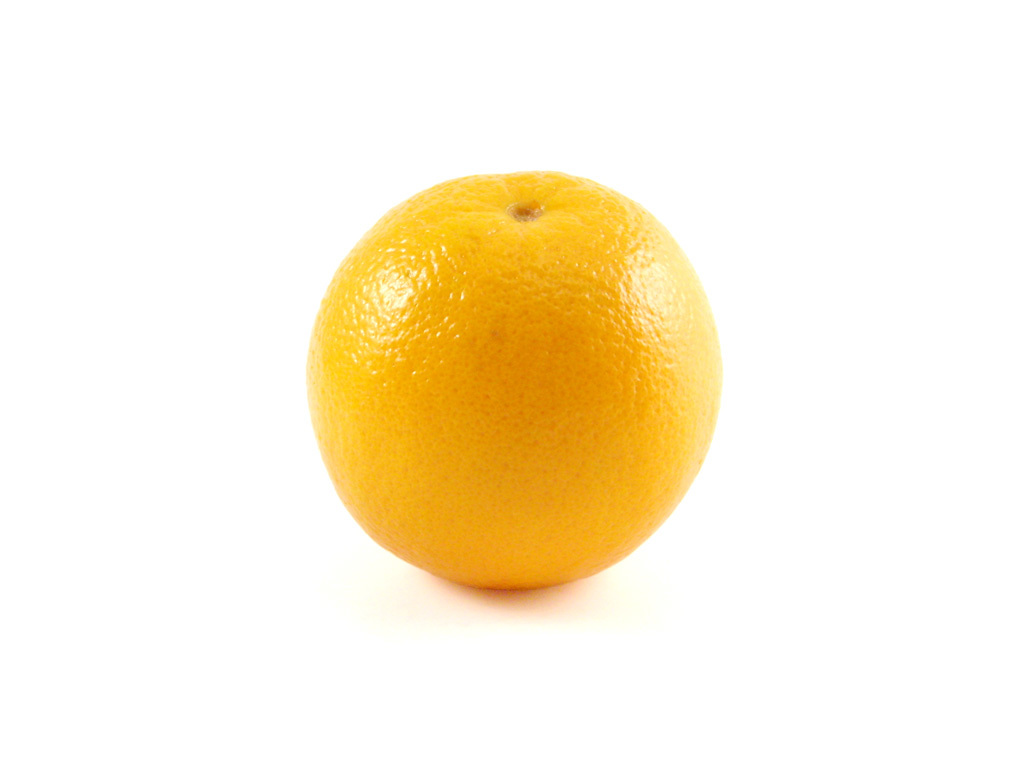 |  |  | 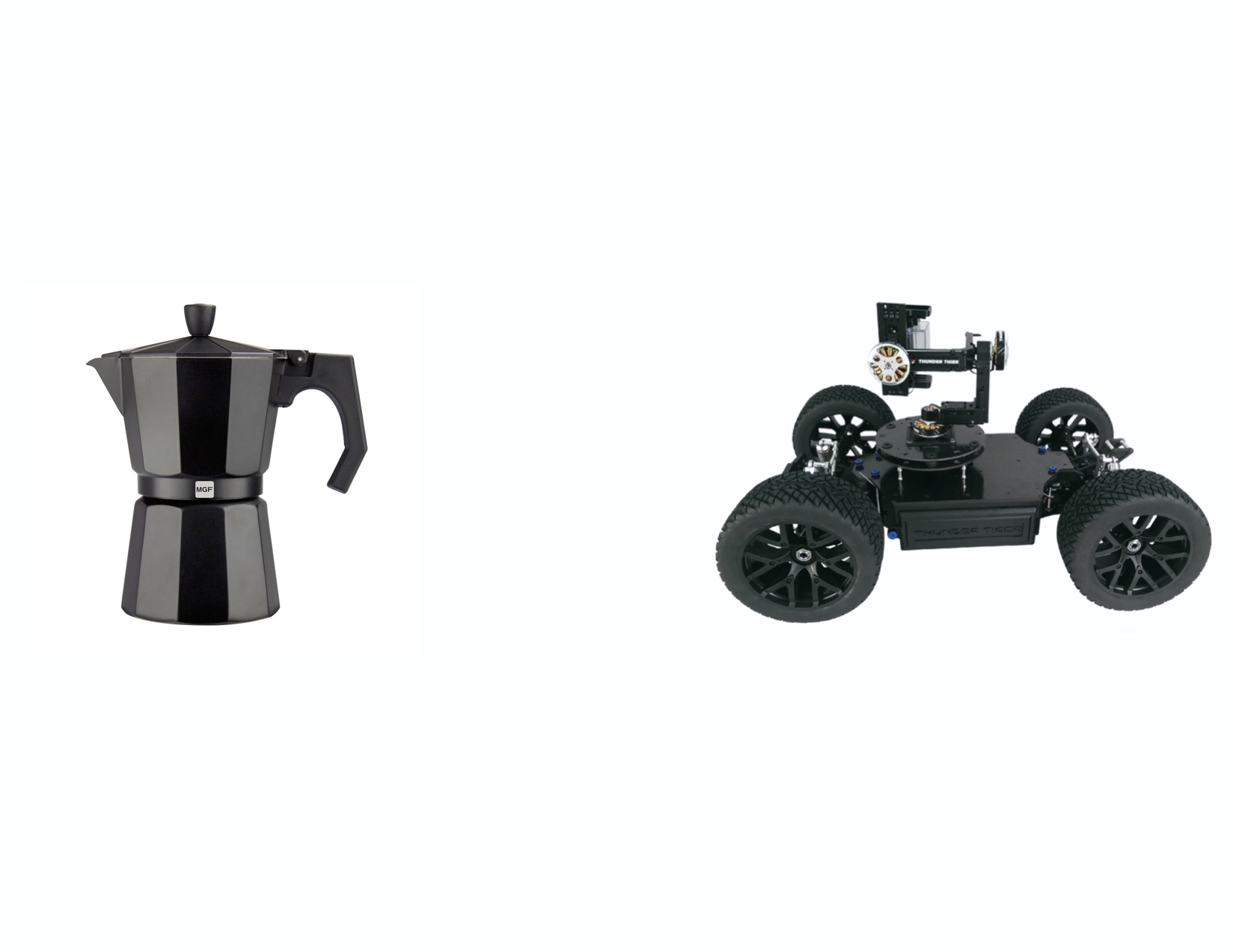 |
| Vehicles: auditory stream | Ooh! Look! A bus! Do you see the bus? | Ooh! Look! A dog! Do you see the dog? | Ooh! Look! An orange! Do you see the orange? | Ooh! A dax! That’s a nice dax! I like daxes! | Let’s play a game! Let’s find the dax! | Now look! Where is the dax? [2 s delay] Can you find the dax? |
| Animals: object images | 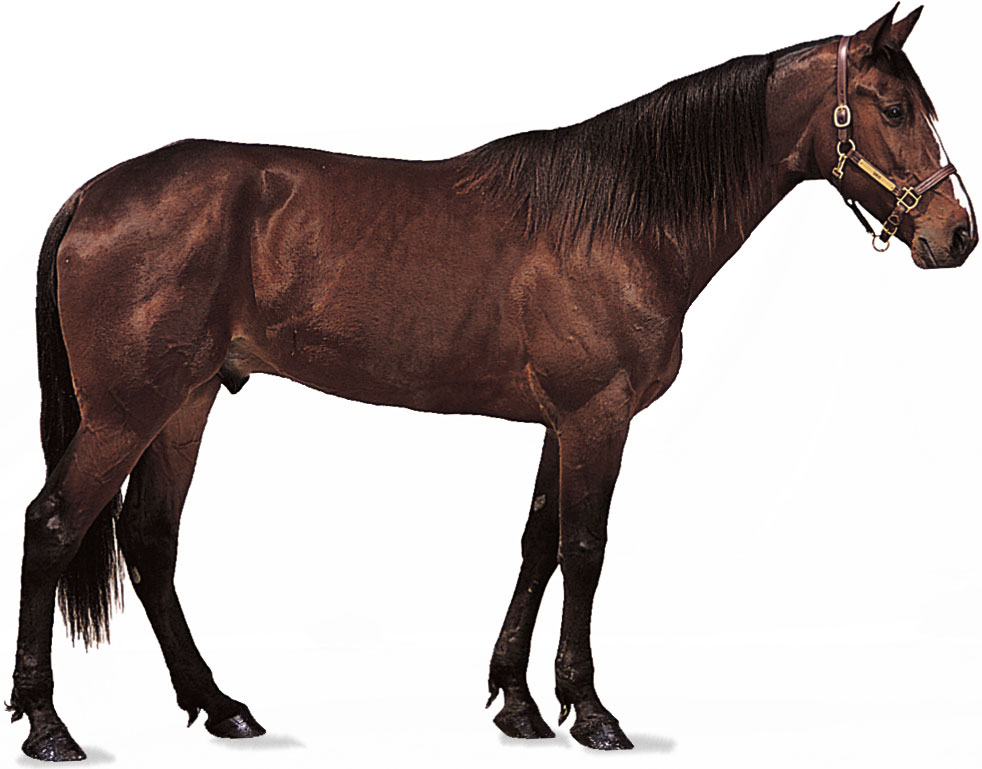 | 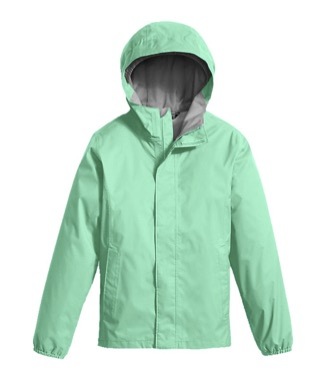 | 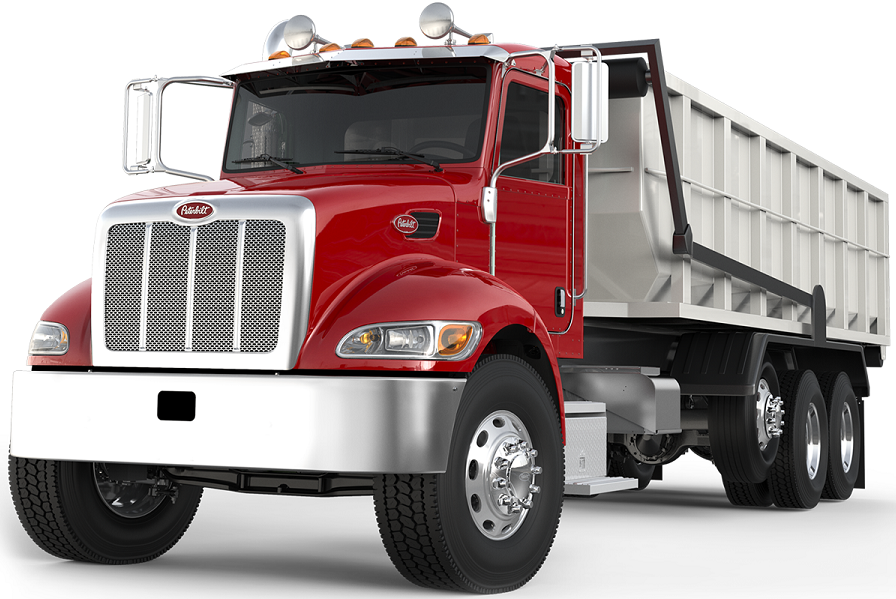 |  |  | 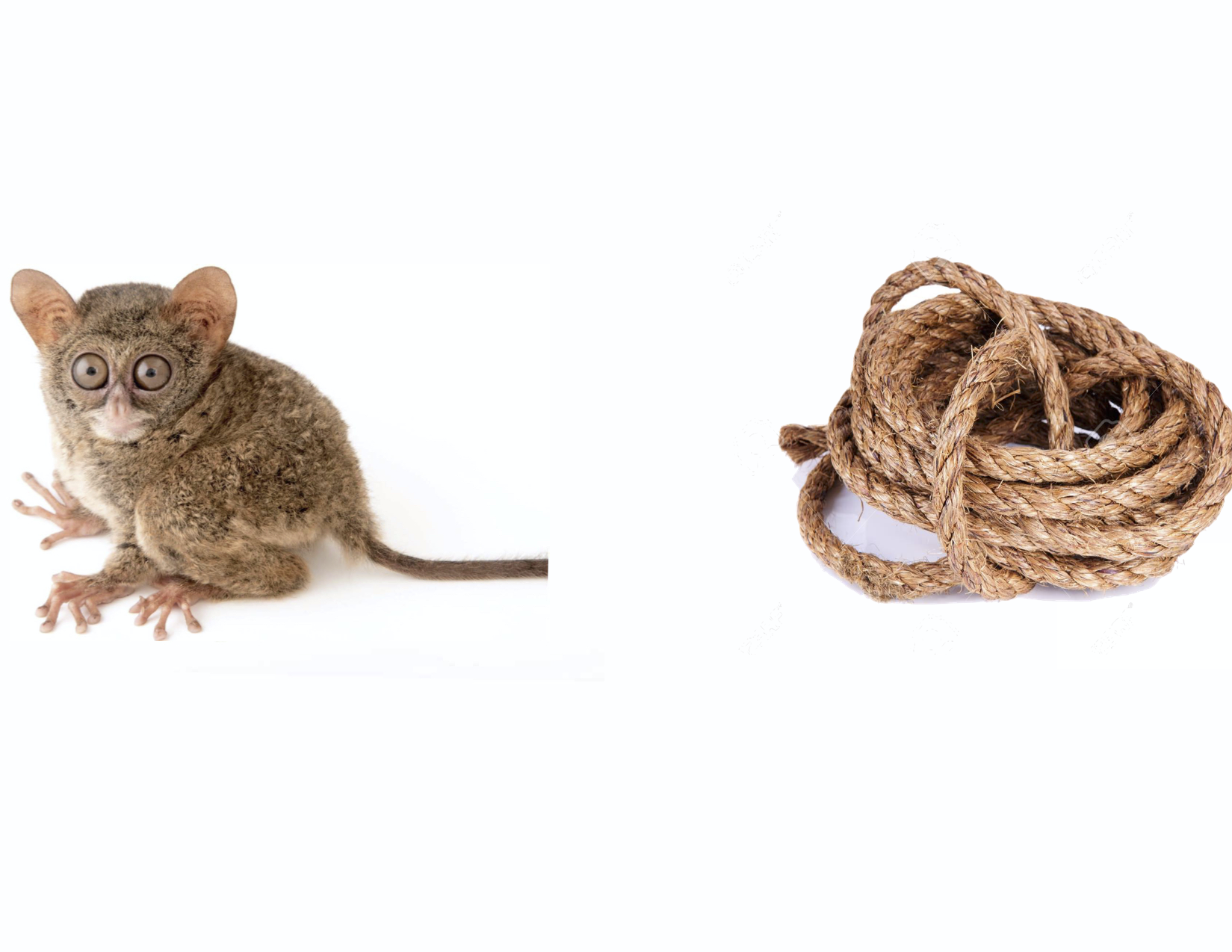 |
| Animals: auditory stream | Ooh! Look! A horse! Do you see the horse? | Ooh! Look! A jacket! Do you see the jacket? | Ooh! Look! A truck! Do you see the truck? | Ooh! A wug! That’s a nice wug! I like wugs! | Let’s play a game! Let’s find the wug! | Now look! Where is the wug? [2 s delay] Can you find the wug? |
| Clothing: object images | 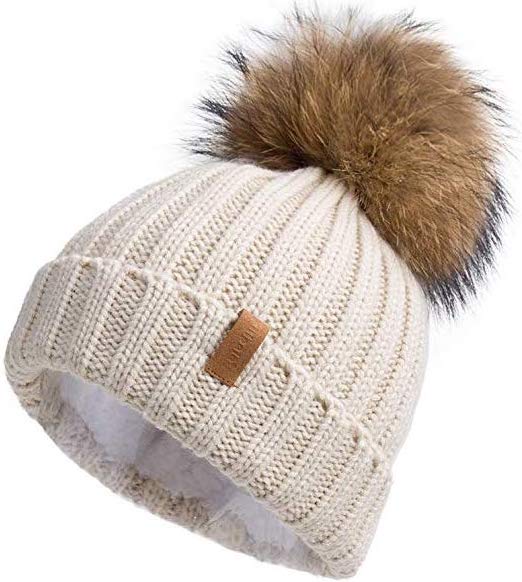 | 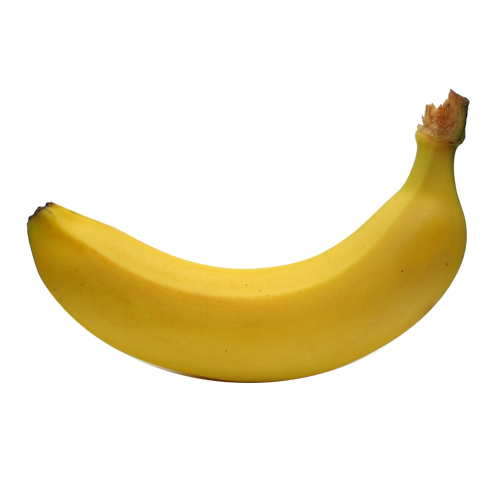 | 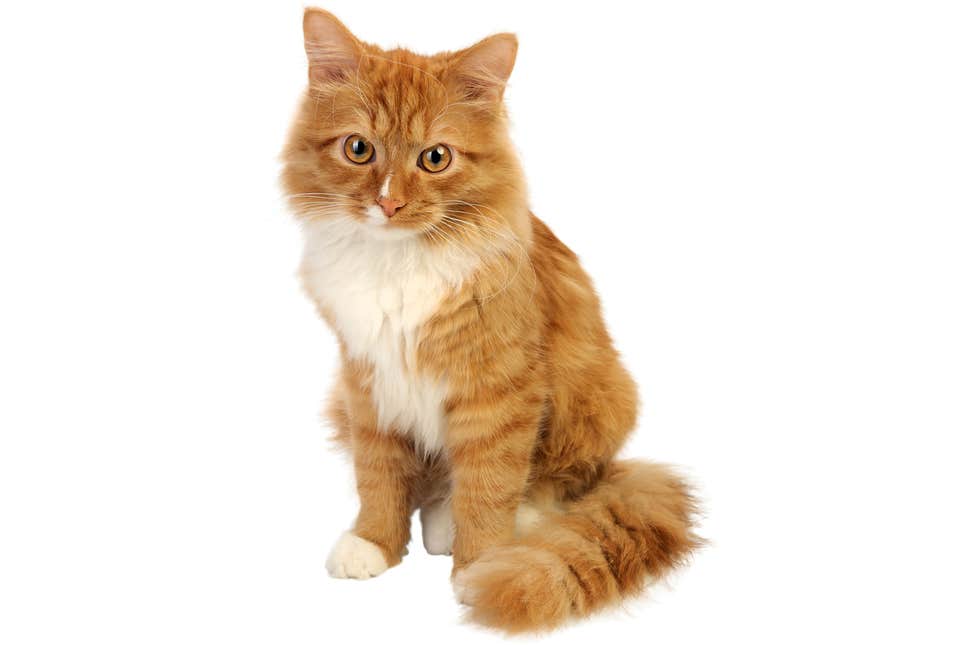 |  |  | 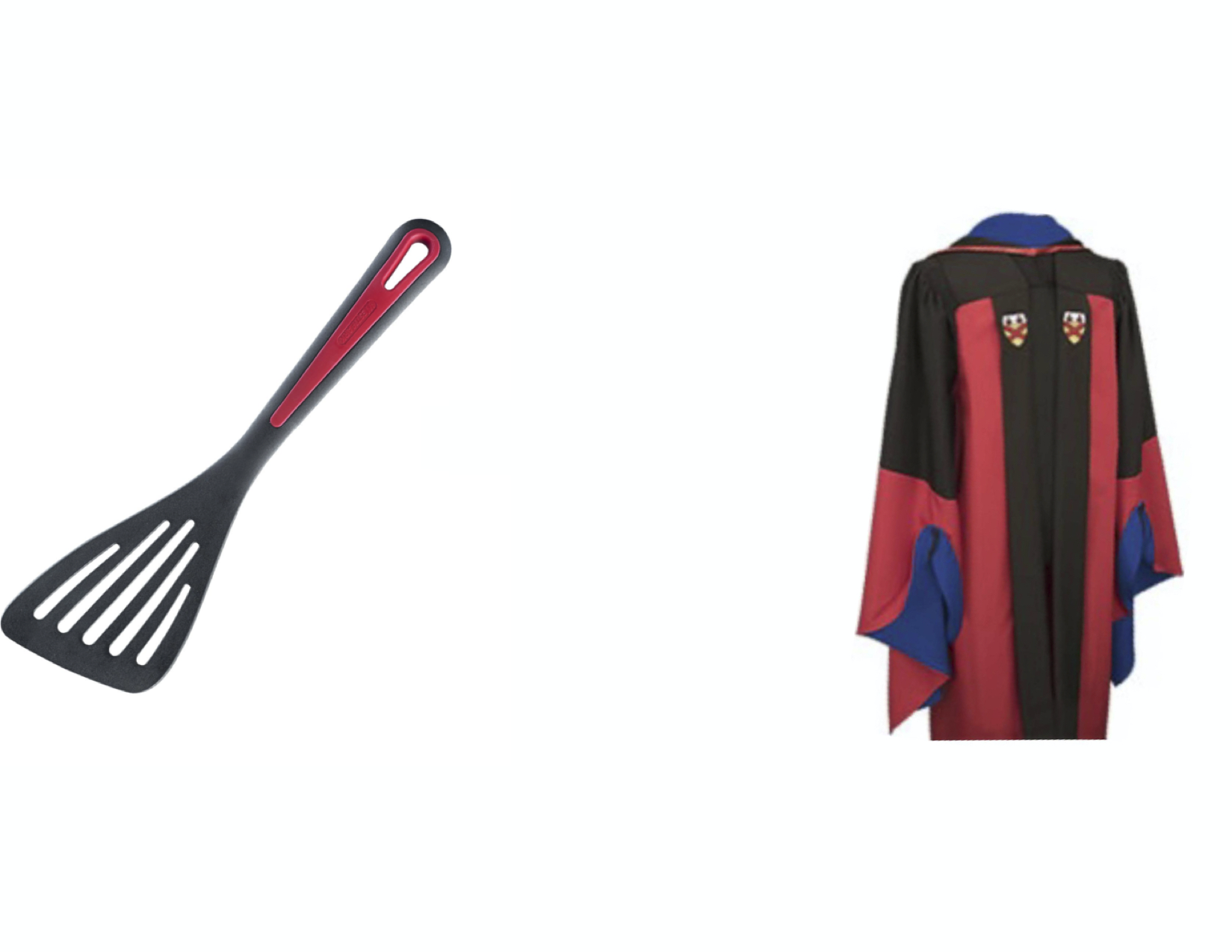 |
| Clothing: auditory stream | Ooh! Look! A hat! Do you see the hat? | Ooh! Look! A banana! Do you see the banana? | Ooh! Look! A cat! Do you see the cat? | Ooh! A blicket! That’s a nice blicket! I like blickets! | Let’s play a game! Let’s find the blicket! | Now look! Where is the blicket? [2 s delay] Can you find the blicket? |
