# Supplementary material for: Semantic priming supports infants’ ability to learn names of unseen objects
Source: PLoS One. 2025 Apr 23;20(4):e0321775. doi: 10.1371/journal.pone.0321775 (PMC12017536; doi:10.1371/journal.pone.0321775)
Supplement: S4 Appendix — (DOCX) [file pone.0321775.s004.docx]

**Appendix 4.** A comparison of looking data coded by human coders and iCatcher+

**
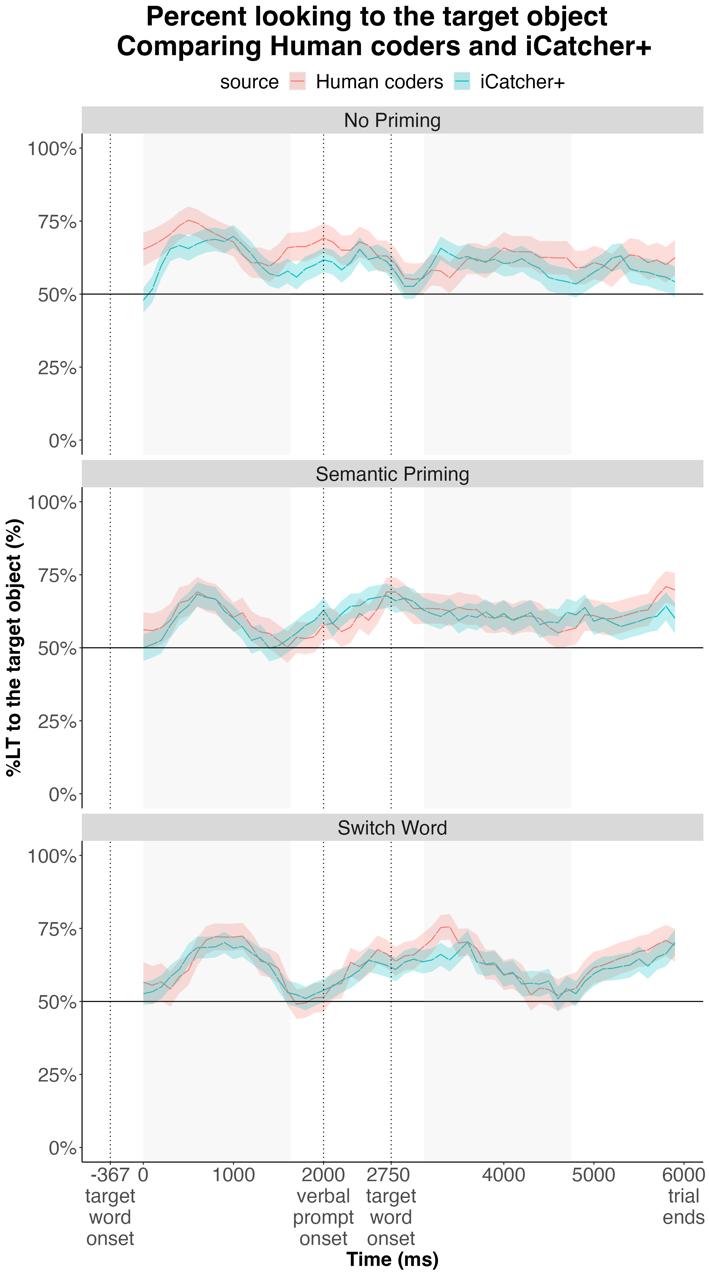
**

**
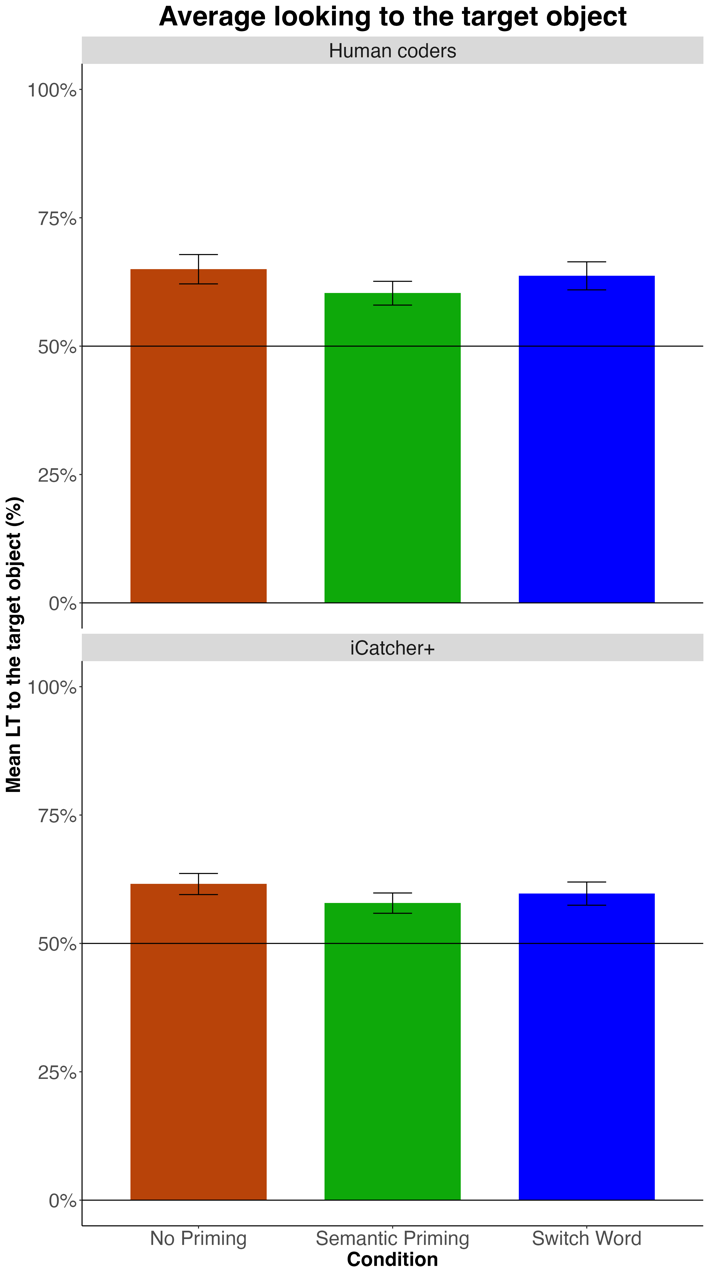
**

| **Condition** | **Mean reliability (excluding track loss)** | **Mean confidence  (excluding track loss)** | **Percent of track loss – Human coders** | **Percent of track loss – iCatcher+** |
| --- | --- | --- | --- | --- |
| No Priming | 90% | 95% | 11% | 17% |
| Semantic Priming | 87% | 94% | 12% | 21% |
| Switch Word | 87% | 94% | 14% | 23% |
