# Supplementary material for: Semantic priming supports infants’ ability to learn names of unseen objects
Source: PLoS One. 2025 Apr 23;20(4):e0321775. doi: 10.1371/journal.pone.0321775 (PMC12017536; doi:10.1371/journal.pone.0321775)
Supplement: S5 Appendix — Semantic Priming and No Priming codes look identical, except for the video recordings shown within the frame named “test trials”. A single video recording contains both Familiarization and Test. (DOCX) [file pone.0321775.s005.docx]

**Appendix 5.** A representative example of a Lookit code for Experiments 1 and 2 (Switch Word condition). Semantic Priming and No Priming codes look identical, except for the videos shown within the frame named “test trials”. A single video recording contains both Familiarization and Test.

**Experiment 1**

{

"frames": {

"webcam-display-break": {

"kind": "exp-lookit-webcam-display",

"blocks": [

{

"title": "Video check:",

"listblocks": [

{

"text": "Please turn around and hold your child over your shoulder right after pressing 'Next'. The study will begin promptly."

},

{

"text": "Please check that your child is visible."

},

{

"text": "Unless you need to pause the study (hit the 'space' bar to pause), please remain in this position until you hear 'All done'."

},

{

"text": "If the video is 'stuck' and is no longer playing, press the 'space' bar to pause the trial. Press the 'space' bar again to restart it."

}

]

}

],

"nextButtonText": "Next",

"showPreviousButton": false,

"displayFullscreenOverride": true,

"startRecordingAutomatically": false

},

"exit-survey": {

"kind": "exp-lookit-exit-survey",

"showDatabraryOptions": false,

"generateProperties": "function(expData, sequence, child, pastSessions) {var eligible = expData['1-study-procedure']['generatedProperties']['ELIGIBLE']; if (eligible) { return { 'debriefing': {'text': '<h2>What was this study about?</h2>We want to understand whether children aged 14-16 months can learn words for things that are not visible. In the Experimental Condition, on each trial, an experimenter first pointed to and named 3 familiar objects from the same category, for example, fruits. The experimenter then named a fourth object, which was not visible to the child (a modi). But because the 3 familiar objects were fruits, the child may have inferred that the last object was a fruit too. During Test, your child saw two objects that they are less likely to be familiar with—one from the same category (a dragon fruit) and one from a different category (an ottoman)–and heard a prompt, Where is the modi?. We measure where children look when they hear this prompt. If children can imagine an object that they have never seen and learn a word for it, they should look to the object from the same category (the dragon fruit). However, because children may look at the other object for unrelated reasons (prior knowledge, individual visual preferences, etc.), we average looking behavior across many children and analyze those averages. In the Control Condition, the experimenter pointed to and named 3 familiar objects from different categories. In this condition, children have no particular reason to prefer to look to one object over the other. <h2>Compensation</h2>To thank you for your participation, we will be emailing you a 5-dollar Amazon card.<ul><li>We will use the email you provided in the <b>Qualtrics survey</b>.</li><li>If you do not hear from within a week, feel free to reach out!</li><li>If you participate again with <i>another</i> child in the age range, you will receive another gift card.</li></ul>', 'title': 'Thank you for helping our research!' } }; } else { return { 'debriefing': {'text': 'Thank you for your interest in our research. At this time, we are only recruiting participants who are located in the United States. We are working to expand our reach to other countries.', 'title': 'Thank you!' } }; }}"

},

"eligibility-survey": {

"kind": "exp-lookit-survey",

"formSchema": {

"schema": {

"type": "object",

"title": "Eligibility survey",

"properties": {

"USbased": {

"enum": [

"yes",

"no"

],

"type": "string",

"title": "Are you located in the United States?",

"required": true

}

}

},

"options": {

"fields": {

"USbased": {

"type": "radio",

"message": "Please answer this question.",

"validator": "required-field"

}

}

}

},

"nextButtonText": "Continue"

},

"study-procedure": {

"kind": "exp-frame-select",

"frameOptions": [

{

"kind": "exp-frame-select",

"frameOptions": [

{

"kind": "exp-lookit-text",

"blocks": [

{

"emph": true,

"text": "You are eligible to participate in this study"

},

{

"text": "Please click on the 'Next' button."

}

]

}

]

},

{

"kind": "exp-lookit-text",

"blocks": [{

"emph": true,

"text": "We are currently unable to run participants located outside the United States."

},

{

"text": "Thank you for your interest in our research. We will contact you when we begin recruiting participants from other countries."

}

]

}

],

"generateProperties": "function(expData, sequence, child, pastSessions) {var formData = expData['0-eligibility-survey'].formData; if (formData.USbased == 'yes') { console.log('eligible'); return { 'whichFrames': 0, 'ELIGIBLE': true } } else { console.log('ineligible'); return { 'whichFrames': 1, 'ELIGIBLE': false } } }",

"selectNextFrame": "function(frames, frameIndex, frameData, expData, sequence, child, pastSessions) {var formData = expData['0-eligibility-survey'].formData; if (formData.USbased == 'yes') {return frameIndex + 1;} else {return frameIndex + 15;}}"

},

"study-intro": {

"kind": "exp-lookit-text",

"blocks": [

{

"emph": true,

"text": "Important: your child does not need to be with you until the videos begin. First, let's go over what will happen!",

"title": "Overview of the 'Using words to learn more words!' study"

},

{

"text": "During this study, your baby will watch videos of an experimenter showing and naming various objects while we record where he or she chooses to look."

},

{

"text": "You’ll have a chance to preview the videos ahead of time. After reading the instructions you’ll start the experiment when you and your baby are ready."

},

{

"text": "The video section will take about 3 minutes. Before the video, we ask you to fill out a survey. This will take 5 minutes."

},

{

"text": "After the videos, you will answer a few final questions. Then you're all done!"

}

],

"showPreviousButton": false

},

"qualtrics": {

"kind": "exp-lookit-text",

"blocks": [

{

"emph": true,

"text": "<iframe width='800' height='600' src='https://northwestern.az1.qualtrics.com/jfe/form/SV_a2Ai4ClETwc5yW9'></iframe>\nPlease remember to hit 'Submit'.\nIf the survey does not show up in the window above, please click <u><a href='https://northwestern.az1.qualtrics.com/jfe/form/SV_a2Ai4ClETwc5yW9' target='_blank' rel='noopener'>here</a></u> and return to this tab after completing the survey.",

"title": "Before we begin, please fill out this survey."

}

],

"showPreviousButton": false

},

"calibration": {

"kind": "exp-lookit-calibration",

"baseDir": "https://www.mit.edu/~kimscott/placeholderstimuli/",

"dorecording": true,

"showWaitForUploadMessage": false,

"waitForUploadImage": [],

"audioTypes": [

"ogg",

"mp3"

],

"videoTypes": [

"webm",

"mp4"

],

"calibrationLength": 2000,

"calibrationAudio":"chimes",

"calibrationVideo": "attentiongrabber",

"calibrationPositions": [

"left",

"right",

"left",

"right",

"center"

]

},

"test-trials": {

"kind": "choice",

"baseDir": "https://www.mit.edu/~kimscott/placeholderstimuli/",

"sampler": "random-parameter-set",

"frameList": [{

"video": {

"loop": false,

"position": "fill",

"source": "VIDEO1"

},

"doRecording": true,

"requireVideoCount": 1,

"requireAudioCount": 0

},

{

"video": {

"loop": false,

"position": "fill",

"source": "VIDEO2"

},

"doRecording": true,

"requireVideoCount": 1,

"requireAudioCount": 0

},

{

"video": {

"loop": false,

"position": "fill",

"source": "VIDEO3"

},

"doRecording": true,

"requireVideoCount": 1,

"requireAudioCount": 0

},

{

"video": {

"loop": false,

"position": "fill",

"source": "VIDEO4"

},

"doRecording": true,

"requireVideoCount": 1,

"requireAudioCount": 0

},

{

"video": {

"loop": true,

"top": 40,

"left": 45,

"width": 10,

"source": "attentiongrabber"

},

"audio": {

"loop": false,

"source": "all_done_HO"

},

"doRecording": false,

"requireAudioCount": 1,

"requireVideoCount": 0

}],

"parameterSets": [

{

"VIDEO1": [{"src": "https://fragmean.s3.us-east-2.amazonaws.com/Follow-up+control+fruits.mp4",

"type": "video/mp4"}],

"VIDEO2": [{"src": "https://fragmean.s3.us-east-2.amazonaws.com/Follow-up+control+vehicles.mp4",

"type": "video/mp4"}],

"VIDEO3": [{"src": "https://fragmean.s3.us-east-2.amazonaws.com/Follow-up+control+animals.mp4",

"type": "video/mp4"}],

"VIDEO4": [{"src": "https://fragmean.s3.us-east-2.amazonaws.com/Follow-up+control+clothing.mp4",

"type": "video/mp4"}]

},

{

"VIDEO2": [{"src": "https://fragmean.s3.us-east-2.amazonaws.com/Follow-up+control+fruits.mp4",

"type": "video/mp4"}],

"VIDEO3": [{"src": "https://fragmean.s3.us-east-2.amazonaws.com/Follow-up+control+vehicles.mp4",

"type": "video/mp4"}],

"VIDEO4": [{"src": "https://fragmean.s3.us-east-2.amazonaws.com/Follow-up+control+animals.mp4",

"type": "video/mp4"}],

"VIDEO1": [{"src": "https://fragmean.s3.us-east-2.amazonaws.com/Follow-up+control+clothing.mp4",

"type": "video/mp4"}]

},

{

"VIDEO3": [{"src": "https://fragmean.s3.us-east-2.amazonaws.com/Follow-up+control+fruits.mp4",

"type": "video/mp4"}],

"VIDEO4": [{"src": "https://fragmean.s3.us-east-2.amazonaws.com/Follow-up+control+vehicles.mp4",

"type": "video/mp4"}],

"VIDEO1": [{"src": "https://fragmean.s3.us-east-2.amazonaws.com/Follow-up+control+animals.mp4",

"type": "video/mp4"}],

"VIDEO2": [{"src": "https://fragmean.s3.us-east-2.amazonaws.com/Follow-up+control+clothing.mp4",

"type": "video/mp4"}]

},

{

"VIDEO4": [{"src": "https://fragmean.s3.us-east-2.amazonaws.com/Follow-up+control+fruits.mp4",

"type": "video/mp4"}],

"VIDEO1": [{"src": "https://fragmean.s3.us-east-2.amazonaws.com/Follow-up+control+vehicles.mp4",

"type": "video/mp4"}],

"VIDEO2": [{"src": "https://fragmean.s3.us-east-2.amazonaws.com/Follow-up+control+animals.mp4",

"type": "video/mp4"}],

"VIDEO3": [{"src": "https://fragmean.s3.us-east-2.amazonaws.com/Follow-up+control+clothing.mp4",

"type": "video/mp4"}]

}

],

"commonFrameProperties": {

"kind": "exp-lookit-video",

"baseDir": "https://www.mit.edu/~kimscott/intermodal/",

"audioTypes": ["ogg",

"mp3"],

"videoTypes": ["webm",

"mp4"],

"backgroundColor": "white",

"autoProceed": true,

"pauseAudio": "pause_HO",

"pauseVideo": "attentiongrabber",

"unpauseAudio": "return_after_pause_HO"

}

},

"instructions": {

"kind": "exp-lookit-instructions",

"blocks": [

{

"title": "Instructions",

"text": "The video section will take about 3 minutes to complete. After that, you will be able to select the level of privacy for your data."

},

{

"title": "Study overview",

"listblocks": [

{

"text": "To get your baby's attention, first they will see a moving shape and hear a chime. "

},

{

"text": "Then your baby will watch four videos, each about 45 seconds long."

}

]

},

{

"title": "During the videos",

"listblocks": [

{

"text": "Please face away from the screen, holding your infant so they can look over your shoulder. Please don't look at the videos yourself--we may not be able to use your infant’s data in that case.",

"image": {

"alt": "Father holding child looking over his shoulder",

"src": "https://s3.amazonaws.com/lookitcontents/exp-physics/OverShoulder.jpg"

}

},

{

"text": "This is because your child is learning from you all the time and may pick up on even very small cues about what you think. But if you can't see the stimuli, we're definitely only measuring your child's own beliefs."

},

{

"text": "Don’t worry if your baby isn’t looking at the screen the entire time! Please just try to keep them facing the screen so they can look if they want to."

}

]

},

{

"title": "Pausing and stopping",

"listblocks": [

{

"text": "If your child gets fussy or distracted, or you need to attend to something else for a moment, you can pause the study by pressing the space bar."

},

{

"text": "If you need to end the study early, try closing the window or tab and you should see an 'exit' option pop up. You’ll be prompted to note any technical problems you might be experiencing and to select a privacy level for your videos."

}

]

},

{

"text": "Please turn the volume up so it's easy to hear but still comfortable.",

"title": "Test your audio",

"mediaBlock": {

"text": "You should hear 'Ready... go?'",

"isVideo": false,

"sources": [

{

"src": "https://fragmean.s3.us-east-2.amazonaws.com/Ready.mp3",

"type": "audio/mp3"

},

{

"src": "OGG_SOURCE_HERE",

"type": "audio/ogg"

}

],

"mustPlay": true,

"warningText": "Please try playing the sample audio."

}

}

],

"nextButtonText": "Next"

},

"video-config": {

"kind": "exp-video-config",

"troubleshootingIntro": "Please follow the links below for additional help and troubleshooting."

},

"video-consent": {

"kind": "exp-lookit-video-consent",

"template": "consent_005",

"PIName": "Dr. Sandra Waxman",

"PIContact": "Elena Luchkina at elena.luchkina@northwestern.edu",

"institution": "Northwestern University",

"omit_injury_phrase": true,

"summary_statement": "\n\n<b>Title of Research Study:<i> Developmental origins and downstream consequences of verbal reference</i></b>\n\n<b>IRB Study Number:</b> STU00214867\n<b>Principal Investigator:</b> Sandra Waxman\n<b>Supported By:</b> This research is supported by the Psychology Department at Weinberg College of Arts and Sciences at Northwestern University and the National Institutes of Health (NIH).\n<b>Financial Interest Disclosure:</b> There is no conflict of interest.\n<h2>Key Information about this research study:</h2>This is a short summary of this study to help you decide whether to permit your child to be a part of this study:\n<ul><li>The purpose of this study is to better understand the link between language and thought in infancy and childhood. We will measure babies’ looking direction to understand what they learned about the new words. </li><li>Your child will be asked to watch a short video or slide show. You will be asked to complete a survey about your child’s vocabulary.</li><li>This study takes about 15 minutes to complete. After the study, you may be contacted by email two times over the course of a year to complete the vocabulary survey again.</li><li>Data is stored securely on Lookit servers and by researchers at Northwestern. However, there is always a small risk that data transmitted over the internet may be intercepted or that the security of stored data may be compromised.</li><li>During the study, you and your child will be audio- and videotaped.</li></ul><b><i>Please note: If you or your child do not wish to be recorded, it is not possible for your child to be in this study.</i></b>\n<ul><li>The vocabulary survey is completed via a Qualtrics form hosted by the Infant and Child Development Center at Northwestern University. Qualtrics is an online survey platform accessed via a secure connection. Terms of service, addressing confidentiality, may be viewed at https://www.qualtrics.com/support/survey-platform/getting-started/data-protection-privacy/.</li><li>Please refer to the <a href='https://lookit.mit.edu/termsofuse/' target='_blank'>Lookit Terms of Use</a> (https://lookit.mit.edu/termsofuse/) for more detail on our use of the Lookit platform.</li><li>To review Lookit’s Privacy Statement again, please use this link <a href='https://lookit.mit.edu/privacy/' target='_blank'>Privacy Statement</a> (https://lookit.mit.edu/privacy/).</li></ul><b><i>Please note: If you do not consent to share your and your child’s data on the Lookit server, it is not possible for your child to be in this study.</i></b>\n<h2>Why am I being asked to take part in this research study?</h2>We are asking you to take part in this research study because your child is of the appropriate age and meets language and health eligibility criteria.\n<h2>How many people will be in this study?</h2>We expect about 80 people will be in this research study.",

"purpose":"<ul><li>Whether or not you take part is up to you.</li><li>You can choose not to take part.</li><li>You can agree to take part and later change your mind.</li><li>Your decision will not be held against you.</li></ul>",

"procedures":"Your child will watch a short video or slide show which will focus on either (1) communicative (e.g., language) or non-communicative (e.g., music) sounds, or (2) moving objects and characters. You will be asked to complete a survey about your child’s vocabulary. After the study you may be contacted by email two times over the course of the year to complete the vocabulary survey again.",

"risk_statement": "Data is stored securely on Lookit servers and by researchers at Northwestern. However, there is always a small risk that data transmitted over the internet may be intercepted or that the security of stored data may be compromised.",

"include_databrary": false,

"voluntary_participation": "You can decide not to participate in this research, or you can start the study and then decide to leave the study at any point and it will not be held against you. To do so, simply close the browser window. Any data already collected will not be saved.",

"datause": "<ul><li>Upon receiving results of your survey, any possible identifiers will be deleted.</li><li>You will be identified only by a unique subject number.</li><li>Your email address will be stored separately from your survey data</li><li>All information will be kept on a secure server only accessible by the research team.</li><li>The results of the research study may be published, but your name will not be used.</li></ul>Please refer to the <a href='https://lookit.mit.edu/termsofuse/' target='_blank'>Lookit Terms of Use</a> for more detail on our use of the Lookit platform.\n<h2>Certificate of Confidentiality</h2> This research is covered by a Certificate of Confidentiality from the National Institutes of Health. This means that the researchers cannot release or use information, documents, or samples that may identify you in any action or suit unless you say it is okay. They also cannot provide them as evidence unless you have agreed. This protection includes federal, state, or local civil, criminal, administrative, legislative, or other proceedings. An example would be a court subpoena.\n\nIdentifiable information that could still be disclosed beyond the research team: The Certificate does not stop reporting that federal, state or local laws require. Some examples are laws that require reporting of child or elder abuse, some communicable diseases, and threats to harm yourself or others. The Certificate cannot be used to stop a sponsoring United States federal or state government agency from checking records or evaluating programs. The Certificate does not stop disclosures required by the federal Food and Drug Administration (FDA). The Certificate also does not prevent your information from being used for other research if allowed by federal regulations.\n\nResearchers may release information about you when you say it is okay. For example, you may give them permission to release information to insurers, medical providers or any other persons not connected with the research. The Certificate of Confidentiality does not stop you from willingly releasing information about your involvement in this research. It also does not prevent you from having access to your own information.",

"gdpr": false,

"research_rights_statement":"This research has been reviewed and approved by an Institutional Review Board (“IRB”). You may talk to them at (312) 503-9338 or <a href = 'mailto: irb@northwestern.edu'>irb@northwestern.edu</a> if:<ul><li>Your questions, concerns, or complaints are not being answered by the research team</li><li>You cannot reach the research team</li><li>You want to talk to someone besides the research team</li><li>You have questions about your rights as a research participant</li><li>You want to get information or provide input about this research.</li></ul>",

"payment": "If you agree to take part in this research study, after you finish the study and if you meet these eligibility criteria, we will email you a $5 Amazon gift card within 1 week. We will send the card even if you do not finish the whole study or we are not able to use your child’s data.\n\nTo be eligible for compensation, the following criteria must be met:<ul><li>Your child must be in the age range for this study;</li><li>You need to submit a valid video consent statement;</li><li>We need to see that there is a child with you in the study participation video.</li></ul>",

"benefits_header": "What else do I need to know?",

"purpose_header":"What should I know about a research study?",

"procedures_header":"If you say that “Yes, you want to be in this research,” here is what you will do:",

"risk_header": "Is there any way being in this study could be bad for me?",

"additional_segments": [

{

"text": "Efforts will be made to limit the use and disclosure of your personal information, including research study records, to people who have a need to review this information.",

"title": "What happens to the information collected for the research?"

},

{

"text": "If you want a copy of this consent for your records, press “download” and save the document to your computer.",

"title": "Saving a copy of the consent form"

}

]

},

"video-preview": {

"kind": "exp-lookit-stimuli-preview",

"blocks": [

{

"title": "Video preview",

"text": "If you'd like to see the videos your child will be shown, you can take a look ahead of time now. It's important that you preview the videos without your child, so that the videos will still be new to them.\n\nIf you choose to skip, press 'Skip preview'."

}

],

"stimuli": [

{

"video": [

{

"src": "https://fragmean.s3.us-east-2.amazonaws.com/Miriam_animals_final_short.mp4",

"type": "video/mp4"

}

],

"caption": "For each trial, there will be a woman on the screen pointing at images of objects. Here's an example.\n\nAs soon as you press 'Continue', please turn around and hold your baby over your shoulder."

}

],

"videoTypes": [

"webm",

"mp4"

],

"baseDir/ext": "https://fragmean.s3.us-east-2.amazonaws.com/",

"skipButtonText": "Skip preview",

"previewButtonText": "I'd like to preview the videos",

"showPreviousButton": true

}

},

"sequence": [

"eligibility-survey",

"study-procedure",

"study-intro",

"video-config",

"video-consent",

"qualtrics",

"instructions",

"video-preview",

"webcam-display-break",

"calibration",

"test-trials",

"exit-survey"

]

}

**Experiment 2**

function generateProtocol(child, pastSessions) {

// -------- Helper functions ----------------------------------------------

function shuffle(array) {

var shuffled = Ember.$.extend(true, [], array); // deep copy array

for (var i = array.length - 1; i > 0; i--) {

var j = Math.floor(Math.random() * (i + 1));

var temp = shuffled[i];

shuffled[i] = shuffled[j];

shuffled[j] = temp;

}

return shuffled;

}

// Returns a random element of an array, and removes that element from the array

function pop_random(array) {

if (array.length) {

var randIndex = Math.floor(Math.random() * array.length);

return array.splice(randIndex, 1)[0];

}

return null;

}

// -------- End helper functions -------------------------------------------

var count = 0;

for (var i = 0; i < pastSessions.length; i++) {

if (pastSessions[i].get('completed') === true) {

count = count + 1

}

}

var name = child.get('id');

var survey_text = name.toString();

var COUNT_1 = count - 1;

var _LEFT = 7 - COUNT_1

var EXIT_COUNT = count

var EXIT_LEFT = 7 - EXIT_COUNT

var welcome_text = 'You have completed ' + COUNT_1.toString() + ' of 7 reading sessions. You have ' + _LEFT.toString() + ' sessions to go.\nClick "Next" to start a new session.'

var exit_fam_only = 'You have completed ' + EXIT_COUNT.toString() + ' of 7 reading sessions. You have ' + EXIT_LEFT.toString() + ' sessions to go. When you have completed all 7 sessions, we will email you a $5 Amazon gift card within a week.'

var exit_first = 'You have successfully signed up to participate in our longitudinal study "Picture book for better word learning". In this study we are investigating whether babies can learn the names of hidden things (which they had never seen before) after their knowledge of familiar object names is strengthened by picture book reading.\n\n <b>Next sessions</b>\nWhen you log into the study again, you will be asked to complete 7 digital book reading sessions, each on a different day. Your reading sessions will be recorded. Please do not record more than one reading session per day. Only one session will be counted towards the total. Please read each page at least once in a given session. After you complete all 7 sessions, the next time you log into the study, you will be prompted to complete the final experiment. \n\n<b>Compensation</b>\nTo thank you for your participation, we will be emailing you Amazon gift cards.<ul><li>We will use the email you provided in the <b>Qualtrics survey</b>.</li><li>You will be paid $5 after completing 7 digital book reading sessions and an additional $10 after completing the final part of the study.</li><li>If you participate again with <i>another</i> baby in the age range, you will receive another set of gift cards ($5 and $10).</li></ul>'

var exit_last = "<b>What was this study about?</b>\nWe want to understand if babies aged 11-13 months can learn words for things that are not visible. We first tested whether babies can learn the names of hidden objects that they had never seen before.\n\nThere were three conditions in this study: Experimental, Control, and Switch Word. If you were in the Experimental condition, on each trial, an experimenter first pointed to and named 3 familiar objects from the same category, for example, fruits. The experimenter then named a fourth object, which was not visible to the baby (a modi). But because the 3 familiar objects were fruits, the baby may have inferred that the last object was a fruit too.\n\nDuring Test, babies saw two objects that they are less likely to be familiar with—one from the same category (a dragon fruit) and one from a different category (an ottoman)–and heard a prompt, Where is the modi?. We measure where babies look when they hear this prompt. If babies can imagine an object that they have never seen and learn a word for it (modi), they should look to the object from the same category (the dragonfruit).\n\nBecause babies may look at the other object for unrelated reasons (prior knowledge, individual visual preferences, etc.), we average looking behavior across many babies and analyze those averages.\n\nIf you participated in the Control condition, the experimenter pointed to and named 3 familiar objects from different categories. In this condition, babies have no particular reason to prefer to look to one object over the other. This condition allows us to test the possibility that babies in the Experimental condition look to the correct object just because of their personal preference for particular objects. \n\nIf you participated in the Switch Word condition, the experimenter showed the same objects as in the Experimental condition, but babies heard a different unfamiliar word during test (e.g., dax instead of modi). This condition allows us to test the possibility that babies look to the correct object without knowing its name. \n\nImportantly, because babies at 11-13 months typically don't have word knowledge sufficient to use the information we provide in the above experiment, we asked you to read a digital picture book to your baby for a week – to boost his or her vocabulary. We then tested your baby's word knowledge and also repeated the same activity they did on the very first day to see if reading the book improved their word learning for hidden objects.\n\n<b>Compensation</b>\n<ul><li>To thank you for your participation, we will be emailing you a $10 Amazon card within a week.</li><li>We will use the email you provided in the Qualtrics survey.</li><li>If you do not hear from within a week, feel free to reach out!</li><li>If you participate again with another baby in the age range, you will receive another gift card!</li></ul>"

// Define common frames

var frames = {

"eligibility-survey": {

"kind": "exp-lookit-survey",

"formSchema": {

"schema": {

"type": "object",

"title": "Eligibility survey",

"properties": {

"USbased": {

"enum": [

"yes",

"no"

],

"type": "string",

"title": "Are you located in the United States?",

"required": true

}

}

},

"options": {

"fields": {

"USbased": {

"type": "radio",

"message": "Please answer this question.",

"validator": "required-field"

}

}

}

},

"nextButtonText": "Continue",

"showPreviousButton": false

},

"study-procedure": {

"kind": "exp-frame-select",

"frameOptions": [{

"kind": "exp-frame-select",

"showPreviousButton": false,

"frameOptions": [{

"kind": "exp-lookit-text",

"showPreviousButton": false,

"blocks": [{

"emph": true,

"text": "You are eligible to participate in this study"

},

{

"text": "Please click on the 'Next' button."

}

]

}]

},

{

"kind": "exp-lookit-text",

"showPreviousButton": false,

"blocks": [{

"emph": true,

"text": "We are currently unable to run participants located outside the United States."

},

{

"text": "Thank you for your interest in our research. We are working on expanding our international reach. We will contact you when we begin recruiting participants from other countries."

}

]

}

],

"generateProperties": "function(expData, sequence, child, pastSessions) {var formData = expData['0-eligibility-survey'].formData; if (formData.USbased == 'yes') { console.log('eligible'); return { 'whichFrames': 0, 'ELIGIBLE': true } } else { console.log('ineligible'); return { 'whichFrames': 1, 'ELIGIBLE': false } } }"

},

"study-procedure2": {

"kind": "exp-frame-select",

"frameOptions": [{

"kind": "exp-lookit-text",

"blocks": [{

"emph": true,

"text": "Next steps"

}]

}],

"selectNextFrame": "function(frames, frameIndex, frameData, expData, sequence, child, pastSessions) {var formData = expData['0-eligibility-survey'].formData; if (formData.USbased == 'no') {return frameIndex.length-2;} else {return frameIndex + 2;}}"

},

"study-intro-none": {

"kind": "exp-lookit-text",

"blocks": [{

"emph": true,

"text": "Thank you for your interest in our study 'Picture book for better word learning'! We are currently not enrolling new participants in this longitudinal study. We expect to begin recruiting a new cohort of participants soon. We are looking forward to your participation! Please revisit our study page in a few weeks."

}, ],

"showPreviousButton": false

},

"study-intro-first": {

"kind": "exp-lookit-text",

"blocks": [{

"emph": true,

"text": "Important: your baby does not need to be with you until the videos begin. First, let's go over what will happen!",

"title": "Overview of the 'Picture book for better word learning' study"

},

{

"text": "During this longitudinal study your baby will first see videos of an experimenter introducing colorful objects and naming them. She then will name a hidden object."

},

{

"text": "After that, we will ask you to read a digital picture book to your baby once oper day, for 7 consecutive days."

},

{

"text": "This is done to boost your baby's vocabulary before we proceed to the final part of the experiment."

},

{

"text": "After all 7 reading sessions are complete, you will be asked to log into the study one last time and complete the final experiment."

},

{

"text": "During the final experiment, your baby will first see images from the picture book and hear an experimenter's voice asking him or her to look at one of those images. We will also repeat the same activity your baby completed on the first day."

},

{

"text": "We are investigating (1) whether babies at 11-13 months can learn the name of the hidden object and (2) whether boosting their vocabulary can help them learn it better."

}

],

"showPreviousButton": false

},

"study-intro-last": {

"kind": "exp-lookit-text",

"blocks": [{

"emph": true,

"text": "You have completed all reading sessions. This is the final part of the 'Picture book for better word learning' study.",

"title": "Welcome back!"

},

{

"text": "During this part of the study, we will first test your baby's knowledge of the words they learned from the digital picture book. We will then repeat the same activity you and your baby did on the very first day, in which an experimenter names different objects, including one that cannot be seen."

},

{

"text": "After reading the instructions you’ll start the experiment when you and your baby are ready."

},

{

"text": "This experimental session will take about 10 minutes."

},

{

"text": "After the videos, you will answer a few final questions. Then you're all done!"

}

],

"showPreviousButton": false

},

"welcome": {

"kind": "exp-lookit-text",

"blocks": [{

"emph": true,

"text": "In this part of the study, we ask you to read our digital picture book to your child once per day for 7 consecutive days.",

"title": "Welcome back!"

},

{

"text": "Please go through every page within each daily reading session. Please read aloud the prompts written in the box 'For parents' at an infant-appropriate pace. You may deviate from the promts as long as you name each object at least 3 times."

},

{

"text": "Your reading sessions will be recorded. Please do not record more than one reading session per day."

},

{

"text": welcome_text

},

{

"emph": true,

"text": "Please remember to fill out the exit survey at the end of the reading session – this is necessary for us to mark your reading session as complete."

}

],

"showPreviousButton": false

},

"video-config": {

"kind": "exp-video-config",

"troubleshootingIntro": "Please follow the links below for additional help and troubleshooting."

},

"video-consent": {

"kind": "exp-lookit-video-consent",

"template": "consent_005",

"PIName": "Dr. Sandra Waxman",

"PIContact": "Elena Luchkina at elena.luchkina@northwestern.edu",

"institution": "Northwestern University",

"omit_injury_phrase": true,

"summary_statement": "\n\n<b>Title of Research Study:<i> Developmental origins and downstream consequences of verbal reference</i></b>\n\n<b>IRB Study Number:</b> STU00214867\n<b>Principal Investigator:</b> Sandra Waxman\n<b>Supported By:</b> This research is supported by the Psychology Department at Weinberg College of Arts and Sciences at Northwestern University and the National Institutes of Health (NIH).\n<b>Financial Interest Disclosure:</b> There is no conflict of interest.\n<h2>Key Information about this research study:</h2>This is a short summary of this study to help you decide whether to permit your child to be a part of this study:\n<ul><li>The purpose of this study is to better understand the link between language and thought in infancy and childhood. We will measure babies' looking direction to understand what they learned about the new words. </li><li>Your child will be asked to watch a short video or slide show. You will be asked to complete a survey about your child’s vocabulary.</li><li>This longitudinal study takes 9 days to complete. On each day, the study will take about 10 minutes. After the study, you may be contacted by email two times over the course of a year to complete the vocabulary survey again.</li><li>Data is stored securely on Lookit servers and by researchers at Northwestern. However, there is always a small risk that data transmitted over the internet may be intercepted or that the security of stored data may be compromised.</li><li>During the study, you and your child will be audio- and videotaped. </li></ul><b><i>Please note: If you or your child do not wish to be recorded, it is not possible for your child to be in this study.</i></b>\n<ul><li>The vocabulary survey is completed via a Qualtrics form hosted by the Infant and Child Development Center at Northwestern University. Qualtrics is an online survey platform accessed via a secure connection. Terms of service, addressing confidentiality, may be viewed at https://www.qualtrics.com/support/survey-platform/getting-started/data-protection-privacy/.</li><li>Please refer to the <a href='https://lookit.mit.edu/termsofuse/' target='_blank'>Lookit Terms of Use</a> (https://lookit.mit.edu/termsofuse/) for more detail on our use of the Lookit platform.</li><li>To review Lookit’s Privacy Statement again, please use this link <a href='https://lookit.mit.edu/privacy/' target='_blank'>Privacy Statement</a> (https://lookit.mit.edu/privacy/).</li></ul><b><i>Please note: If you do not consent to share your and your child’s data on the Lookit server, it is not possible for your child to be in this study.</i></b>\n<h2>Why am I being asked to take part in this research study?</h2>We are asking you to take part in this research study because your child is of the appropriate age and meets language and health eligibility criteria.\n<h2>How many people will be in this study?</h2>We expect about 80 people will be in this research study.",

"purpose": "<ul><li>Whether or not you take part is up to you.</li><li>You can choose not to take part.</li><li>You can agree to take part and later change your mind.</li><li>Your decision will not be held against you.</li></ul>",

"procedures": "Your child will watch a short video or slide show which will focus on either (1) communicative (e.g., language) or non-communicative (e.g., music) sounds, or (2) moving objects and characters. You will be asked to complete a survey about your child’s vocabulary. After the study you may be contacted by email two times over the course of the year to complete the vocabulary survey again.",

"risk_statement": "Data is stored securely on Lookit servers and by researchers at Northwestern. However, there is always a small risk that data transmitted over the internet may be intercepted or that the security of stored data may be compromised.",

"include_databrary": false,

"voluntary_participation": "You can decide not to participate in this research, or you can start the study and then decide to leave the study at any point and it will not be held against you. To do so, simply close the browser window. Any data already collected will not be saved.",

"datause": "<ul><li>Upon receiving results of your survey, any possible identifiers will be deleted.</li><li>You will be identified only by a unique subject number.</li><li>Your email address will be stored separately from your survey data</li><li>All information will be kept on a secure server only accessible by the research team.</li><li>The results of the research study may be published, but your name will not be used.</li></ul>Please refer to the <a href='https://lookit.mit.edu/termsofuse/' target='_blank'>Lookit Terms of Use</a> for more detail on our use of the Lookit platform.\n<h2>Certificate of Confidentiality</h2> This research is covered by a Certificate of Confidentiality from the National Institutes of Health. This means that the researchers cannot release or use information, documents, or samples that may identify you in any action or suit unless you say it is okay. They also cannot provide them as evidence unless you have agreed. This protection includes federal, state, or local civil, criminal, administrative, legislative, or other proceedings. An example would be a court subpoena.\n\nIdentifiable information that could still be disclosed beyond the research team: The Certificate does not stop reporting that federal, state or local laws require. Some examples are laws that require reporting of child or elder abuse, some communicable diseases, and threats to harm yourself or others. The Certificate cannot be used to stop a sponsoring United States federal or state government agency from checking records or evaluating programs. The Certificate does not stop disclosures required by the federal Food and Drug Administration (FDA). The Certificate also does not prevent your information from being used for other research if allowed by federal regulations.\n\nResearchers may release information about you when you say it is okay. For example, you may give them permission to release information to insurers, medical providers or any other persons not connected with the research. The Certificate of Confidentiality does not stop you from willingly releasing information about your involvement in this research. It also does not prevent you from having access to your own information.",

"gdpr": false,

"research_rights_statement": "This research has been reviewed and approved by an Institutional Review Board (“IRB”). You may talk to them at (312) 503-9338 or <a href = 'mailto: irb@northwestern.edu'>irb@northwestern.edu</a> if:<ul><li>Your questions, concerns, or complaints are not being answered by the research team</li><li>You cannot reach the research team</li><li>You want to talk to someone besides the research team</li><li>You have questions about your rights as a research participant</li><li>You want to get information or provide input about this research.</li></ul>",

"payment": "If you agree to take part in this research study, after you finish the study and if you meet these eligibility criteria, we will email you a $5 Amazon gift card after you compete 7 book reading sessions within 1 week. After you complete the final test (on day 9), we will send you a $10 Amazon gift card within 1 week. We will send the card even if we are not able to use your child’s data. \n\nTo be eligible for compensation, the following criteria must be met:<ul><li>Your child must be in the age range for this study;</li><li>You need to submit a valid video consent statement;</li><li>We need to see that there is a child with you in the study participation video.</li></ul>",

"benefits_header": "What else do I need to know?",

"purpose_header": "What should I know about a research study?",

"procedures_header": "If you say that “Yes, you want to be in this research,” here is what you will do:",

"risk_header": "Is there any way being in this study could be bad for me?",

"additional_segments": [{

"text": "Efforts will be made to limit the use and disclosure of your personal information, including research study records, to people who have a need to review this information.",

"title": "What happens to the information collected for the research?"

},

{

"text": "If you want a copy of this consent for your records, press “download” and save the document to your computer.",

"title": "Saving a copy of the consent form"

}

]

},

"qualtrics": {

"kind": "exp-lookit-text",

"blocks": [

{

"emph": false,

"text": survey_text,

"title": "Your child's Lookit ID is:"

},

{

"emph": true,

"text": "<iframe width='800' height='600' src='https://northwestern.az1.qualtrics.com/jfe/form/SV_7OIkk149rvhkah0'></iframe>\n\nPlease remember to hit the purple button 'Submit' in the bottom of the survey.\n\nIf the survey does not show up in the window above, please click <u><a href='https://northwestern.az1.qualtrics.com/jfe/form/SV_a2Ai4ClETwc5yW9' target='_blank' rel='noopener'>here</a></u> and return to this tab after completing the survey.",

"title": "Before we begin, please fill out this survey. We will use the email you provide in this survey to send you Amazon gift cards."

}

],

"showPreviousButton": false

},

"instructions-first": {

"kind": "exp-lookit-instructions",

"blocks": [{

"title": "Instructions",

"text": "This is the initial part of the study, before the picture book reading begins. Before the initial experiment, we will ask you to fill out a Qualtrics survey. This will take 5-10 minutes. Then, the video section (the experiment) will begin. The video section will take about 3 minutes to complete. After that, you will be able to select the level of privacy for your data."

},

{

"title": "Study overview",

"listblocks": [{

"text": "To get your baby's attention, first they will see a moving shape and hear a chime. "

},

{

"text": "Then your baby will watch four videos, each about 45 seconds long."

}

]

},

{

"title": "During the videos",

"listblocks": [{

"text": "Please face away from the screen, holding your baby so they can look over your shoulder. Please don't look at the videos yourself – we may not be able to use your baby’s data in that case.",

"image": {

"alt": "Father holding child looking over his shoulder",

"src": "https://s3.amazonaws.com/lookitcontents/exp-physics/OverShoulder.jpg"

}

},

{

"text": "This is because your baby is learning from you all the time and may pick up on even very small cues about what you think. But if you can't see the stimuli, we're definitely only measuring your baby's own beliefs."

},

{

"text": "Don’t worry if your baby isn’t looking at the screen the entire time! Please just try to keep them facing the screen so they can look if they want to."

}

]

},

{

"title": "Pausing and stopping",

"listblocks": [{

"text": "If your baby gets fussy or distracted, or you need to attend to something else for a moment, you can pause the study by pressing the space bar."

},

{

"text": "If you need to end the study early, try closing the window or tab and you should see an 'exit' option pop up. You’ll be prompted to note any technical problems you might be experiencing and to select a privacy level for your videos."

}

]

},

{

"text": "Please turn the volume up so it's easy to hear but still comfortable.",

"title": "Test your audio",

"mediaBlock": {

"text": "You should hear 'Ready... go?'",

"isVideo": false,

"sources": [{

"src": "https://fragmean.s3.us-east-2.amazonaws.com/Ready.mp3",

"type": "audio/mp3"

},

{

"src": "OGG_SOURCE_HERE",

"type": "audio/ogg"

}

],

"mustPlay": true,

"warningText": "Please try playing the sample audio."

}

}

],

"nextButtonText": "Next"

},

"instructions-last": {

"kind": "exp-lookit-instructions",

"blocks": [{

"title": "Instructions",

"text": "This is the final part of our longitudinal study. Thank you for completing all reading sessions and returning for the final part!"

},

{

"title": "Study overview",

"listblocks": [{

"text": "First, your baby will see a series of objects from the book and hear an experimenter's voice asking them to look at one of the objects. After that, your baby will see the same videos that they saw in the very first part of the study."

},

{

"text": "In total, the entire procedure will take about 10 minutes to complete."

}

]

},

{

"title": "During the videos",

"listblocks": [{

"text": "Please face away from the screen, holding your baby so they can look over your shoulder. Please don't look at the videos yourself--we may not be able to use your baby’s data in that case.",

"image": {

"alt": "Father holding child looking over his shoulder",

"src": "https://s3.amazonaws.com/lookitcontents/exp-physics/OverShoulder.jpg"

}

},

{

"text": "This is because your baby is learning from you all the time, and may pick up on even very small cues about what you think. But if you can't see the stimuli, we're definitely only measuring your baby's own beliefs."

},

{

"text": "Don’t worry if your baby isn’t looking at the screen the entire time! Please just try to keep them facing the screen so they can look if they want to."

}

]

},

{

"title": "Pausing and stopping",

"listblocks": [{

"text": "If your baby gets fussy or distracted, or you need to attend to something else for a moment, you can pause the study by pressing the space bar."

},

{

"text": "If you need to end the study early, try closing the window or tab and you should see an 'exit' option pop up. You’ll be prompted to note any technical problems you might be experiencing and to select a privacy level for your videos."

}

]

},

{

"text": "Please turn the volume up so it's easy to hear but still comfortable.",

"title": "Test your audio",

"mediaBlock": {

"text": "You should hear 'Ready... go?'",

"isVideo": false,

"sources": [{

"src": "https://fragmean.s3.us-east-2.amazonaws.com/Ready.mp3",

"type": "audio/mp3"

},

{

"src": "OGG_SOURCE_HERE",

"type": "audio/ogg"

}

],

"mustPlay": true,

"warningText": "Please try playing the sample audio."

}

}

],

"nextButtonText": "Next"

},

"video-preview": {

"kind": "exp-lookit-stimuli-preview",

"blocks": [{

"title": "Video preview",

"text": "If you'd like to see the videos your baby will be shown, you can take a look ahead of time now. It's important that you preview the videos without your baby, so that the videos will still be new to them.\n\nIf you choose to skip, press 'Skip preview'."

}],

"stimuli": [{

"video": [{

"src": "https://fragmean.s3.us-east-2.amazonaws.com/Miriam_animals_final_short.mp4",

"type": "video/mp4"

}],

"caption": "For each trial, there will be a woman on the screen pointing at images of objects. Here's an example.\n\nAs soon as you press 'Continue', please turn around and hold your baby over your shoulder."

}],

"videoTypes": [

"webm",

"mp4"

],

"baseDir/ext": "https://fragmean.s3.us-east-2.amazonaws.com/",

"skipButtonText": "Skip preview",

"previewButtonText": "I'd like to preview the videos",

"showPreviousButton": true

},

"webcam-display-break": {

"kind": "exp-lookit-webcam-display",

"blocks": [{

"title": "Video check:",

"listblocks": [{

"text": "Please turn around and hold your baby over your shoulder right after pressing 'Next'. The study will begin promptly."

},

{

"text": "Please check that your baby is visible."

},

{

"text": "Unless you need to pause the study (hit the 'space' bar to pause), please remain in this position until you hear 'All done'."

},

{

"text": "If the video is 'stuck' and is no longer playing, press the 'space' bar to pause the trial. Press the 'space' bar again to restart it."

}

]

}],

"nextButtonText": "Next",

"showPreviousButton": false,

"displayFullscreenOverride": true,

"startRecordingAutomatically": false

},

"fam-trials": {

"kind": "choice",

"baseDir": "https://www.mit.edu/~kimscott/placeholderstimuli/",

"sampler": "permute",

"commonFrameProperties": {

"kind": "exp-lookit-images-audio",

"audioTypes": [

"ogg",

"mp3"

],

"audio": [{

"src": "https://fragmean.s3.us-east-2.amazonaws.com/magic-chime-01.mp3",

"type": "audio/mp3"

}],

"autoProceed": false,

"doRecording": true,

"showProgressBar": true,

"maximizeDisplay": true,

"showCursor": true,

"pageColor": "white",

"backgroundColor": "white",

"showPreviousButton": false

},

"frameOptions": [{

"images": [{

"id": "Apple",

"src": "https://fragmean.s3.us-east-2.amazonaws.com/Apple.jpg",

"position": "fill"

}],

"parentTextBlock": {

"text": "Look! An apple! Do you see the apple? This is an apple.",

"title": "For parents"

}

},

{

"images": [{

"id": "Banana",

"src": "https://fragmean.s3.us-east-2.amazonaws.com/Banana.jpg",

"position": "fill"

}],

"parentTextBlock": {

"text": "Look! A banana! Do you see the banana? This is a banana.",

"title": "For parents"

}

},

{

"images": [{

"id": "Bowl",

"src": "https://fragmean.s3.us-east-2.amazonaws.com/Bowl.jpg",

"position": "fill"

}],

"parentTextBlock": {

"text": "Look! A bowl! Do you see the bowl? This is a bowl.",

"title": "For parents"

}

},

{

"images": [{

"id": "Bus",

"src": "https://fragmean.s3.us-east-2.amazonaws.com/Bus.jpg",

"position": "fill"

}],

"parentTextBlock": {

"text": "Look! A bus! Do you see the bus? This is a bus.",

"title": "For parents"

}

},

{

"images": [{

"id": "Car",

"src": "https://fragmean.s3.us-east-2.amazonaws.com/Car.jpg",

"position": "fill"

}],

"parentTextBlock": {

"text": "Look! A car! Do you see the car? This is a car.",

"title": "For parents"

}

},

{

"images": [{

"id": "Cat",

"src": "https://fragmean.s3.us-east-2.amazonaws.com/Cat.jpg",

"position": "fill"

}],

"parentTextBlock": {

"text": "Look! A cat! Do you see the cat? This is a cat.",

"title": "For parents"

}

},

{

"images": [{

"id": "Chair",

"src": "https://fragmean.s3.us-east-2.amazonaws.com/Chair.jpg",

"position": "fill"

}],

"parentTextBlock": {

"text": "Look! A chair! Do you see the chair? This is a chair.",

"title": "For parents"

}

},

{

"images": [{

"id": "Cup",

"src": "https://fragmean.s3.us-east-2.amazonaws.com/Cup.jpg",

"position": "fill"

}],

"parentTextBlock": {

"text": "Look! A cup! Do you see the cup? This is a cup.",

"title": "For parents"

}

},

{

"images": [{

"id": "Dog",

"src": "https://fragmean.s3.us-east-2.amazonaws.com/Dog.jpg",

"position": "fill"

}],

"parentTextBlock": {

"text": "Look! A dog! Do you see the dog? This is a dog.",

"title": "For parents"

}

},

{

"images": [{

"id": "Fork",

"src": "https://fragmean.s3.us-east-2.amazonaws.com/Fork.jpg",

"position": "fill"

}],

"parentTextBlock": {

"text": "Look! A fork! Do you see the fork? This is a fork.",

"title": "For parents"

}

},

{

"images": [{

"id": "Glass",

"src": "https://fragmean.s3.us-east-2.amazonaws.com/Glass.JPG",

"position": "fill"

}],

"parentTextBlock": {

"text": "Look! A glass! Do you see the glass? This is a glass.",

"title": "For parents"

}

},

{

"images": [{

"id": "Hammer",

"src": "https://fragmean.s3.us-east-2.amazonaws.com/Hammer.jpg",

"position": "fill"

}],

"parentTextBlock": {

"text": "Look! A hammer! Do you see the hammer? This is a hammer.",

"title": "For parents"

}

},

{

"images": [{

"id": "Hat",

"src": "https://fragmean.s3.us-east-2.amazonaws.com/Hat.jpg",

"position": "fill"

}],

"parentTextBlock": {

"text": "Look! A hat! Do you see the hat? This is a hat.",

"title": "For parents"

}

},

{

"images": [{

"id": "Horse",

"src": "https://fragmean.s3.us-east-2.amazonaws.com/Horse.jpg",

"position": "fill"

}],

"parentTextBlock": {

"text": "Look! A horse! Do you see the horse? This is a horse.",

"title": "For parents"

}

},

{

"images": [{

"id": "Jacket",

"src": "https://fragmean.s3.us-east-2.amazonaws.com/Jacket.jpg",

"position": "fill"

}],

"parentTextBlock": {

"text": "Look! A jacket! Do you see the jacket? This is a jacket.",

"title": "For parents"

}

},

{

"images": [{

"id": "Knife",

"src": "https://fragmean.s3.us-east-2.amazonaws.com/Knife.jpg",

"position": "fill"

}],

"parentTextBlock": {

"text": "Look! A knife! Do you see the knife? This is a knife.",

"title": "For parents"

}

},

{

"images": [{

"id": "Orange",

"src": "https://fragmean.s3.us-east-2.amazonaws.com/Orange.jpg",

"position": "fill"

}],

"parentTextBlock": {

"text": "Look! An orange! Do you see the orange? This is an orange.",

"title": "For parents"

}

},

{

"images": [{

"id": "Saw",

"src": "https://fragmean.s3.us-east-2.amazonaws.com/Saw.jpg",

"position": "fill"

}],

"parentTextBlock": {

"text": "Look! A saw! Do you see the saw? This is a saw.",

"title": "For parents"

}

},

{

"images": [{

"id": "Sock",

"src": "https://fragmean.s3.us-east-2.amazonaws.com/Sock.jpg",

"position": "fill"

}],

"parentTextBlock": {

"text": "Look! A sock! Do you see the sock? This is a sock.",

"title": "For parents"

}

},

{

"images": [{

"id": "Sofa",

"src": "https://fragmean.s3.us-east-2.amazonaws.com/Sofa.jpg",

"position": "fill"

}],

"parentTextBlock": {

"text": "Look! A sofa! Do you see the sofa? This is a sofa.",

"title": "For parents"

}

},

{

"images": [{

"id": "Spoon",

"src": "https://fragmean.s3.us-east-2.amazonaws.com/Spoon.jpg",

"position": "fill"

}],

"parentTextBlock": {

"text": "Look! A spoon! Do you see the spoon? This is a spoon.",

"title": "For parents"

}

},

{

"images": [{

"id": "Truck",

"src": "https://fragmean.s3.us-east-2.amazonaws.com/Truck.jpg",

"position": "fill"

}],

"parentTextBlock": {

"text": "Look! A truck! Do you see the truck? This is a truck.",

"title": "For parents"

}

},

{

"images": [{

"id": "Wrench",

"src": "https://fragmean.s3.us-east-2.amazonaws.com/Wrench.jpg",

"position": "fill"

}],

"parentTextBlock": {

"text": "Look! A wrench! Do you see the wrench? This is a wrench.",

"title": "For parents"

}

}

]

},

"fragmean-trials": {

"kind": "choice",

"baseDir": "https://www.mit.edu/~kimscott/placeholderstimuli/",

"sampler": "random-parameter-set",

"frameList": [{

"video": {

"loop": false,

"position": "fill",

"source": "VIDEO1"

},

"doRecording": true,

"requireVideoCount": 1,

"requireAudioCount": 0

},

{

"video": {

"loop": false,

"position": "fill",

"source": "VIDEO2"

},

"doRecording": true,

"requireVideoCount": 1,

"requireAudioCount": 0

},

{

"video": {

"loop": false,

"position": "fill",

"source": "VIDEO3"

},

"doRecording": true,

"requireVideoCount": 1,

"requireAudioCount": 0

},

{

"video": {

"loop": false,

"position": "fill",

"source": "VIDEO4"

},

"doRecording": true,

"requireVideoCount": 1,

"requireAudioCount": 0

},

{

"video": {

"loop": true,

"top": 40,

"left": 45,

"width": 10,

"source": [{

"src": "https://fragmean.s3.us-east-2.amazonaws.com/attentiongrabber.webm",

"type": "video/webm"

}]

},

"audio": {

"loop": false,

"source": "all_done_HO"

},

"doRecording": false,

"requireAudioCount": 1,

"requireVideoCount": 0

}

],

"parameterSets": [{

"VIDEO1": [{

"src": "https://fragmean.s3.us-east-2.amazonaws.com/Follow-up+control+fruits.mp4",

"type": "video/mp4"

}],

"VIDEO2": [{

"src": "https://fragmean.s3.us-east-2.amazonaws.com/Follow-up+control+vehicles.mp4",

"type": "video/mp4"

}],

"VIDEO3": [{

"src": "https://fragmean.s3.us-east-2.amazonaws.com/Follow-up+control+animals.mp4",

"type": "video/mp4"

}],

"VIDEO4": [{

"src": "https://fragmean.s3.us-east-2.amazonaws.com/Follow-up+control+clothing.mp4",

"type": "video/mp4"

}]

},

{

"VIDEO2": [{

"src": "https://fragmean.s3.us-east-2.amazonaws.com/Follow-up+control+fruits.mp4",

"type": "video/mp4"

}],

"VIDEO3": [{

"src": "https://fragmean.s3.us-east-2.amazonaws.com/Follow-up+control+vehicles.mp4",

"type": "video/mp4"

}],

"VIDEO4": [{

"src": "https://fragmean.s3.us-east-2.amazonaws.com/Follow-up+control+animals.mp4",

"type": "video/mp4"

}],

"VIDEO1": [{

"src": "https://fragmean.s3.us-east-2.amazonaws.com/Follow-up+control+clothing.mp4",

"type": "video/mp4"

}]

},

{

"VIDEO3": [{

"src": "https://fragmean.s3.us-east-2.amazonaws.com/Follow-up+control+fruits.mp4",

"type": "video/mp4"

}],

"VIDEO4": [{

"src": "https://fragmean.s3.us-east-2.amazonaws.com/Follow-up+control+vehicles.mp4",

"type": "video/mp4"

}],

"VIDEO1": [{

"src": "https://fragmean.s3.us-east-2.amazonaws.com/Follow-up+control+animals.mp4",

"type": "video/mp4"

}],

"VIDEO2": [{

"src": "https://fragmean.s3.us-east-2.amazonaws.com/Follow-up+control+clothing.mp4",

"type": "video/mp4"

}]

},

{

"VIDEO4": [{

"src": "https://fragmean.s3.us-east-2.amazonaws.com/Follow-up+control+fruits.mp4",

"type": "video/mp4"

}],

"VIDEO1": [{

"src": "https://fragmean.s3.us-east-2.amazonaws.com/Follow-up+control+vehicles.mp4",

"type": "video/mp4"

}],

"VIDEO2": [{

"src": "https://fragmean.s3.us-east-2.amazonaws.com/Follow-up+control+animals.mp4",

"type": "video/mp4"

}],

"VIDEO3": [{

"src": "https://fragmean.s3.us-east-2.amazonaws.com/Follow-up+control+clothing.mp4",

"type": "video/mp4"

}]

}

],

"commonFrameProperties": {

"kind": "exp-lookit-video",

"baseDir": "https://www.mit.edu/~kimscott/intermodal/",

"audioTypes": ["ogg",

"mp3"

],

"videoTypes": ["webm",

"mp4"

],

"backgroundColor": "white",

"autoProceed": true,

"pauseAudio": [{

"src": "https://fragmean.s3.us-east-2.amazonaws.com/pause.mp3"

}],

"pauseVideo": [{

"src": "https://fragmean.s3.us-east-2.amazonaws.com/attentiongrabber.webm",

"type": "video/webm"

}],

"unpauseAudio": [{

"src": "https://fragmean.s3.us-east-2.amazonaws.com/return_after_pause.mp3O"

}]

}

},

"calibration": {

"kind": "exp-lookit-calibration",

"baseDir": "https://www.mit.edu/~kimscott/placeholderstimuli/",

"dorecording": true,

"showWaitForUploadMessage": false,

"waitForUploadImage": [],

"audioTypes": [

"ogg",

"mp3"

],

"videoTypes": [

"webm",

"mp4"

],

"calibrationLength": 2000,

"calibrationAudio": [{

"src": "https://fragmean.s3.us-east-2.amazonaws.com/chimes.mp3"

}],

"calibrationVideo": [{

"src": "https://fragmean.s3.us-east-2.amazonaws.com/attentiongrabber.webm",

"type": "video/webm"

}],

"calibrationPositions": [

"left",

"right",

"left",

"right",

"center"

]

},

"exit-survey-first": {

"kind": "exp-lookit-exit-survey",

"showDatabraryOptions": false,

"debriefing": {

"title": "Thank you for signing up!",

"blocks": [{

"listblocks": [{

"text": exit_first

}]

}]

}

},

"exit-survey-fam": {

"kind": "exp-lookit-exit-survey",

"showDatabraryOptions": false,

"debriefing": {

"title": "Thank you for completing today's reading session!",

"blocks": [{

"listblocks": [{

"text": exit_fam_only

},

{

"text": "Please log back tomorrow.\n\nIf you have completed all reading sessions, the next time you log in, you will be prompted to the final part of the study."

}

]

}]

}

},

"exit-survey-last": {

"kind": "exp-lookit-exit-survey",

"showDatabraryOptions": false,

"debriefing": {

"title": "Thank you for helping our research!",

"blocks": [{

"listblocks": [{

"text": exit_last

}]

}]

}

}

}

// start at a random point in this list and cycle through across trials.

// each element is a list: category1, category2, audio.

// category1 and category2 match up to keys in available_images; audio

// should be filenames in baseDir/mp3

var all_category_pairings = [

[

"apple",

"filler",

"Apple"

],

[

"banana",

"filler",

"Banana"

],

[

"orange",

"filler",

"Orange"

],

[

"dog",

"filler",

"Dog"

],

[

"cat",

"filler",

"Cat"

],

[

"horse",

"filler",

"Horse"

],

[

"bus",

"filler",

"Bus"

],

[

"car",

"filler",

"Car"

],

[

"truck",

"filler",

"Truck"

],

[

"hat",

"filler",

"Hat"

],

[

"sock",

"filler",

"Sock"

],

[

"jacket",

"filler",

"Jacket"

]

]

// Every image is just used once total, either as a target or as a distractor.

// We'll remove the images from these lists as they get used.

var available_images = {

"apple": ["https://fragmean.s3.us-east-2.amazonaws.com/Apple.jpg"],

"banana": ["https://fragmean.s3.us-east-2.amazonaws.com/Banana.jpg"],

"orange": ["https://fragmean.s3.us-east-2.amazonaws.com/Orange.jpg"],

"bus": ["https://fragmean.s3.us-east-2.amazonaws.com/Bus.jpg"],

"car": ["https://fragmean.s3.us-east-2.amazonaws.com/Car.jpg"],

"truck": ["https://fragmean.s3.us-east-2.amazonaws.com/Truck.jpg"],

"cat": ["https://fragmean.s3.us-east-2.amazonaws.com/Cat.jpg"],

"dog": ["https://fragmean.s3.us-east-2.amazonaws.com/Dog.jpg"],

"horse": ["https://fragmean.s3.us-east-2.amazonaws.com/Horse.jpg"],

"hat": ["https://fragmean.s3.us-east-2.amazonaws.com/Hat.jpg"],

"sock": ["https://fragmean.s3.us-east-2.amazonaws.com/Sock.jpg"],

"jacket": ["https://fragmean.s3.us-east-2.amazonaws.com/Jacket.jpg"],

"filler": [

"https://fragmean.s3.us-east-2.amazonaws.com/Bowl.jpg",

"https://fragmean.s3.us-east-2.amazonaws.com/Glass.JPG",

"https://fragmean.s3.us-east-2.amazonaws.com/Cup.jpg",

"https://fragmean.s3.us-east-2.amazonaws.com/Chair.jpg",

"https://fragmean.s3.us-east-2.amazonaws.com/Sofa.jpg",

"https://fragmean.s3.us-east-2.amazonaws.com/Table.jpg",

"https://fragmean.s3.us-east-2.amazonaws.com/Wrench.jpg",

"https://fragmean.s3.us-east-2.amazonaws.com/Hammer.jpg",

"https://fragmean.s3.us-east-2.amazonaws.com/Saw.jpg",

"https://fragmean.s3.us-east-2.amazonaws.com/Spoon.jpg",

"https://fragmean.s3.us-east-2.amazonaws.com/Fork.jpg",

"https://fragmean.s3.us-east-2.amazonaws.com/Knife.jpg"

]

};

var available_audio = {

"Apple": [{

"src": "https://fragmean.s3.us-east-2.amazonaws.com/Apple.mp3"

}],

"Banana": [{

"src": "https://fragmean.s3.us-east-2.amazonaws.com/Banana.mp3"

}],

"Orange": [{

"src": "https://fragmean.s3.us-east-2.amazonaws.com/Orange.mp3"

}],

"Bus": [{

"src": "https://fragmean.s3.us-east-2.amazonaws.com/Bus.mp3"

}],

"Car": [{

"src": "https://fragmean.s3.us-east-2.amazonaws.com/Car.mp3"

}],

"Truck": [{

"src": "https://fragmean.s3.us-east-2.amazonaws.com/Truck.mp3"

}],

"Cat": [{

"src": "https://fragmean.s3.us-east-2.amazonaws.com/Cat.mp3"

}],

"Dog": [{

"src": "https://fragmean.s3.us-east-2.amazonaws.com/Dog.mp3"

}],

"Horse": [{

"src": "https://fragmean.s3.us-east-2.amazonaws.com/Horse.mp3"

}],

"Hat": [{

"src": "https://fragmean.s3.us-east-2.amazonaws.com/Hat.mp3"

}],

"Sock": [{

"src": "https://fragmean.s3.us-east-2.amazonaws.com/Sock.mp3"

}],

"Jacket": [{

"src": "https://fragmean.s3.us-east-2.amazonaws.com/Jacket.mp3"

}]

};

// Make a deep copy of the original available images, in case we run out

// (e.g. after adding additional trials) and need to "refill" a category.

var all_images = Ember.$.extend(true, {}, available_images)

// Choose a random starting point and order for the category pairings

var ordered_category_pairings = shuffle(all_category_pairings)

var frame_sequence_none = ['study-intro-none']

var frame_sequence_first = ['eligibility-survey', 'study-procedure', 'study-procedure2', 'study-intro-first', 'video-config', 'video-consent', 'qualtrics', 'instructions-first', 'video-preview', 'webcam-display-break', 'calibration', 'fragmean-trials', 'exit-survey-first']

var frame_sequence_book = ['welcome', 'video-config', 'video-consent', 'fam-trials', 'exit-survey-fam']

var frame_sequence_last = ['study-intro-last', 'video-config', 'video-consent', 'instructions-last', 'webcam-display-break', 'calibration']

for (iTrial = 0; iTrial < 12; iTrial++) {

var category_pairing = ordered_category_pairings[iTrial]

var category_id_1 = category_pairing[0]

var category_id_2 = category_pairing[1]

var audio = category_pairing[2];

// "Refill" available images if empty

if (!available_images[category_id_1].length) {

available_images[category_id_1] = all_images[category_id_1]

}

if (!available_images[category_id_2].length) {

available_images[category_id_2] = all_images[category_id_2]

}

var image1 = pop_random(available_images[category_id_1])

var image2 = pop_random(available_images[category_id_2])

var left_right_pairing = shuffle(["left", "right"])

thisTrial = {

"kind": "group",

"frameList": [{

"images": [{

"id": "apple",

"src": "https://fragmean.s3.us-east-2.amazonaws.com/Elmo.jpg",

"top": 47,

"left": 47,

"width": 5

}],

"durationSeconds": 2,

"doRecording": false

},

{

"audio": available_audio[audio],

"images": [{

"id": "option1-test",

"src": image1,

"position": left_right_pairing[0]

},

{

"id": "option2-test",

"src": image2,

"position": left_right_pairing[1]

}

],

"durationSeconds": 10,

"doRecording": true

}

],

"commonFrameProperties": {

"kind": "exp-lookit-images-audio",

"autoProceed": true,

"maximizeDisplay": true,

"backgroundColor": "white",

"pageColor": "white",

"showCursor": false,

"audioTypes": [

"mp3"

]

}

}

// Store this frame in frames and in the sequence

frameId = 'test-trial-' + (iTrial + 1)

frames[frameId] = thisTrial

frame_sequence_last.push(frameId)

}

if (count < 1) {

frame_seq = frame_sequence_first

} else if (count < 8) {

frame_seq = frame_sequence_book

} else {

frame_seq = frame_sequence_last

}

// Finish up the frame sequence with the exit survey

if (frame_seq == frame_sequence_last) {

frame_seq = frame_seq.concat(['fragmean-trials', 'exit-survey-last'])

}

// Return a study protocol with "frames" and "sequence" fields just like when

// defining the protocol in JSON only

return {

frames: frames,

sequence: frame_seq

}

}
