# Supplementary material for: Semantic priming supports infants’ ability to learn names of unseen objects
Source: PLoS One. 2025 Apr 23;20(4):e0321775. doi: 10.1371/journal.pone.0321775 (PMC12017536; doi:10.1371/journal.pone.0321775)
Supplement: Attachment — Submitted filename: Response to reviewers Final.pdf [file pone.0321775.s009.pdf]

Dear Dr. Lorett,

Please consider our revised manuscript PONE-D-24-17859 for publication in PLOS One. We are grateful to you for your and reviewers for such thoughtful feedback and for offering us the opportunity for revision. In what follows, we provide a point-by-point response to each reviewer's comments. We attach the revised manuscript and a track-changes copy.

We hope that you will agree that by responding to this very constructive set of reviews, we have now produced a manuscript that is acceptable for publication in PLOS One.

Sincerely,
Authors

Please note that page and line references are for the *clean copy* of the revised manuscript. The copy that contains tracked changes does not accurately reflect these references.

Reviewer #1

1. *Deviations from the registered protocol seem reasonable. The most significant deviation is the change of the analysis time window. Would the results be different if the original window had been chosen? Why did Ferguson use a longer window? Why is the "conventional" window the way it is? I think it would be good to be more explicit here.*

We are happy to provide a more explicit explanation for the deviations we implemented for the analysis time window (pp. 7-8, lines 151-158). We had initially thought to implement the same time window as in prior work in our lab (Ferguson et al., 2014), but subsequently realized that the current design was closer to other word-learning tasks that involve ostensive labeling (cf., Swingley & Aslin, 2000), except that in our design, the labelled object was hidden from view.

To clarify, here is how our design differs from Ferguson et al: in their design, to be able to respond to the audio prompt, infants had to recall the sentence in which the name of the novel object was embedded (e.g., "the *dax* is crying"), and to use the surrounding familiar words to infer the meaning of the novel noun. This incurred a greater processing time: looking trajectories across conditions began to diverge around 3250 ms. In our current investigation, infants did not have to rely on sentence processing: they could use object category to infer the meaning of the novel word. For this reason, we decided to use an analysis time window that matches those in most word learning designs (cf., Swingley & Aslin, 2000; we provide additional citations in the text). This window of analysis is based on the time infants typically require to process a word and initiate a gaze shift.

2. *Methods: How was the study implemented? Is the code available somewhere?*

We now provide the code in Appendix 5.

3. *Analysis: Why is there no random effect for subjects? Some, if not most, individuals have more than one trial.*

Thank you for raising this point. We did not include a random effect of subject because we employed a between subjects design. Therefore, adding a random effect for subjects would result in zero variance within Condition.

4. *In line 288, you give 1.84 as the t-value threshold; in 304, it's 1.69. Why are the values different? Which one was actually used?*

Thank you for catching this. The threshold was 1.69. We have now corrected our error.

5. *307: I would include the non-significant demographics in the final analysis nevertheless. Or what is the reason not to do so?*

Thank you for raising this question. We decided against including the non-significant demographics because many were categorical with multiple levels (these variables are converted into dummy variables, one per level, in regression analysis). Including these in the final analysis would increase the chance of accidental multicollinearity or an overly specified model (that cannot be supported by the data). Both issues would result in a singular model fit. This, coupled with a modest sample size (~22 infants per condition), motivated our decision to collapse across those variables after preliminary analyses (reported here) revealed no significant effects. However, we are willing to include the model suggested by the reviewer if the editor prefers.

6. *I'm not sure Experiment 2 is meaningful, given that you excluded twice as many infants as you included in the sample. It seems the procedure is too challenging for them.*

Thank you for pointing this out. We regret that in the original submission, we were not sufficiently clear about our criteria for inclusion. We have now corrected this oversight (p. 20, lines 452-453). We clarify that infants who did not participate in all 9 learning sessions were not fussy or inattentive. Instead, many caregivers (n=109, and despite our reminders) did not log in the 9 learning sessions. Their infants could not participate in the outcome assessment for this reason.

With this point clarified, we note here that we found no differences in either the number of look-aways or the distributions of mean %LT at baseline assessment. This provides assurances that caregivers' decision to complete the learning sessions did not reflect their infants' attention to the display or their responses to the experimental stimuli.

In our view, Experiment 2 is valuable for at least two reasons. First, it reveals that the processing costs of simultaneously representing an unseen object and learning a new word for it, are too steep for 12-month-old infants, even those who completed our vocabulary training. Second, the results of Experiment 2 suggest that infants' preferences in a forced choice looking task may be influenced by their familiarity with the semantic neighborhood of the test objects. These findings will likely be helpful to other researchers seeking to leverage infants' category knowledge and word learning in experimental contexts.

7. *502: Please report the correlations even if they are not significant.*

We now report the coefficient and the p-value for the non-significant correlation between infants' word knowledge before vocabulary training (MCDI) and after training (mean %LT to the target on vocabulary test trials) (p. 25, line 560).

8. *506: How was vocabulary knowledge computed? Was the data aggregated by infant or by infant and category?*

For each infant, we calculated the proportion of looking to the target object on each of 12 vocabulary test trials (see Figure 6). For the timecourse plots, we have aggregated the looking across time across infants and across each semantic neighborhood. With 12 trials in total, corresponding to the 12 word-object pairs tested, this means that we aggregated across infants and across 3 word-object pairs from each semantic neighborhood (see Figure 8).

9. *Discussion: I do not understand why children should look at the target in the no-priming condition (what they do). Any explanation? This result suggests something was off with the stimuli, the procedure, or the coding.*

Thank you for raising this question. We now discuss this in the revised manuscript. Because the coding was reliable for all conditions (88% consistency for human vs. iCatcher and 90% among human coders), we think it is unlikely that coding contributed this pattern of looking. Instead, the parallels in 12-month-olds' looking preference in all three conditions, including the No Priming condition, are more likely related to our choice of target and distractor objects.

Recall that we selected these from the semantic neighborhoods that are most familiar to infants. We expected that this would maximize the power of our semantic priming manipulation. But as we now discuss, it is possible that this affected 12-month-olds in another way: they may have preferred objects from the most familiar neighborhoods (perhaps because those objects were easier to identify). Alternatively, 12-month-olds may have preferred the same objects in all conditions due to their size, animacy, and/or manipulability. In the revision, we acknowledge these possibilities (see pp. 28-29).

10. *574: Such a design critically depends on the reliability of measurement. Did you assess something like split-half reliability? (I'd recommend the splithalfr package to account for the small number of trials). This is just a comment; no need to address it in this paper.*

Thank you. We appreciate this suggestion and will implement it in our future designs.

Reviewer #2

1. *The key claim in the paper is that at 15 months, infants can form a representation of a never-seen object and learn its name. It does not seem that the experimental procedure offers a direct test of infants' mental representation of a hidden labeled object. During the test phase, both the target and the distractor objects were present on the screen while the infants were prompted to find the target. Fifteen-month-old infants did look at the target more than at the distractor, but this does not specify what they had represented during the absent reference phase or if they had formed a representation of a specific object. In combination with the two control conditions, it is possible to claim that infants at this age rely on the category membership of the objects presented during training and that they also use the novel label to infer that the target is another object from the same category. It is possible that during the absent reference phase, they do activate category-relevant information and bring to mind a concept of a fruit. However, we cannot say that they "form a representation of a never-seen object." I suggest reframing the paper to soften this strong interpretation and making it more about infants' ability to rely on taxonomic information to infer the meaning of a new word.*

Thank you for raising this point. In the revision, we softened the interpretation throughout. But we propose that infants likely establish *some* representation of the unseen object. We know that by 15 months, infants take into account contextual cues to interpret ambiguous requests for hidden objects (Ganea, 2005), rely on language to anticipate changes in recently seen scenes (Ganea et al., 2016), and use the number of unique labels to individuate unseen objects (Xu et al., 2005). Together, these findings suggest that at 15-months, infants can form a placeholder representation of an unseen object, however sparse (e.g., it can be a simple notion that something related to the shown objects is hidden behind the actor), and map a word to it. We added a clarification that our

goal is to assess whether infants can, *in principle*, map novel words onto newly-formed representations of unseen objects (p. 4, lines 71-72). We return to this point in the general discussion to note that in an ongoing study, we are implementing a design that will permit us to characterize the *content* of representations that infants form in the absence of visual referents to novel words (p. 30-31, lines 674-684).

2. *The choice of distractor objects and target-distractor pairing may raise concerns. For example, the target and distractor are not only from different categories, but they also differ in manipulability (ottoman, dragon fruit), animacy (tarsier, rope), and size (regalia, spatula). Without the two control conditions, it would be possible to bring up alternative interpretations of the results. For example, 15-month-olds looked at the tarsier for longer than the rope because animals are more interesting to them than inanimate objects. Infants looked at the dragon fruit for longer because easily graspable, manipulable objects are more appealing to them than pieces of furniture. The two control conditions rule out these explanations, but it would help the reader if this was explicitly stated.*

Thank you for this suggestion. We now mention explicitly that 15-month-olds' looking patterns across the conditions rule out the possibility that the results in the Semantic Priming condition could be due manipulability, animacy or size of target objects (p. 19, lines 431-432).

3. *I also suggest adding more details to the description of the design and procedure. Was the side of the target counterbalanced for each semantic neighborhood? Was the order of the four within-subjects trials counterbalanced?*

We followed this advice and now note that a) we used a Latin-squares design to produce 4 sequences of trial order, with infants assigned randomly to a sequence; b) that the L-R position of the target alternated across trials (left-right-left-right or right-left-right-left; p. 12, lines 256-258). We also report that there was no effect for trial order in either Experiment 1 (p. 14, lines 340-342 or Experiment 2 (p. 23, line 527).

4. *I don't think the term "never-seen object" describes the nature of the target selection very well. We cannot be sure that infants had never seen target objects used in the study before, especially the dragon fruit. It is sold in many grocery stores and infants could have been exposed to it before. It is unlikely that infants know the names for the target objects, and even if some infants are familiar with tarsiers or dragon fruit, it is unlikely to explain the results. Nevertheless, to avoid undue questions, I suggest changing the term "never-seen object" to something like "hidden object," "unseen object," "occluded object," or "perceptually unavailable object."*

Thank you for this insight. We agree and have changed the term to "unseen object" throughout; this terminological change is also consistent with prior literature reporting on infants' comprehension of reference to items or scenes that are not made visible until test trials.

5. *The same applies to the title of the paper. The previous title mentioned in line 120 looks more appropriate and represents what was done in the paper much better than the current one.*

We have changed the title to "Fifteen-month-olds can learn names of unseen objects in their absence" to soften the claim about representations. We favor this title over the one we proposed in the Stage-1 registered report ("Semantic priming supports infants' ability to learn names of unseen objects"), because it communicates the current set of findings and the goal of our investigation.

6. *The introduction can benefit from a more thorough account of previous research concerning the cognitive components required by the procedure. For example, it is worth adding more*

information about infants' and toddlers' conceptual development, their understanding of common semantic neighborhoods, as well as their ability to use this knowledge to identify the referent of a common noun (e.g., Osina, M. A., Saylor, M. M., & Ganea, P. A. (2018). Infants use category label knowledge to interpret absent reference. Infancy, 23(5), 650-673.). There is also classic research on infants' ability to map new words to unseen objects and actions that seems very relevant to the current investigation (Akhtar, N., & Tomasello, M. (1996). Two-year-olds learn words for absent objects and actions. British Journal of Developmental Psychology, 14(1), 79-93.).

We appreciate you reminding us of these excellent, related papers. We include them in the revision, especially the introduction.

7. *The description of the stimuli in the text of the manuscript and in Figure 1 is inconsistent with what appears in the videos posted on OSF. For example, in the videos, the distractor is an ottoman, not a chandelier, and in the semantic neighborhood of animals, the target is a tarsier, not a sugar glider.*

Thank you for your careful attention to this important detail. To conform to the image copyright requirements of this journal, we needed to change which images appear in the Appendix and in some of the figures. The images in the videos accurately reflect the images we used. In the revised manuscript, we have changed the references to objects in the text to match those in the videos.

8. *Experiment 2, 12-month-olds. As the results suggest, vocabulary training did not help the younger group of infants infer the referent of a novel word. Is it possible that the training only helped infants learn isolated words without assisting the formation of semantic* neighborhoods? This could explain why vocabulary training did not support category priming.

Yes, we agree. The training was designed to support infants' memory of priming object names and their ability to recognize these objects as exemplars of their basic-level categories (e.g., apples, oranges, bananas, etc.). The training was indeed not designed to enrich infants' knowledge of semantic neighborhoods. We articulate this both in motivating the design of Experiment 2 and the discussion (p. 28, lines 647-654).

9. *Vocabulary training focused on teaching infants the names of objects used in the priming phase and did not include the names of the distractor items. It may be helpful to remind the reader that infants were not supposed to know the names of the distractors so that they could not rely on mutual exclusivity to detect the target.*

Thank you for mentioning this. We now clarify (p. 22, lines 480-487) that our vocabulary training did include the names of distractor objects on the vocabulary test and did not include the names of either target or distractor objects used in the main experimental trials (baseline and outcome assessments).

10. *One may think that infants could have been biased to look at the target object in all conditions (experimental and control) because it was from the category that infants were exposed to during vocabulary training. However, they also displayed preference for the target at the baseline assessment as well. Can this bias be explained by infants' preference for animals over objects, graspable objects over non-graspable ones, and bigger objects over smaller ones? See comment (2) above.*

Thank you for raising this point. This is indeed possible that object properties (graspable, big, animate, etc.) could have affected infants' looking preferences; we acknowledge this possibility in

the revised manuscript (p. 29, lines 651-654). Vocabulary training, however, is unlikely to have motivated this looking preference. Infants showed the same looking patterns both before (baseline) and after (outcome) the vocabulary training occurred. This suggests that vocabulary training, which exposed infants to word-object pairs drawn from semantic neighborhoods of both target and distractor objects on the main task, did not affect infants' performance on the outcome assessment. Familiarity with semantic neighborhood may have influenced infants' looking preference in a different way. In this design, we selected semantic neighborhoods for priming with which infants are likely already quite familiar (prior to participating in our study). Semantic neighborhoods of distractor objects may have remained less familiar to them, even after vocabulary training. This possibility appears consistent with Pomiechowska & Gliga, 2019.

11. *There are multiple language errors (word choice, articles and prepositions), typos, and missing spaces between citations that need to be corrected before publication.*

Thank you – we have corrected these.

12. *Pages 9 and 19, lines 188 and 422 – make the date format the same.*

Done.

13. *Lines 190-193 in the "Apparatus" section. How was the camera calibration done? I believe it was part of the procedure. Also, a bit more information about how infants' gaze is recorded via web cameras would be helpful.*

We have now provided these details (p. 10, p. 211-222).

14. *Lines 195-200, "Stimuli." From reading the paper for the first time, I found it difficult to follow when the objects were visible and when they were not. It gets clearer later on in the Procedure section (from line 230 on), but I suggest making it clear right away that the objects named in the priming phase appeared behind the actor but were visible to the infant.*

Thank you for pointing this out. We have implemented the suggested edit.

15. *Lines 206-215, "Stimulus selection and design." Specify that the objects presented in the study were pictures, not real objects manipulated by the actor. It is especially important for the conditions where the test phase objects differ in manipulability.*

Done.

16. *Lines 221-227, "Procedure." This section will benefit from a bit more clarity and details. For example, at first, it is confusing what was within subjects and what was between. You can also explain the counterbalancing scheme here.*

Done.

17. *Line 288, the threshold t-value is 1.84, but in line 304 the threshold t-value is mentioned to be 1.691. Why are they different? Readers who are not familiar with this analysis may be confused.*

Thank you for catching this error. The threshold was 1.69. We have now made it consistent.

18. *Line 314, "Predictions." You mention 12-month-olds there although the experiment is about 15-month-olds. To avoid any confusion, I suggest restating that the predictions are about 15-month-olds.*

Done.

19. *Consider citing some literature (lines 314-316) on infants' ability to extend novel count nouns based on category membership to better justify your predictions.*

Done. We have now added several relevant citations (pp. 5-6, lines 109-122).

20. *Results and discussion, line 342. Before describing the results, to improve clarity, please restate the goal of the experiment and the age of the participants.*

Done.

21. *Line 351. When you state that there was no difference between the no priming and switch word conditions, did you switch the reference category when running GLMM to look at this comparison? Consider adding this information.*

Thank you for raising this question. This was done with an additional t-test, which we now state explicitly (p. 18, lines 396).

22. *Figure 3. Indicate when the pictures appear on the screen (right at the offset of the first mentioning of the novel label, correct?).*

Done.

23. *Experiment 2. Lines 399-401. Provide a bit more information here about what vocabulary training entails. One or two sentences with a general description will help improve clarity.*

We have now added more detail (pp. 21-22, lines 473-487).

24. *Vocabulary training, lines 435-437. Please add the names of objects that served as distractors when listing the vocabulary training items drawn from the same semantic neighborhoods.*

Done.

25. *Results and discussion, lines 480-483. I was quite confused by the "negative effect of age." Two different age groups were tested in two separate experiments, so it cannot be a comparison of 12- and 15-month-olds. Do you mean the difference in seven days between the baseline assessment and the outcome assessment? Seven days is barely an effect of age; this is too short of a time period. Maybe you implied baseline to outcome assessment comparison? Please explain. It is also unclear how looking at the interaction could help you understand the main effect of age. This needs a bit of an explanation as well.*

Thank you for pointing it out. The effect of age was within the 12-month-old group. We measured age in days, which we now state explicitly (p. 24, line 526). Infants were about 12 months on average, but there was nearly a month of difference between the youngest and the oldest infants within this age group. We included an interaction because preliminary analyses were conducted for each condition separately (the same variables, such as vocabulary per MCDI, could have influenced the outcome differently across conditions) and the effect of age was only observed in the Semantic Priming condition. Because of this, we included an age-by-condition interaction.

26. *Baseline assessment. Line 486. What is the effect of Intercept? Can you add some information about what it shows? The same applies to line 507.*

We now provide a clarification (p. 18, lines 390-391) that the effect of the intercept refers to the grand mean when all predictors are set at zero.

27. *Figure 5 that follows "Baseline assessment" contains results from both baseline and outcome assessment. When placed right after this section (after line 490), it makes things confusing. Consider moving it below the "Outcome assessment" section.*

Done.

28. *Lines 474, 487. You mention that the distribution of residuals was not normal for the baseline and the outcome assessments. Have you considered transforming the DV? Logarithmic transformation can help normalize residual distribution.*

Thank you for raising this point. We have now provided additional analyses for both baseline and outcome assessments after an exponential transformation of our left-skewed data (p. 24, lines 541-543 and p. 26, lines 569-572).

29. *Despite acknowledging that nonnormal residual distribution rendered significance levels uninterpretable, the authors move on to stating that there was no condition effect. Additionally, different arguments are used to justify this in different sections (see lines 475-476 and 488-489). I suggest further refining the description of the results for Experiment 2 to avoid confusion. Data transformation to normalize residual distribution is one option. Not stating that the significance levels are uninterpretable right before interpreting them is another option.*

Done (see above).

30. *Figure 7. I believe image onset should be after the first word onset, not before, unless the procedure was different for 12-month-olds than for 15-month-olds. Video clips posted on OSF suggest that infants first heard a prompt to find a "blicket" and right after that they were presented with pictures of the test items.*

Done.

31. *Figure 7. Please clarify the reason for showing infants' looking data after 6000 ms. Lines 526-527 suggest that the purpose of this figure was to show that infants have an early bias to look at the target objects.*

We provide the time course for the entire duration of vocabulary test trials, which lasted 10 s (vs. main experimental trials, which lasted 6 s).

32. *Figures 6 and 7. To avoid confusion, I suggest either making the X-axis the same on both figures or explaining the differences. For example, when did the image onset take place? When did the trial actually end?*

These figures (now labeled Figure 7 and Figure 8) represent different types of trials, which we now make clearer both in the text and in captions). Figure 7 represents main experimental trials (baseline and outcome assessments), whereas Figure 8 represents vocabulary test trials, averaged across infants and across semantic neighborhoods (3 items per line)

33. *Discussion, lines 540-541. It is implied here that infants had been introduced to the names of both target and distractor objects and were equally familiar with the images. However, as the description in lines 435-437 suggests, infants were not taught the names of the specific distractor items used in the test phase. Neither did they see those exact pictures. Please work on addressing this discrepancy.*

Thank you for raising this point. We have now clarified (p. 22, lines 480-487) that infants were indeed taught the names of distractor objects used on vocabulary test trials.

34. *General Discussion. Please work on making the discussion consistent with what the experimental methods and results allow concluding (see comment (1)).*

Done.

35. *I believe General Discussion can benefit from citing some literature on infants' category knowledge and novel word extension. Overall, this research could be put in the existing literature on the topic more thoroughly. Here are a couple of examples of relevant research: 1) Pomiechowska, B., & Gliga, T. (2019). Lexical acquisition through category matching: 12-month-old infants associate words to visual categories. Psychological Science, 30(2), 288-299. 2) Booth, A. E., & Waxman, S. R. (2009). A horse of a different color: Specifying with precision infants' mappings of novel nouns and adjectives. Child development, 80(1), 15-22.*

Thank you for these relevant papers, all of which we now include in the General Discussion.

36. *In Appendix 3, the "Switch word" condition is labeled differently. Please make it consistent with the text in the manuscript.*

Done.
