# Supplementary material for: Semantic priming supports infants’ ability to learn names of unseen objects
Source: PLoS One. 2025 Apr 23;20(4):e0321775. doi: 10.1371/journal.pone.0321775 (PMC12017536; doi:10.1371/journal.pone.0321775)
Supplement: Attachment — Submitted filename: Luchkina & Waxman review.docx [file pone.0321775.s006.docx]

Review of the manuscript titled “Fifteen-month-olds represent never-seen objects and learn their names”

Summary.

The paper investigates whether 12- and 15-month-old infants can rely on the category membership of several presented objects to learn the name of an absent object. Infants watched an actor point at and name several pictures of familiar objects, such as fruit. They then heard a name for an object that seemed occluded from view by the actor. When presented with a picture of an unfamiliar object from the same category and a distractor object, infants were asked to find the one labeled by the novel noun. In two control conditions, infants were either presented with objects from different categories during training, or two different labels were used to name the absent object and in the test phase. Infants from the older age group looked at the intended referent more than at the distractor, and this occurred only in the key experimental condition. Infants from the younger group did not show the same preference.

For the most part, this work was carried out during the pandemic, which presented serious challenges to developmental research. The methods used in the study, which allowed remote recording of infants’ looking time, are informative and broaden our understanding of ways to study cognition. The paper also reports a comparison between eye movements recorded remotely using the newly developed software Lookit and those recorded in the lab using the conventional Tobii eye tracker. There is also a comparison between human coders’ annotation of infants’ looking behavior and the same implemented by the automated gaze annotation technology iCatcher. Besides technical innovations, the paper provides insights into infants’ priming abilities, category and word knowledge, and novel label learning at 12 and 15 months. It also adds to the body of research with similar methods, showing that infants’ eye tracking can be recorded via a computer camera without any control from the experimenter. The investigation is well-designed and offers two relevant and well-thought-out control conditions. However, before publication in PLOS ONE, I recommend revising the manuscript to improve the clarity of the methods and procedure, as well as to align the interpretation of the results with the experimental methods.

I. Major Issues.

1. The key claim in the paper is that at 15 months, infants can form a representation of a never-seen object and learn its name. It does not seem that the experimental procedure offers a direct test of infants’ mental representation of a hidden labeled object. During the test phase, both the target and the distractor objects were present on the screen while the infants were prompted to find the target. Fifteen-month-old infants did look at the target more than at the distractor, but this does not specify what they had represented during the absent reference phase or if they had formed a representation of a specific object. In combination with the two control conditions, it is possible to claim that infants at this age rely on the category membership of the objects presented during training and that they also use the novel label to infer that the target is another object from the same category. It is possible that during the absent reference phase, they do activate category-relevant information and bring to mind a concept of a fruit. However, we cannot say that they “form a representation of a never-seen object.” I suggest reframing the paper to soften this strong interpretation and making it more about infants’ ability to rely on taxonomic information to infer the meaning of a new word.
2. The choice of distractor objects and target-distractor pairing may raise concerns. For example, the target and distractor are not only from different categories, but they also differ in manipulability (ottoman, dragon fruit), animacy (tarsier, rope), and size (regalia, spatula). Without the two control conditions, it would be possible to bring up alternative interpretations of the results. For example, 15-month-olds looked at the tarsier for longer than the rope because animals are more interesting to them than inanimate objects. Infants looked at the dragon fruit for longer because easily graspable, manipulable objects are more appealing to them than pieces of furniture. The two control conditions rule out these explanations, but it would help the reader if this was explicitly stated.
3. I also suggest adding more details to the description of the design and procedure. Was the side of the target counterbalanced for each semantic neighborhood? Was the order of the four within-subjects trials counterbalanced?
4. I don’t think the term “never-seen object” describes the nature of the target selection very well. We cannot be sure that infants had never seen target objects used in the study before, especially the dragon fruit. It is sold in many grocery stores and infants could have been exposed to it before. It is unlikely that infants know the names for the target objects, and even if some infants are familiar with tarsiers or dragon fruit, it is unlikely to explain the results. Nevertheless, to avoid undue questions, I suggest changing the term “never-seen object” to something like “hidden object,” “unseen object,” “occluded object,” or “perceptually unavailable object.”
5. The same applies to the title of the paper. The previous title mentioned in line 120 looks more appropriate and represents what was done in the paper much better than the current one.
6. The introduction can benefit from a more thorough account of previous research concerning the cognitive components required by the procedure. For example, it is worth adding more information about infants’ and toddlers’ conceptual development, their understanding of common semantic neighborhoods, as well as their ability to use this knowledge to identify the referent of a common noun (e.g., Osina, M. A., Saylor, M. M., & Ganea, P. A. (2018). Infants use category label knowledge to interpret absent reference. Infancy, 23(5), 650-673.). There is also classic research on infants’ ability to map new words to unseen objects and actions that seems very relevant to the current investigation (Akhtar, N., & Tomasello, M. (1996). Two‐year‐olds learn words for absent objects and actions. British Journal of Developmental Psychology, 14(1), 79-93.).

II. Other Concerns.

7. The description of the stimuli in the text of the manuscript and in Figure 1 is inconsistent with what appears in the videos posted on OSF. For example, in the videos, the distractor is an ottoman, not a chandelier, and in the semantic neighborhood of animals, the target is a tarsier, not a sugar glider.
8. Experiment 2, 12-month-olds. As the results suggest, vocabulary training did not help the younger group of infants infer the referent of a novel word. Is it possible that the training only helped infants learn isolated words without assisting the formation of semantic neighborhoods? This could explain why vocabulary training did not support category priming.
9. Vocabulary training focused on teaching infants the names of objects used in the priming phase and did not include the names of the distractor items. It may be helpful to remind the reader that infants were not supposed to know the names of the distractors so that they could not rely on mutual exclusivity to detect the target.
10. One may think that infants could have been biased to look at the target object in all conditions (experimental and control) because it was from the category that infants were exposed to during vocabulary training. However, they also displayed preference for the target at the baseline assessment as well. Can this bias be explained by infants’ preference for animals over objects, graspable objects over non-graspable ones, and bigger objects over smaller ones? See comment (2) above.
11. There are multiple language errors (word choice, articles and prepositions), typos, and missing spaces between citations that need to be corrected before publication.
12. Pages 9 and 19, lines 188 and 422 – make the date format the same.
13. Lines 190-193 in the “Apparatus” section. How was the camera calibration done? I believe it was part of the procedure. Also, a bit more information about how infants’ gaze is recorded via web cameras would be helpful.
14. Lines 195-200, “Stimuli.” From reading the paper for the first time, I found it difficult to follow when the objects were visible and when they were not. It gets clearer later on in the Procedure section (from line 230 on), but I suggest making it clear right away that the objects named in the priming phase appeared behind the actor but were visible to the infant.
15. Lines 206-215, “Stimulus selection and design.” Specify that the objects presented in the study were pictures, not real objects manipulated by the actor. It is especially important for the conditions where the test phase objects differ in manipulability.
16. Lines 221-227, “Procedure.” This section will benefit from a bit more clarity and details. For example, at first, it is confusing what was within subjects and what was between. You can also explain the counterbalancing scheme here.
17. Line 288, the threshold t-value is 1.84, but in line 304 the threshold t-value is mentioned to be 1.691. Why are they different? Readers who are not familiar with this analysis may be confused.
18. Line 314, “Predictions.” You mention 12-month-olds there although the experiment is about 15-month-olds. To avoid any confusion, I suggest restating that the predictions are about 15-month-olds.
19. Consider citing some literature (lines 314-316) on infants’ ability to extend novel count nouns based on category membership to better justify your predictions.
20. Results and discussion, line 342. Before describing the results, to improve clarity, please restate the goal of the experiment and the age of the participants.
21. Line 351. When you state that there was no difference between the no priming and switch word conditions, did you switch the reference category when running GLMM to look at this comparison? Consider adding this information.
22. Figure 3. Indicate when the pictures appear on the screen (right at the offset of the first mentioning of the novel label, correct?).
23. Experiment 2. Lines 399-401. Provide a bit more information here about what vocabulary training entails. One or two sentences with a general description will help improve clarity.
24. Vocabulary training, lines 435-437. Please add the names of objects that served as distractors when listing the vocabulary training items drawn from the same semantic neighborhoods.
25. Results and discussion, lines 480-483. I was quite confused by the “negative effect of age.” Two different age groups were tested in two separate experiments, so it cannot be a comparison of 12- and 15-month-olds. Do you mean the difference in seven days between the baseline assessment and the outcome assessment? Seven days is barely an effect of age; this is too short of a time period. Maybe you implied baseline to outcome assessment comparison? Please explain. It is also unclear how looking at the interaction could help you understand the main effect of age. This needs a bit of an explanation as well.
26. Baseline assessment. Line 486. What is the effect of Intercept? Can you add some information about what it shows? The same applies to line 507.
27. Figure 5 that follows “Baseline assessment” contains results from both baseline and outcome assessment. When placed right after this section (after line 490), it makes things confusing. Consider moving it below the “Outcome assessment” section.
28. Lines 474, 487. You mention that the distribution of residuals was not normal for the baseline and the outcome assessments. Have you considered transforming the DV? Logarithmic transformation can help normalize residual distribution.
29. Despite acknowledging that nonnormal residual distribution rendered significance levels uninterpretable, the authors move on to stating that there was no condition effect. Additionally, different arguments are used to justify this in different sections (see lines 475-476 and 488-489). I suggest further refining the description of the results for Experiment 2 to avoid confusion. Data transformation to normalize residual distribution is one option. Not stating that the significance levels are uninterpretable right before interpreting them is another option.
30. Figure 7. I believe image onset should be after the first word onset, not before, unless the procedure was different for 12-month-olds than for 15-month-olds. Video clips posted on OSF suggest that infants first heard a prompt to find a “blicket” and right after that they were presented with pictures of the test items.
31. Figure 7. Please clarify the reason for showing infants’ looking data after 6000 ms. Lines 526-527 suggest that the purpose of this figure was to show that infants have an early bias to look at the target objects.
32. Figures 6 and 7. To avoid confusion, I suggest either making the X-axis the same on both figures or explaining the differences. For example, when did the image onset take place? When did the trial actually end?
33. Discussion, lines 540-541. It is implied here that infants had been introduced to the names of both target and distractor objects and were equally familiar with the images. However, as the description in lines 435-437 suggests, infants were not taught the names of the specific distractor items used in the test phase. Neither did they see those exact pictures. Please work on addressing this discrepancy.
34. General Discussion. Please work on making the discussion consistent with what the experimental methods and results allow concluding (see comment (1)).
35. I believe General Discussion can benefit from citing some literature on infants’ category knowledge and novel word extension. Overall, this research could be put in the existing literature on the topic more thoroughly. Here are a couple of examples of relevant research: 1) Pomiechowska, B., & Gliga, T. (2019). Lexical acquisition through category matching: 12-month-old infants associate words to visual categories. Psychological Science, 30(2), 288-299. 2) Booth, A. E., & Waxman, S. R. (2009). A horse of a different color: Specifying with precision infants’ mappings of novel nouns and adjectives. Child development, 80(1), 15-22.
36. In Appendix 3, the “Switch word” condition is labeled differently. Please make it consistent with the text in the manuscript.
