# Supplementary material for: Semantic priming supports infants’ ability to learn names of unseen objects
Source: PLoS One. 2025 Apr 23;20(4):e0321775. doi: 10.1371/journal.pone.0321775 (PMC12017536; doi:10.1371/journal.pone.0321775)
Supplement: Attachment — Submitted filename: Luchkina & Waxman_Revision 2.docx [file pone.0321775.s008.docx]

Review of revision 1 of the manuscript titled *“Fifteen-month-olds can learn names of unseen objects in their absence”.*

I carefully read the revision of the manuscript and found the presentation of the material much improved. However, I still have a few questions and suggestions.

1. Introduction and Discussion. The new version of the manuscript presents a better framing and more appropriate interpretation of the results. However, I am still concerned that some claims are too strong and cannot not be 100% supported by the current design.
   a) I recommend softening or rephrasing the strong assertion about infants' understanding of the referential nature of words. Understanding that words refer rather than “go with” objects, at a minimum, requires understanding that words are linked to mental representations rather than directly to objects, and in the case of common nouns it should be not one specific object but rather a category of objects. I am afraid I am not entirely convinced that the design and the results of the current work provide a direct test of infants’ ability to map words to mental representations or to category of objects. Here is my reasoning.

In the Semantic Priming condition where infants successfully identified the target, the word and objects were presented together during the test at the second naming event and nearly simultaneously at the first naming event (2-second lag). Infants’ understanding of the new word hasn’t been tested in the absence of the target object. Thus, we can’t be sure that the absent reference phase is really necessary for the infants to map the word onto the object. They could have inferred the meaning of the novel label during the test. Moreover, there is no direct assessment of what kind of meaning infants attach to the label of the unseen object while it is still unseen. Without it the claim that infants learn the names of unseen objects is not entirely supported. Although the task is very interesting, it does not provide direct evidence that infants map the novel word onto their representation of the hidden object or that they need this phase of the experiment at all to identify the target.

The comparison between the Semantic Priming and the Switch Word conditions sheds light to an extent on how the absent refence phase and the test work together for infants’ ability to identify the target. We do see that infants need a consistent reference across the absent reference phase and the test to succeed. They could not identify the target when the words were different across the phases of the experiment. Therefore, verbal reference is important, and semantic priming per se can’t explain the results. This, however, does not prove that they need to form a mental representation of an unseen object during the absent reference phase. It is possible that infants failed in the Switch Word condition because they were confused by hearing two labels. They first were invited to “play a game and find a wug” and then after the delay they suddenly had to find the “dawnoo” which could have confused them.

You might argue that in the Semantic Priming condition infants start looking at the target after the first naming event that occurs before the objects actually become visible, so they mapped the word on a mental image of an unseen object first. However, it is possible that the word was still present in infants’ memory when they saw the objects because it was the last one mentioned and with a minimum delay. Therefore, I am not entirely convinced that infants’ success proves that they were relying on their mental representation of the referent and their newly formed concept to map the word onto the now visible object. It does not rule out the possibility that they were just associating words with the objects when both were available.

Besides testing the importance of the representational component of verbal reference, to support the claim about infants’ referential understanding of words you would have to test whether infants just associated the new word with a specific object (e.g., this individual tarsier) or if they indeed generated a conceptual understanding of the word and could identify another tarsier as a “wug”.

Without more evidence that infants relied on mental representations to succeed in this task and formed concepts of new things, a strong claim about infants' understanding of the referential nature of words cannot be made. I don’t think that the claim that infants learn names for unseen objects is supported either. What does it mean “to learn a word” which is a common noun? Is it possible without being able to see the referent? What the study shows is that infants can use category information to infer that the unseen object is another member of that category and rely on the novel word to identify that object during the test. It also shows that infants can categorize an unknown object based on its 2D image.

b) The same concern applies to the claim that the current study demonstrates infants’ understanding of the communicative nature of words. Implementing a control condition that would include using a machine-generated voice instead of an actual human actor, would bolster the argument about infants' grasp of communicative intentions or the communicative nature of words. Without a direct test of infants’ understanding of the communicative nature of words I recommend removing references to it from the Discussion (lines 428, 434).

I understand that at this point it is not feasible to run more experiments for this paper. It already has enough interesting data to warrant publication. I suggest refining the introduction and discussion to better align with the design and findings. Please incorporate the possibilities mentioned in this comment into your introduction and general discussion.

2. The revised title more carefully reflects the intended nature of the experiment, although now it contains a redundancy. The terms “unseen objects” and “objects in their absence” convey the same meaning; thus, I suggest removing “in their absence” from the title. Please also update this in lines 127 and 186. Additionally, considering my earlier comments about the lack of direct evidence that infants relied on mental representations to learn object names, I recommend revising the title entirely.
3. Abstract can be formulated in a more clear and concise way.
   a) E.g., in line 35 you don’t need to state what you predicted. You can focus on summarizing the findings.
   b) In lines 35-37 instead of first describing the purpose of the two control conditions you can directly summarize what they showed. For example, “Two control conditions clarified that infants’ preference for the target could not be explained by the effects of semantic priming alone, regardless of the novel word, or by any intrinsic looking preferences”.
   c) Line 41-42. Just a suggestion: rephrase this sentence to better reflect the novelty of your findings because a large body of previous research already found that “…between 12 and 15 months, infants begin to represent unseen objects.”
   d) Line 32. There is a typo – you probably meant “that” instead of “and” in “…a hidden object from a particular semantic neighborhood (e.g., fruits) **and** was labeled with a novel noun…”
4. Line 151, there is a different format of citation here (Swingley & Aslin, 2000) and it is not included in the references.
5. Line 195 in Methods. “Thirty-one additional participants were excluded from the analyses…” – do you means from your final sample or were they replaced? I think you mean they participated but were excluded from the final analyses resulting in the final sample of 23+23+21 infants. Please make this more clear.
6. Lines 241, 245, 274, 686 – you refer to the target objects as “novel”. Because these objects were not designed specifically for this experiment, and they exist in real life consider replacing “novel” with “unfamiliar”. Additionally, for the same reason, in line 434, “objects they have never seen” should better be replaced by “objects they are unlikely to have seen”.
7. Line 307. You name this variable “Mean %LT to the target” but you use the word “proportion of looking time”. Please make it consistent as either % or proportion throughout the manuscript.
8. Line 333. To what comparisons did you apply the Holm-Bonferroni adjustment? As far as I know, it is usually not applied to coefficients in models with multiple predictors, which in your case are dummy coded.
9. Lines 391-393. Please provide the beta coefficient for the intercept with all the accompanying statistics, like you do for the other two comparisons.
10. Lines 395-396 and 401-402. I recommend changing the reference category to Switch Word condition and then to No Priming conditions in turn to be able to look at 1) the significance of the difference between these conditions and to 2) the difference in %LT in these two conditions to 50% (chance). Because of the differences in how SE are computed, I think, it is better to stay consistent with your type of analysis (GLMM with three dummy coded variables) than to switch to a different one (t-tests).
11. For the statement in lines 495-497 please explain why you decided not to test infants’ knowledge of the distractor objects’ names.
12. Line 527-528. Why is the slope for age negative? Does it mean that with every day increase in age, %LT to target is predicted to decrease? Please provide an interpretation for this result because it is not very intuitive. Also, when you were doing all these preliminary analyses, as far as I understand one factor at a time, did you apply any correction for multiplicity? Could it be that this significant negative coefficient is just a type 1 error?
13. I could not find tables 1-5. They are being referenced in the text and there are captions for them under “Supporting information captions” but I did not find them in the downloadable Appendices, in the text, or on OSF.
14. Lines 548-549. Again, I recommend using GLMM and changing the reference categories in turn to compare %LT in all three conditions to chance. Also, there is a typo: “for” is missing in “preference the target object”.
15. Line 569. “interaction” is missing after “age-by-condition”.
16. Line 572, “distribution” should be “transformation”.
17. Lines 608-611. This sentence has a very complex syntactic structure. It is very difficult to parse it and understand what clause refers to what head. Can you please rephrase it?
18. Line 653, “objects is graspable” – fix the typo.
19. Line 666, “the” is missing between “infer” and “presence”.
20. Lines 685-687. This sentence describes the findings in a very strong way. The results do not demonstrate that infants represent anything specific, any novel unseen objects. Even if they do have a generic placeholder representation this sentence sounds too strong.
21. Lines 687-688. You are referencing “individual differences in infants’ performance on this task reported here”. I don’t think you reported any individual differences, only condition effects and age effects, on average.
22. Please double check all references and citations. I did not check all of them, but I noticed at least 2 inaccuracies. Citation 16 in line 80 should be 15 (Saylor, 2004). Reference to Ferguson et al. (2014) should be 22, not 23.
