# Supplementary material for: Semantic priming supports infants’ ability to learn names of unseen objects
Source: PLoS One. 2025 Apr 23;20(4):e0321775. doi: 10.1371/journal.pone.0321775 (PMC12017536; doi:10.1371/journal.pone.0321775)
Supplement: Attachment — Submitted filename: Stage-2 response to reviewers revision 2 Final.docx [file pone.0321775.s011.docx]

Dear Dr. Morett,

Thank you again. We are grateful to you and the reviewers for your thoughtful feedback on our manuscript, PONE-D-24-17859R1. In the revision, we have addressed reviewers’ remaining concerns and suggestions. Below we provide a point-by-point response to each reviewer’s comments. We hope that you will agree that by responding to this very constructive set of reviews, we have now produced a manuscript that is acceptable for publication in PLOS One.

Sincerely,

Authors

Please note that the line numbers correspond to those in the clean version (not with track changes).

**Reviewer 1**

*This reviewer had only one remaining concern, one that we have attended to carefully. The reviewer noted that it was unclear why our analyses included no random effect for subject in the analysis for %LT.*

We thank R1 for careful attention to our analyses. We agree that in principle, including a grouping variable at the level of randomization would be ideal. Yet here, when we add Participant as another grouping (random) variable in addition to Trial^1^, only a single data point is available to estimate the intercept within each group, and the variance of the intercept is 0. This results in a singular fit error. Even when we remove Trial as a grouping variable, the variance of the intercept due to Participant remains negligible (close to 0), and the model again returns a singular fit error. Moreover, adding or removing grouping variables does not substantially affect our coefficients or their significance (see Table 1 below). We have therefore opted to report the model with Trial (and not Participant) as a grouping variable in manuscript. If you prefer that we include Table 1 as an appendix in the manuscript, we would be happy to do so.

| **Model** | **Condition** | ***Beta*** | ***SE*** | ***T*-value** | ***p*-value** |
| --- | --- | --- | --- | --- | --- |
| Mean %LT ~ Condition+ (1\|Trial) + (1\|Participant) | Intercept | 0.1784 | 0.0446 | 4.00 | .0032 |
| Mean %LT ~ Condition+ (1\|Trial) + (1\|Participant) | No Priming | -0.1784 | 0.0530 | -3.36 | .0010 |
| Mean %LT ~ Condition+ (1\|Trial) + (1\|Participant) | Switch Word | -0.1169 | 0.0468 | -2.50 | .0138 |
| Mean %LT ~ Condition+ (1\|Trial) | Intercept | 0.1784 | 0.0446 | 4.00 | .0032 |
| Mean %LT ~ Condition+ (1\|Trial) | No Priming | -0.1784 | 0.0530 | -3.36 | .0010 |
| Mean %LT ~ Condition+ (1\|Trial) | Switch Word | -0.1169 | 0.0468 | -2.50 | .0138 |
| Mean %LT ~ Condition+ (1\|Participant) | Intercept | 0.1817 | 0.0359 | 5.06 | .0000013 |
| Mean %LT ~ Condition+ (1\|Participant) | No Priming | -0.1888 | 0.0539 | -3.50 | .00063 |
| Mean %LT ~ Condition+ (1\|Participant) | Switch Word | -0.1194 | 0.0478 | -2.50 | .01367 |
| Mean %LT ~ Condition | Intercept | 0.1817 | 0.0359 | 5.06 | .0000013 |
| Mean %LT ~ Condition | No Priming | -0.1888 | 0.0539 | -3.50 | .00063 |
| Mean %LT ~ Condition | Switch Word | -0.1194 | 0.0478 | -2.50 | .01367 |

**Table 1. Coefficients from a GLMM predicting Mean% LT in 15-month-olds**

^1^ Trial is referred to as ‘Test Item’ in the text of the manuscript. Each trial probed a word-image mapping—a test item—from a different semantic neighborhood.

**Reviewer 2**

*This reviewer found the manuscript much improved but mentioned a few remaining questions and suggestions.*

We thank R2 for paying such a close attention to the consistency of our argument and the accuracy of our text. This feedback has been instrumental.

*“I recommend softening or rephrasing the strong assertion about infants’ understanding of the referential nature of words…”*

We thank R2 for this suggestion. Following the suggestion, we removed the paragraph about reference from the introduction. We have also softened our assertion throughout.

*“I am not entirely convinced that the design and the results of the current work provide a direct test of infants’ ability to map words to mental representations or to category of objects…”*

Our design permits us to assess infants’ expectation for the (formerly hidden) referent of the novel word when it becomes visible at Test. As in most analyses based on a word-mapping task at Test, it does not permit us to test the nature or the breadth of infants’ representation of the novel word’s referent. It does, however, provide insight into infants’ ability to map a novel noun to an object unseen prior to the Test phase on the basis of information gleaned earlier (Priming phase) in the absence of any visible referent for that word, and to then to recruit this mapping later at Test. Moreover, we now note (in the GD; lines 691-693) that we are currently testing the breadth of infants’ interpretation of the novel word. Once those data are available, we will have more evidence regarding infants’ ability to link words with kinds, or categories, of unseen objects.

*“In the Semantic Priming condition … infants could have inferred the meaning of the novel label during the test … Moreover, there is no direct assessment of what kind of meaning infants attach to the label of the unseen object while it is still unseen. … Although the task is very interesting, it does not provide direct evidence that infants map the novel word onto their representation of the hidden object or that they need this phase of the experiment at all to identify the target.”*

We agree with R2’s point that our task provides no direct assessment of the kind of representation that infants attach to the novel label of the unseen object during the Priming phase. Instead, we probe infants’ expectations in each condition at Test. We also agree that on the basis of the information provided in the Priming phase, infants could not know which particular member of a semantic neighborhood might be the referent of the novel word until they see the test images. Seeing those objects may indeed have influenced infants’ knowledge by clarifying the mapping.

Nevertheless, to succeed in our task (as they did), infants *must* *have created some representation,* however sparse, that supported their preference for the target object (and not the foil) when it appeared, de novo, at Test. Importantly, the evidence we report here is consistent with the prediction: Infants’ performance at test varies significantly as a function of the information provided during Priming. We now discuss this in the GD (lines 665-676).

*“The comparison between the Semantic Priming and the Switch Word conditions sheds light to an extent on how the absent refence phase and the test work together for infants’ ability to identify the target. We do see that infants need a consistent reference across the absent reference phase and the test to succeed … .”*

We are happy to hear that we have now reported the results in such a way as to clarify this key finding.

*“It is possible that infants failed in the Switch Word condition because they were confused by hearing two labels. They first were invited to “play a game and find a wug” and then after the delay they suddenly had to find the “dawnoo” which could have confused them”*

We now address this possibility in the GD (lines 677-685): “We think there is no reason for such a confusion, unless infants expected continuity of discourse within a trial. If infants expected a correspondence between the pragmatics of the situation—the actor first ostensibly labels a non-visible object and then invites the infant to play a finding game—and the words the actor used, then infants must had realized that the actor was using the novel noun to refer to the hidden object and expected her to continue using that word. This explanation is also consistent with our interpretation that infants formed some representation of the hidden object and linked it the novel word.”

*“You might argue that in the Semantic Priming condition infants start looking at the target after the first naming event that occurs before the objects actually become visible, so they mapped the word on a mental image of an unseen object first. However, it is possible that the word was still present in infants’ memory when they saw the objects because it was the last one mentioned and with a minimum delay.”*

Here, we offer a 2-part response to R2’s point.

First, this would have been a concern if the word-object (i.e. image of the object) mapping was learned in the presence of the object. In that case, retaining the word in the short-term memory may have been sufficient to access a mapping between that word and the object and prompt infants to look to the target object at Test. However, because infants had not seen the target object until it appeared at Test, recalling the word could not have called up a memory of any particular object. Instead, the results indicate that infants connected the word to a more abstract representation.

Second, notice that infants’ looking to the target in the Semantic Priming condition increased after the second mention of the word, paralleling the increase after the first mention (see Figure 3). This provides additional evidence that infants’ looking reflects a word-referent mapping (rather than a carryover activation of a specific semantic field).

*R2 also suggested we revise the title once more to better reflect our findings.*

We have done so. The revised title is “Fifteen-month-olds map novel words to referents that are absent during naming.” We believe that this title accurately reflects our findings.

*R2 suggested that the abstract can be formulated in a more clear and concise way.*

We thank R2 for catching these errors and making helpful suggestions about rewording the abstract. We have implemented the suggested changes.

*“Line 151, there is a different format of citation here (Swingley & Aslin, 2000) and it is not included in the references.”*

We have now made the citation format consistent with the rest of the manuscript and included this citation to the reference list.

*R2 asked that we clarify how many participants were excluded from the final sample.*

We thank R2 for this suggestion. We now include an explicit statement that the 31 additional participants were excluded from the final analyses (lines 185-188).

*R2 suggested that we remove ‘novel’ objects and use ‘unfamiliar’ objects instead to refer to the items we present at Test because there is a chance that infants saw such objects in the past.*

We have implemented both suggested changes throughout the manuscript.

*R2 suggested that we do not use the word proportion in reference to our DV %Mean LT.*

We have changed ‘proportion’ to ‘percent’ throughout.

*R2 raised a question whether the use of Holm-Bonferroni adjustment in a regression model with multiple predictors was justified.*

The use of the adjustment just depends on how you view the hypothesis. It’s either two hypotheses tested in one model or one hypothesis with two coefficients. We originally applied the Holm-Bonferroni adjustment to be conservative and cover both of those interpretations of the hypothesis. In this case, given that the effects of interest are significant with or without the adjustment, we have removed it based on R2’s comment (line 628).

*“Lines 391-393. Please provide the beta coefficient for the intercept with all the accompanying statistics, like you do for the other two comparisons.”*

Done (lines 382-383)

*R2 suggested that we perform comparisons to the chance level by testing the significance of the intercept in models (with each of our conditions treated as the reference level in a separate model) instead of using t-tests.*

We did so for both experiments (lines 387-393, 547-553, and 582-587).

*R2 asked us to explain why we did not test the knowledge of distractor object names in Exp. 2.*

We now provide a statement that we did not test infants’ knowledge of the distractor objects’ names because this knowledge was not relevant to 12-month-olds ability to benefit from semantic priming (lines 491-493).

*R2 asked us to clarify why the slope of age in days in Exp. 2 was negative.*

We now added a statement that the observed effect of age is likely a Type 1 error (line 527). In preliminary analyses, the effects were entered into the same model, which we now state explicitly (lines 375 and 525).

*“I could not find tables 1-5. They are being referenced in the text and there are captions for them under “Supporting information captions” but I did not find them in the downloadable Appendices, in the text, or on OSF.”*

We apologize for this. We will be sure to check if the tables are available in the PDF version of our submission and add them on OSF if necessary.

*R2 also pointed out a number of spelling, punctuation and style errors and suggested better wording in a few places.*

We are grateful to R2 for their careful attention to detail in our manuscript. We have corrected the errors and improved the wording in every case.
