# Supplementary material for: Semantic priming supports infants’ ability to learn names of unseen objects
Source: PLoS One. 2025 Apr 23;20(4):e0321775. doi: 10.1371/journal.pone.0321775 (PMC12017536; doi:10.1371/journal.pone.0321775)
Supplement: Attachment — Submitted filename: Luchkina & Waxman_Revision 3.docx [file pone.0321775.s010.docx]

Review of revision 2 of the manuscript titled *“Fifteen-month-olds map novel words to referents that are absent during naming”.*

I carefully read the second revision of the manuscript and found the presentation of the material further improved. My remaining concerns can truly be addressed by running additional experiments, which of course is not feasible. Therefore, I suggest that the authors explicitly acknowledge all alternative interpretations of their results (as detailed below) and include more thorough and detailed argumentation supporting their default interpretations.

1) Infants may not form any representations of the unseen object during the absent reference phase and wait until the test phase when the objects become visible to infer which one is the referent of the novel word. Without running an additional experiment, I don’t think it is possible to test the idea that infants had to form any kind of “placeholder” representation of an unseen object to succeed at this task. What if the absent reference phase is replaced by a filler phase where the experimenter simply looks at the infant? Will they still be able to find a “wug” at test? It is possible that the results would be the same without the absent reference phase. The current experimental procedure does not allow any assessment of the kind of representation that infants create when the experimenter labels the unseen object or if they create it at all.

It is wonderful that the authors are conducting further research to investigate the breadth of infants’ representations of unseen mentioned objects in such a scenario. However, before new findings are available, there are still alternative accounts of the data presented in this manuscript. The possibility that infants map novel words to the visible object at test rather than to the absent object at the learning phase cannot be ruled out.

2) It is possible that in the switch word condition infants actually did expect a continuity of discourse. They were initially asked to find a “wug”, and when the objects became visible at test, they were suddenly asked to find a “dawnoo”. This may have been confusing and puzzling for them, which potentially explains their behavior in this condition rather than supporting the idea that they had formed a representation of an unseen “wug” in the absent reference phase.

If you believe that infants at 15 months do not have the necessary sensitivity to the pragmatics of the situation in order to expect a continuity of discourse (line 679 in the general discussion) I suggest that you refer to evidence from prior research to support this point. I suggest that a better and more detailed argument should be made to support the interpretation proposed in the manuscript.

Minor points.

1) Lines 665 and 677-678 in the general discussion. I think stylistically it is best not to start a paragraph with a question and rather phrase the first sentence as a statement.
2) Lines 679-683. This sentence is quite long and is difficult to parse.
3) There are still a few typos and grammatical errors, especially in the new text added in the current revision. One example is “infants must **had** realized” in line 681.
4) In the general discussion some sentences contain word repetitions and tautologies (e.g., line 668, “seen scenes”, line 675, the word “hear” used twice). In general, many sentences could be phrased better.
