# Supplementary material for: Semantic priming supports infants’ ability to learn names of unseen objects
Source: PLoS One. 2025 Apr 23;20(4):e0321775. doi: 10.1371/journal.pone.0321775 (PMC12017536; doi:10.1371/journal.pone.0321775)
Supplement: Attachment — Submitted filename: Response to reviewers 3.docx [file pone.0321775.s012.docx]

Dear Dr. Morett,

Thank you again. We are grateful to you and the reviewers for your thoughtful feedback on our manuscript, PONE-D-24-17859R2. In the revision, we have addressed reviewers’ remaining concerns and suggestions. Below we provide a point-by-point response to each reviewer’s comments. We hope that you will agree that by responding to this very constructive set of reviews, we have now produced a manuscript that is acceptable for publication in PLOS One.

Sincerely,

Authors

Please note that the line numbers correspond to those in the clean version (not with track changes).

**Reviewer 1**

*Reviewer 1 asked us to report the results of our regression analyses including Participant as a grouping factor for the intercept.*

We followed R1’s advice and now report the results of our regression analyses in both experiments with Participant *grouping factor* included (lines 397-399, 547-549, and 581-583).

**Reviewer 2**

*Reviewer 2 asked us to explicitly acknowledge alternative interpretations of our results and include more thorough and detailed argumentation supporting their default interpretations.*

We have done so in the General Discussion (lines 682-691) of the revised manuscript. We now explicitly state that the interpretations suggested by R2 are indeed plausible and will be explored in future investigations.

Furthermore, we have edited the manuscript throughout to temper the original claim about infants’ representations of hidden referent and now emphasize that infants may represent something about the meaning of the novel noun, but not necessarily the hidden referent. Instead, we bring up the idea about infants’ representation of the hidden referent of the novel noun in the General Discussion as one of the plausible interpretations of our results. We then mention another alternative, one proposed by R2, as a plausible interpretation of our findings as well.

We also changed the title of the manuscript back to the original one, following R2’s advice.

We have also thoroughly proofread the manuscript and corrected all typos, word repetitions or omissions, and other errors.

We thank you and the reviewers for detailed feedback and advice.
